# Study of the Structure–Activity Relationship of an Anti-Dormant Mycobacterial Substance 3-(Phenethylamino)Demethyl(oxy)aaptamine to Create a Probe Molecule for Detecting Its Target Protein

**DOI:** 10.3390/md20020098

**Published:** 2022-01-24

**Authors:** Yuji Sumii, Kentaro Kamiya, Takehiko Nakamura, Kenta Tanaka, Takumi Kaji, Junya Mukomura, Naoyuki Kotoku, Masayoshi Arai

**Affiliations:** 1 Graduate School of Pharmaceutical Sciences, Osaka University, 1-6 Yamadaoka, Suita, Osaka 565-0871, Japan; sumii.yuji@nitech.ac.jp (Y.S.); k.kami@outlook.com (K.K.); nakamura-take@phs.osaka-u.ac.jp (T.N.); tnkn_yzy_lvmkntoktt@yahoo.co.jp (K.T.); kttk0810@leaf.ocn.ne.jp (T.K.); 2 College of Pharmaceutical Sciences, Ritsumeikan University, 1-1-1 Noji-higashi, Kusatsu, Shiga 525-8577, Japan; ph0107ir@ed.ritsumei.ac.jp

**Keywords:** tuberculosis, dormant, antibiotics, aaptamine, structure–activity relationship, probe molecule

## Abstract

The current tuberculosis treatment regimen is long and complex, and its failure leads to relapse and emergence of drug resistance. One of the major reasons underlying the extended chemotherapeutic regimen is the ability of *Mycobacterium tuberculosis* to attain a dormant state. Therefore, the identification of new lead compounds with chemical structures different from those of conventional anti-tuberculosis drugs is essential. The compound 3-(phenethylamino)demethyl(oxy)aaptamine (PDOA, **1**), isolated from marine sponge of *Aaptos* sp., is known as an anti-dormant mycobacterial substance, and has been reported to be effective against the drug resistant strains of *M. tuberculosis*. However, its target protein still remains unclear. This study aims to clarify the structure–activity relationship of **1** using 15 synthetic analogues, in order to prepare a probe molecule for detecting the target protein of **1**. We succeeded in creating the compound **15** with a photoaffinity group that retained antimicrobial activity, which proved to be a suitable probe molecule for identifying the target protein of **1**.

## 1. Introduction

Tuberculosis, caused by *Mycobacterium tuberculosis*, remains a major infectious disease worldwide. In 2020, the World Health Organization (WHO) had recorded an estimated 10 million cases of tuberculosis and 1.5 million deaths globally [1]. The current standard 6-month regimen, known as Directly Observed Therapy Short-course (DOTS), was first introduced by the WHO. The regimen administers isoniazid, rifampicin, ethambutol, and pyrazinamide for 2 months, followed by isoniazid and rifampicin for 4 months, and is highly effective against the drug-susceptible *M. tuberculosis* [2]. However, the regimen is long and complex, and its failure leads to relapse and the emergence of drug-resistant varieties, namely multidrug-resistant (MDR) and extensively drug-resistant (XDR) ones. One of the major reasons for the extended chemotherapeutic regimen is the ability of *Mycobacterium tuberculosis* to remain in a dormant state [3]. Furthermore, only three new anti-tuberculosis drugs, bedaquiline (TMC207), delamanid (OPC-67683), and pretomanid (PA-824), have been developed and approved in the past 50 years. In addition, there already exist several cases that are resistant to both bedaquiline and delamanid treatment [4,5]. Therefore, the discovery of new lead compounds targeting the mycobacteria would be urgently required to overcome and avoid drug resistance.

The marine environment is a rich source of drug leads, owing to its chemical and biological diversity. Bioactive compounds from marine organisms are also useful tools for identifying novel drug targets. Due to such biodiversity, more than 15 marine-derived drugs have already been approved, and more than 30 compounds are currently in clinical trials [6]. To date, we have explored marine medicinal resources for anti-mycobacterial substances using the fast-growing *Mycobacterium smegmatis* and the slow-growing *Mycobacterium bovis* BCG that are highly homologous to *Mycobacterium tuberculosis*. Moreover, we induced the dormancy response, such as isoniazid tolerance, using hypoxic culture conditions in *M. smegmatis* and *M. bovis* BCG, and used it for screening the anti-dormant mycobacterial substances. Previously, we had isolated some aaptamine-class alkaloids from the Indonesian marine sponge *Aaptos* sp. as anti-dormant mycobacterial substances [7,8]. Among them, 3-(phenethylamino)demethyl(oxy)aaptamine (PDOA, **1**) was found to exhibit a potent anti-microbial activity on *M. bovis* BCG under both actively growing aerobic conditions and dormancy-inducing hypoxic conditions. We succeeded in the total synthesis of compound **1** and clarified the effectiveness of the latter against *M. tuberculosis*, including the clinically isolated MDR and XDR strains [8]. Therefore, PDOA may be a new lead compound for an anti-tuberculosis drug. However, the detailed mode of action and target molecule of compound **1** remain unclear.

A number of methods for identifying target molecules have been reported so far. Among them, an analysis method of target molecules utilizing the synthesized probe molecule is one of the useful methods. For instance, nybomycin, isolated as an anti-dormant mycobacterial substance from marine-derived *Streptomyces* sp., was shown to bind to the mycobacterial genome [9]. Dictyoceratin-C was found to target RNA polymerase II-associated protein 3 (RPAP3) and inhibit the growth of cancer cells selectively under hypoxic conditions [10]. These target molecules were identified using the synthetic probe molecule of nybomycin and dictyoceratin-C.

In this paper, the structure–activity relationship of compound **1** was investigated in order to create a probe molecule for detecting its target. We then evaluate the function of the compound having a photoaffinity group and exhibiting antimicrobial activity as a probe molecule.

## 2. Results and Discussion

### 2.1. Synthesis of PDOA Analogues and Probe Molecules

**Scheme 1:**
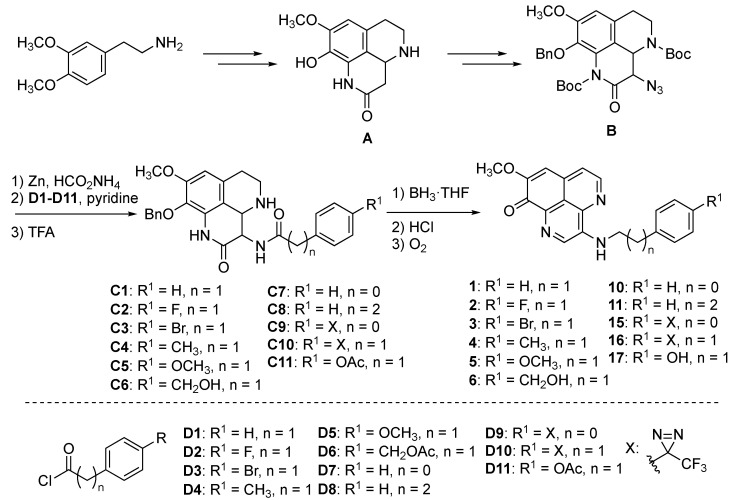
Synthesis of PDOA (**1**) and its analogues.

First, we conducted the synthesis of PDOA analogues by modifying its side chain. Previously, we had accomplished the total synthesis of **1**, the outline of which is depicted in Scheme 1 [8]. Starting with a known tricyclic intermediate **A** [11], an azide group was installed at C-3 position of **A** to produce an intermediate **B**. Then, the phenethylamino side chain was attached through reduction of the azide group and subsequent acylation using acyl chloride **D1** (intermediate **C1**: R^1^ = H, n = 1); the final one-pot sequential transformations, i.e., reduction of amide moieties by BH_3_/hydrolysis of the resulting amine-borane complex/oxidative aromatization using O_2_, produced the desired compound **1**. Applying the above synthetic method with the corresponding acyl chlorides **D2**–**D8**, we could prepare the objective PDOA analogues (**2**–**6**, **10**, **11**) (Figures S1–S14). Compounds **15** and **16**, having a trifluoromethyl diazirine group for the photoaffinity labeling of the target protein, were also synthesized in the same manner as above (Figures S15–S18).

**Scheme 2:**
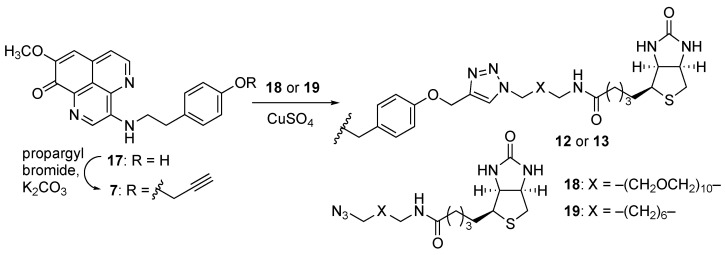
Synthesis of biotinylated probes **12** and **13**.

The biotinylated probes **12** and **13** were prepared as shown in Scheme 2. The compound **17** with the 4-hydroxyphenethyl side chain was prepared through the same procedure as above by using the acyl chloride **D11**, and then reacted with propargyl bromide to produce an *O*-propargyl analogue **7** (Figures S19–S22). Thereafter, Cu(I)-catalyzed Huisgen reaction with polyethylene glycol (PEG)- or alkyl chain-tethered biotin **18** or **19** produced the compounds **12** or **13**, respectively (Figures S23 and S24).

**Scheme 3:**
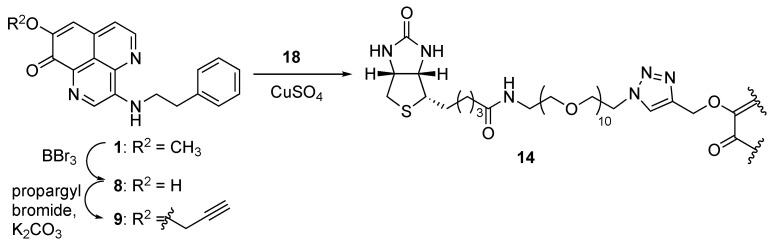
Synthesis of compounds **8**, **9**, and **14**.

Another biotinylated probe **14** was synthesized through the modification of substituent R^2^ of **1** (Scheme 3). The cleavage of the methyl ether at C-8 position of **1** using BBr_3_ produced the 8-OH analogue **8** (Figures S25 and S26). The subsequent propargylation and Huisgen reaction with PEG-tethered biotin **12** produced the desired probe molecule **14** (Figure S29).

### 2.2. Antimicrobial Activity of Compounds on M. bovis BCG

**Figure 1:**
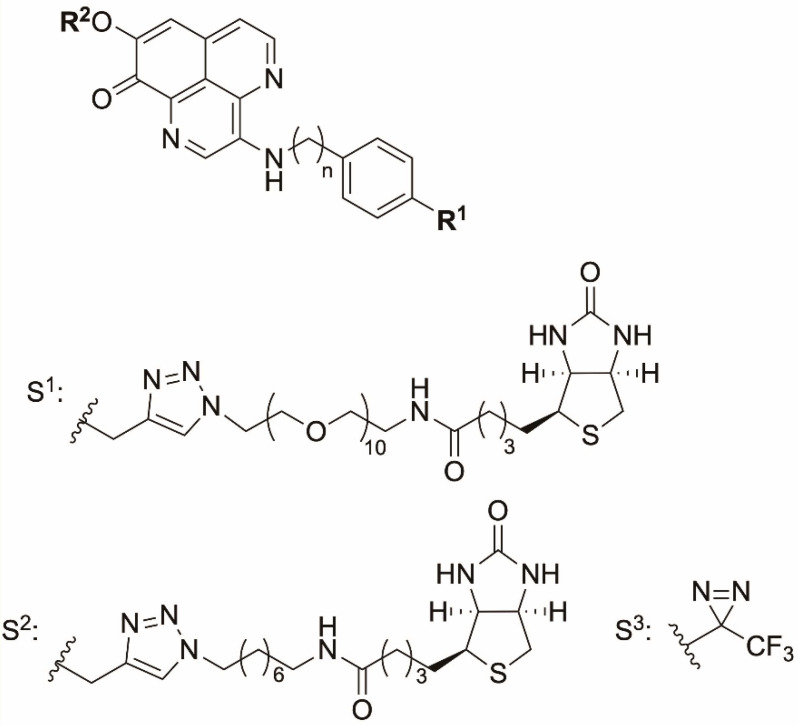
Chemical structures of synthetic analogues of PDOA. The length of the methylene group and functional groups of R^1^ and R^2^ are shown in Table 1.

In order to understand the structure–activity relationship of 3-(phenethylamino)demethyl(oxy)aaptamine (PDOA, **1**) as an anti-dormant mycobacterial substance, we evaluated the antimicrobial activity of synthesized analogues (**2**–**16**) against *M. bovis* BCG under both active growing aerobic conditions and dormancy-inducing hypoxic conditions (Figure 1 and Table 1).

The antimicrobial activity of compounds that were modified in the substituent R^1^, to halogen (**2** and **3**), electron-donating group (**4**, **5** and **7**), or hydroxymethyl group (**6**), was evaluated next. The results show that the halogen and electron-donating groups do not affect the activity and rather retain almost the same antimicrobial activity as of the natural product. Interestingly, the activity of compound **3,** with Br in R^1^ position, slightly increased against *M. bovis* BCG, under hypoxic conditions. On the other hand, the antimicrobial activity was reduced in case of the analogue **6** with the hydroxymethyl group. We next examined the effect of the length of the methylene chain. The antimicrobial activity of compound **10**, in which the length of methylene chain was shortened from two to one, was decreased. The activity of compound **11** with three methylene chains was slightly decreased against *M. bovis* BCG in aerobic conditions; however, there was no significant difference with that of the natural product. We further investigated the effect of the substituent R^2^ on the antimicrobial activity. The results show the modification of R^2^ to have a significant effect on the activity; the latter was significantly reduced in compounds **8**, **9,** and **14**. Therefore, we decided to utilize the R^1^ position to prepare the probe molecule for **1**. Accordingly, we first synthesized the biotinylated probes (**12** and **13**) by utilizing compound **7**, and we investigated the antimicrobial activity. Unfortunately, the compounds did not show activity up to 200 µM. Thus, we next synthesized the compounds **15** and **16**, in which the trifluoromethyl diazirine group was introduced in R^1^ position, and we evaluated their antimicrobial activity. Both compounds showed the MIC values of 1.5 µM, same as that of the natural product, against *M. bovis* BCG under hypoxic conditions, whereas the activities of compounds **15** and **16** against *M. bovis* BCG under aerobic conditions slightly decreased, with MIC values of 6.25 and 12.5 µM, respectively. Unexpectedly, compound **15**, having one methylene chain, showed more potent activity compared to compound **16** having the same methylene length as the natural product; the activity of compound **10** was reduced compared to that of the natural product (**1**).

### 2.3. Functional Evaluation of Compound **15** as a Probe Molecule

**Figure 2:**
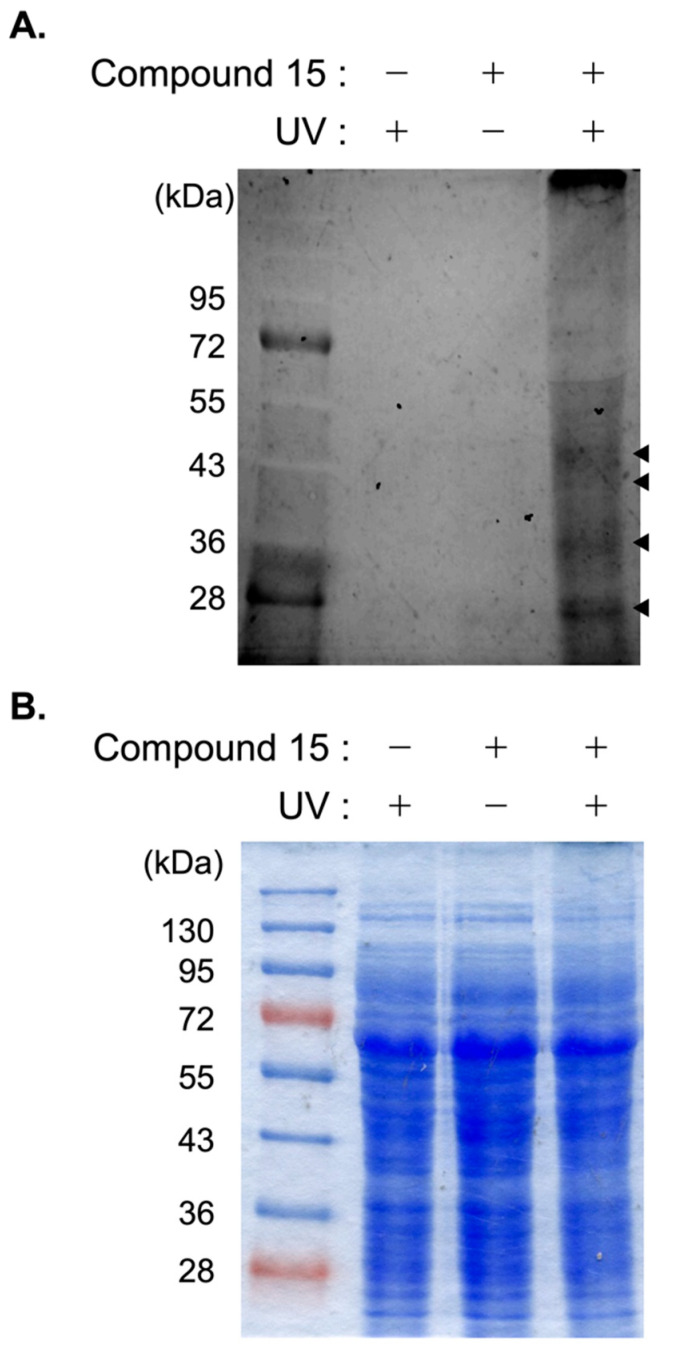
Evaluation of compound **15** as a probe molecule for detecting the target protein. The compound **15** was incubated in the lysate of *M. bovis* BCG for 1 h at 4 °C. After irradiation (365 nm) for 10 min, the proteins in lysate were precipitated by cold acetone. The precipitant was separated by SDS-PAGE. The protein-compound **15** complexes were detected by the fluorescence of compound **15** (Ex: 505 nm, Em: 555 nm) (**A**). The gel was also stained by CBB (**B**). The display of “+” and “−” indicated the presence (+) or absence (−) of compound **15** or UV irradiation.

The aaptamine-class alkaloids are known to be fluorescent [12,13]. Moreover, compound **15**, with trifluoromethyl diazirine moiety at R^1^, retained antimicrobial activity against *M. bovis* BCG under both aerobic and hypoxic conditions with MIC values of 6.25 and 1.56 µM, respectively. Therefore, we speculated that the target protein of compound **1** could be clarified by preparing a covalent bond between compound **15** and target proteins, followed by the detection of fluorescence from compound **15**. We evaluated the function of compound **15** as a probe molecule for detecting the target protein of compound **1**. As shown in Figure 2, three lanes on the acrylamide gel were loaded with the same amounts of protein. No visible band was detected in the fluorescence image under the condition of absence of compound **15** or absence of UV irradiation. However, compound **15** was found to covalently bind to particular proteins under UV-irradiated conditions, which could be detected as four major bands (Figure 2). Although further studies would be necessary for determining the appropriate experimental conditions, the current result strongly suggested compound **15** as a probe molecule that can identify the target protein of compound **1**.

## 3. Materials and Methods

### 3.1. General

The following instruments were used to obtain physical data: a JEOL ECA-500 (^1^H-NMR: 500 MHz, ^13^C-NMR: 125 MHz) spectrometer for ^1^H and ^13^C NMR data, using tetramethylsilane as an internal standard; a JASCO FT/IR-5300 infrared spectrometer for IR spectra; a Waters Q-Tof Ultima API mass spectrometer for ESI-TOF MS; and a Hitachi L-6000 pump equipped with Hitachi L-4000H UV detector for HPLC Silica gel (Kanto 40-100 μm, Nacalai COSMOSIL 75C18-OPN) and pre-coated thin layer chromatography (TLC) plates (Merck 60F_254_, Merck 60RP-18 WF_254_S) were used for column chromatography and TLC, respectively. Spots on the TLC plates were detected by spraying with an acidic *p*-anisaldehyde solution (*p*-anisaldehyde: 25 mL, *c*-H_2_SO_4_: 25 mL, AcOH: 5 mL, EtOH: 425 mL) or with a phosphomolybdic acid solution (phosphomolybdic acid: 25 g, EtOH: 500 mL) with subsequent heating. Unless otherwise noted, all reactions were performed under a N_2_ atmosphere. After the workup, the organic layers were dried over anhydrous Na_2_SO_4_. Chemicals were purchased from Sigma-Aldrich (St. Louis, MO, USA) or Kishida Chemical Co., Ltd. (Osaka, Japan).

### 3.2. Bacterial Culture

*Mycobacterium bovis* BCG Pasteur was grown in Middlebrook 7H9 broth (BD, Franklin lakes, NJ, USA) containing 10% OADC (BD), 0.5% glycerol, and 0.05% Tween 80, or on Middlebrook 7H10 agar (BD) containing 10% OADC and 0.5% glycerol.

### 3.3. Antimicrobial Activity of the Compounds under Aerobic and Hypoxic Conditions

The minimum inhibitory concentrations (MICs) against *M. bovis* BCG Pasteur were determined using the established MTT method [14]. The samples were dissolved in DMSO, and the activity of the samples was evaluated by preparing samples in 2-fold dilution series from 200 uM (final concentration). The mid-log phase of *M. bovis* BCG (1 × 10^5^ CFU/0.1 mL) was inoculated in a 96-well plate, and the serially diluted sample was added to the 96-well plate. In case of aerobic conditions, bacteria were incubated at 37 °C for 7 days. Alternatively, the hypoxic model was established based on the protocol of Rustad et al., with minor modifications [15]. The mycobacterial bacilli were grown in Middlebrook 7H9 broth at 37 °C under a nitrogen atmosphere containing 0.2% oxygen until the optical density at 600 nm reached 0.8. Subsequently, the bacilli were inoculated in a 96-well plate at the same density under aerobic conditions and incubated at 37 °C under a nitrogen atmosphere containing 0.2% oxygen for 14 days. After incubation, an aliquot (50 µL) of MTT solution (0.5 mg/mL) was added to each well and incubated at 37 °C for an additional 12 h under aerobic or hypoxic condition. The optical density at 560 nm was then measured to determine the MIC value. The reproducibility of data was confirmed by three independent experiments.

### 3.4. Synthesis

#### 3.4.1. General Procedure for the Synthesis of PDOA Analogues

##### Procedure A

HCO_2_NH_4_ (10 mmol) and Zn powder (2 mmol) were added to a solution of intermediate **B** (1 mmol) in CH_2_Cl_2_/MeOH (2:1, 10 mL), and the whole mixture was stirred for 5 h. The mixture was filtered through a pad of Celite, and the filtrate was concentrated in vacuo. The residue was dissolved in H_2_O and then basified with 28% aq. NH_3_. The whole mixture was subsequently extracted with CHCl_3_. The removal of solvent from the CHCl_3_ extract under reduced pressure gave a crude amine, which was used for the next reaction without further purification.

Pyridine (10 mmol) and acyl chloride **D** (1.1 mmol) were added to a solution of the above crude amine in CH_2_Cl_2_ (10 mL) and the whole mixture was stirred for 20 h at rt. Sat. NaHCO_3_ aq. was added to the mixture at 0 °C, and the whole mixture was extracted with CH_2_Cl_2_. The removal of solvent from the CH_2_Cl_2_ extract under reduced pressure gave a crude product, which was used for the next reaction without further purification.

TFA (4.0 mL, 50 mmol) was added to a solution of the above product in CH_2_Cl_2_ (10 mL) at 0 °C, and the whole mixture was stirred for 2 h at rt. The solvent was removed from the mixture under reduced pressure; the residue was dissolved in H_2_O and then basified with 28% aq. NH_3_. The whole mixture was extracted with CHCl_3_. The removal of solvent from the CHCl_3_ extract under reduced pressure gave a crude product, which was purified using a SiO_2_ column (CHCl_3_/MeOH = 20:1 containing 1% Et_3_N to 5:1 containing 1% Et_3_N) to give the corresponding intermediate **C**.

##### Procedure B

A BH_3_·THF complex (0.8 mL of a 1.0 M solution in THF, 0.8 mmol, 8.0 equiv.) was added to a solution of the intermediate **C** (0.1 mmol) in anhydrous THF (1 mL) and the whole mixture was stirred at 45 °C for 2 h. MeOH (2 drops) and 6 M aq. HCl were added to the mixture at rt, and the whole mixture was stirred under O_2_ atmosphere at 85 °C for 24 h. The mixture was washed with Et_2_O, and the H_2_O layer was basified with 4 M aq. NaOH. CHCl_3_ was added to the mixture, and the whole mixture was extracted with CHCl_3_-MeOH (10:1). The removal of solvent from the CHCl_3_ extract under reduced pressure gave a crude product, which was purified by SiO_2_ column (CHCl_3_/MeOH = 30:1 + 1% Et_3_N) and ODS column chromatography (70% MeOH) to produce the corresponding PDOA analogues.

The detailed synthetic procedures including synthesis of **D10** (Scheme S1), reaction yields, and physical data of the respective compounds are summarized in the Supplementary Materials (Figures S1–S39).

### 3.5. Functional Evaluation of Compound 15 as a Probe Molecule

A culture of *M. bovis* BCG (500 mL, OD_600_ = 1.0) was centrifuged at 8000× *g* for 20 min. The mycelium was sonicated in 50 mM Tris-HCl (pH 7.4), supplemented with 0.05% Tween 80, and centrifuged (15,000× *g*, 30 min). The protein concentration of the supernatant was adjusted to 1 mg/mL and used for the functional evaluation of compound **15**.

Compound **15** (50 nmol) was added to 1 mg/mL of lysate, and the mixture was incubated at 4 °C for 1 h. After UV irradiation (365 nm) for 10 min on ice, 150 µL of portion was mixed with 600 µL of cold acetone to precipitate the protein in lysate. After centrifugation (15,000× *g*, 60 min), the precipitant was mixed with 1× SDS sample buffer and subjected to SDS-PAGE. Based on the fluorescence of compound **15** (Ex 505 nm, Em 555 nm), compound-**15**-labeled proteins were detected by an Image Quant LAS4010 Scanner (GE Healthcare Life Sciences, Buckinghamshire, UK). To detect whole proteins, the gel was stained by Coomassie Brilliant Blue (CBB).

## 4. Conclusions

Compound **1** (PDOA) is considered to be a new lead compound for anti-tuberculosis drug, although its detailed mode of action and target molecule still remain unclear. Pull-down assays using synthesized probe molecules are highly useful methods for analyzing target molecules of bioactive natural products. To prepare the probe molecule for detecting the target protein of **1**, we investigated the structure–activity relationship of **1** using the synthetic analogues **2**–**16**. The tolerance of the functional group at R^2^ position was found to be low, and OCH_3_ at R^2^ position was revealed to be important for retaining the activity. On the other hand, the tolerance of the functional group at R^1^ position was higher than that at R^2^ position. Based on the information of the structure–activity relationship, we succeeded in creating compound **15** with a photoaffinity group that retained the antimicrobial activity of **1**. We also investigated its function as a probe molecule using its fluorescence properties as an indicator; four bands, derived from the lysate of *M. bovis* BCG, could be detected using compound **15**. Further studies, such as competition experiment with compound **1** to identify the target protein, are under progress.

## Figures and Tables

**Table 1:** Anti-microbial activity of compounds on *M. bovis* BCG under both aerobic and hypoxic conditions.

				MIC (µM)	
Compounds ^1^	R^1^	R^2^	n ^2^	Aerobic	Hypoxic
**1** (PDOA)	H	CH_3_	2	3.13	1.56
**2**	F	CH_3_	2	3.13	1.56
**3**	Br	CH_3_	2	3.13	0.78
**4**	CH_3_	CH_3_	2	3.13	1.56
**5**	OCH_3_	CH_3_	2	3.13	3.13
**6**	CH_2_OH	CH_3_	2	12.5	12.5
**7**	*O*-propargyl	CH_3_	2	3.13	3.13
**8**	H	H	2	50	25
**9**	H	propargyl	2	12.5	6.25
**10**	H	CH_3_	1	12.5	6.25
**11**	H	CH_3_	3	6.25	1.56
**12**	O-S^1^	CH_3_	2	>200	>200
**13**	O-S^2^	CH_3_	2	>200	200
**14**	H	S^1^	2	100	100
**15**	S^3^	CH_3_	1	6.25	1.56
**16**	S^3^	CH_3_	2	12.5	1.56
Isoniazid	-	-	-	0.22	>730

^1^ The structures of the basic skeleton of compounds, namely S^1^, S^2^, and S^3^ are shown in Figure 1. ^2^ “n” indicates the length of methylene group of PDOA analogues in Figure 1.

## Data Availability

Not applicable.

## Supplementary Materials

The following supporting information can be downloaded at https://www.mdpi.com/article/10.3390/md20020098/s1, Scheme S1: Synthesis of diazirine **D10**, Figures S1–S10: ^1^H and ^13^C NMR spectra of **2**–**6**, Figures S11–S14: ^1^H and ^13^C NMR spectra of **10** and **11**, Figures S15–S20: ^1^H and ^13^C NMR spectra of **15**–**17**, Figures S21 and S22: ^1^H and ^13^C NMR spectra of **7**, Figure S23: ^1^H NMR spectrum of **12**, Figure S24: ^1^H NMR spectrum of **13**, Figures S25–S28: ^1^H and ^13^C NMR spectra of **8** and **9**, Figure S29: ^1^H NMR spectrum of **14**, Figures S30 and S31: ^1^H and ^13^C NMR spectra of 1-(4-(2-((*tert*-Butyldimethylsilyl)oxy)ethyl)phenyl)-2,2,2-trifluoroethanone *O*-tosyl oxime, Figures S32 and S33: ^1^H and ^13^C NMR spectra of 3-(4-(2-((*tert*-Butyldimethylsilyl)oxy)ethyl)phenyl)-3-(trifluoromethyl)diaziridine, Figures S34 and S35: ^1^H and ^13^C NMR spectra of 3-(4-(2-((*tert*-Butyldimethylsilyl)oxy)ethyl)phenyl)-3-(trifluoromethyl)-3*H*-diazirine, Figures S36 and S37: ^1^H and ^13^C NMR spectra of 2-(4-(3-(Trifluoromethyl)-3*H*-diazirin-3-yl)phenyl)ethanol, Figures S38 and S39: ^1^H and ^13^C NMR spectra of 2-(4-(3-(Trifluoromethyl)-3*H*-diazirin-3-yl)phenyl)acetic acid.
