# Supplementary material for: Study of the Structure–Activity Relationship of an Anti-Dormant Mycobacterial Substance 3-(Phenethylamino)Demethyl(oxy)aaptamine to Create a Probe Molecule for Detecting Its Target Protein
Source: Mar Drugs. 2022 Jan 24;20(2):98. doi: 10.3390/md20020098 (PMC8879696; doi:10.3390/md20020098)

Supplementary Material for:

Study of the Structure–Activity Relationship of an Anti-Dormant Mycobacterial Substance 3-(Phenethylamino)Demethyl(oxy)aaptamine to Create a Probe Molecule for Detecting Its Target Protein

Yuji Sumii [1,†], Kentaro Kamiya [1], Takehiko Nakamura [1], Kenta Tanaka [1], Takumi Kaji [1], Junya Mukomura [2], Naoyuki Kotoku [1,2,]* and Masayoshi Arai [1,]*

[1] Graduate School of Pharmaceutical Sciences, Osaka University, 1-6 Yamadaoka, Suita, Osaka 565-0871, Japan; sumii.yuji@nitech.ac.jp (Y.S.); k.kami@outlook.com (K.K.); nakamura-take@phs.osaka-u.ac.jp (T.N.); tnkn_yzy_lvmkntoktt@yahoo.co.jp (K.T.); kttk0810@leaf.ocn.ne.jp (T.K.)

[2] College of Pharmaceutical Sciences, Ritsumeikan University, 1-1-1 Noji-higashi, Kusatsu, Shiga 525-8577, Japan; ph0107ir@ed.ritsumei.ac.jp (J.M.)

* Correspondence: kotoku@fc.ritsumei.ac.jp (N.K.); araim@phs.osaka-u.ac.jp (M.A.); Tel: +81-77561-4920 (N.K.); Tel/Fax: +81-66879-8215 (M.A.)

† Current affiliation: Graduate School of Engineering, Nagoya Institute of Technology, Nagoya 466-8555, Japan

Table of contents

Detailed synthetic procedure and physical data of PDOA analogues and probe molecules.

Scheme S1. Synthesis of diazirine **D10**.

^{1}H- and ^{13}C-NMR spectra of PDOA analogues and probe molecules.
Figure S1. ^{1}H NMR spectrum of **2**
Figure S2. ^{13}C NMR spectrum of **2**
Figure S3. ^{1}H NMR spectrum of **3**
Figure S4. ^{13}C NMR spectrum of **3**
Figure S5. ^{1}H NMR spectrum of **4**
Figure S6. ^{13}C NMR spectrum of **4**
Figure S7. ^{1}H NMR spectrum of **5**
Figure S8. ^{13}C NMR spectrum of **5**
Figure S9. ^{1}H NMR spectrum of **6**
Figure S10. ^{13}C NMR spectrum of **6**
Figure S11. ^{1}H NMR spectrum of **10**
Figure S12. ^{13}C NMR spectrum of **10**
Figure S13. ^{1}H NMR spectrum of **11**
Figure S14. ^{13}C NMR spectrum of **11**

Figure S15. ^{1}H NMR spectrum of **15**
Figure S16. ^{13}C NMR spectrum of **15**
Figure S17. ^{1}H NMR spectrum of **16**
Figure S18. ^{13}C NMR spectrum of **16**
Figure S19. ^{1}H NMR spectrum of **17**
Figure S20. ^{13}C NMR spectrum of **17**
Figure S21. ^{1}H NMR spectrum of **7**
Figure S22. ^{13}C NMR spectrum of **7**
Figure S23. ^{1}H NMR spectrum of **12**
Figure S24. ^{1}H NMR spectrum of **13**
Figure S25. ^{1}H NMR spectrum of **8**
Figure S26. ^{13}C NMR spectrum of **8**
Figure S27. ^{1}H NMR spectrum of **9**
Figure S28. ^{13}C NMR spectrum of **9**
Figure S29. ^{1}H NMR spectrum of **14**
Figure S30. ^{1}H NMR spectrum of 1-(4-(2-((*tert*-Butyldimethylsilyl)oxy)ethyl)phenyl)-2,2,2-trifluoroethanone *O*-tosyl oxime
Figure S31. ^{13}C NMR spectrum of 1-(4-(2-((*tert*-Butyldimethylsilyl)oxy)ethyl)phenyl)-2,2,2-trifluoroethanone *O*-tosyl oxime
Figure S32. ^{1}H NMR spectrum of 3-(4-(2-((*tert*-Butyldimethylsilyl)oxy)ethyl)phenyl)-3-(trifluoromethyl)diaziridine
Figure S33. ^{13}C NMR spectrum of 3-(4-(2-((*tert*-Butyldimethylsilyl)oxy)ethyl)phenyl)-3-(trifluoromethyl)diaziridine
Figure S34. ^{1}H NMR spectrum of 3-(4-(2-((*tert*-Butyldimethylsilyl)oxy)ethyl)phenyl)-3-(trifluoromethyl)-3*H*-diazirine
Figure S35. ^{13}C NMR spectrum of 3-(4-(2-((*tert*-Butyldimethylsilyl)oxy)ethyl)phenyl)-3-(trifluoromethyl)-3*H*-diazirine
Figure S36. ^{1}H NMR spectrum of 2-(4-(3-(Trifluoromethyl)-3*H*-diazirin-3-yl)phenyl)ethanol
Figure S37. ^{13}C NMR spectrum of 2-(4-(3-(Trifluoromethyl)-3*H*-diazirin-3-yl)phenyl)ethanol
Figure S38. ^{1}H NMR spectrum of 2-(4-(3-(Trifluoromethyl)-3*H*-diazirin-3-yl)phenyl)acetic acid
Figure S39. ^{13}C NMR spectrum of 2-(4-(3-(Trifluoromethyl)-3*H*-diazirin-3-yl)phenyl)acetic acid

Experimental

General: The following instruments were used to obtain physical data: a JEOL ECS-300 (^{1}H-NMR: 300 MHz, ^{13}C-NMR: 75 MHz), ECA-500 (^{1}H-NMR: 500 MHz, ^{13}C-NMR: 125 MHz) and a Varian NMR system (^{1}H-NMR: 600 MHz, ^{13}C-NMR: 150 MHz) spectrometer for ^{1}H and ^{13}C NMR data using tetramethylsilane as an internal standard; a JASCO FT/IR-5300 infrared spectrometer for IR spectra; a Waters Q-Tof Ultima API mass spectrometer for ESI-TOF MS; HPLC was performed using a Hitachi L-6000 pump equipped with Hitachi L-4000H UV detector. Silica gel (Kanto 40-100 μm, nacalai cosmosil 75C18-OPN) and pre-coated thin layer chromatography (TLC) plates (Merck 60F_{254}, Merck 60RP-18 $WF_{254}S$) were used for column chromatography and TLC. Spots on TLC plates were detected by spraying acidic *p*-anisaldehyde solution (*p*-anisaldehyde: 25 mL, *c*-H_2SO_4: 25 mL, AcOH: 5 mL, EtOH: 425 mL) or phosphomolybdic acid solution (phosphomolybdic acid: 25 g, EtOH: 500 mL) with subsequent heating. Unless otherwise noted, all the reaction was performed under N_2 atmosphere. After workup, the organic layer was dried over Na_2SO_4.

General procedure for the synthesis of PDOA analogues

Procedure A:

HCO_2NH_4 (10 mmol) and Zn powder (2 mmol) were added to a solution of intermediate **B** (1 mmol) in CH_2Cl_2/MeOH (2:1, 10 mL), and the whole mixture was stirred for 5 h. The mixture was filtered through pad of Celite, and the filtrate was concentrated in vacuo. The residue was dissolved in H_2O and was basicified with 28% NH_3 aq. The whole mixture was extracted with $CHCl_3$. Removal of the solvent from the $CHCl_3$ extract under reduced pressure gave a crude amine, which was used for the next reaction without further purification.

Pyridine (10 mmol) and acyl chloride **D** (1.1 mmol) were added to a solution of the above crude amine in CH_2Cl_2 (10 mL) and the whole mixture was stirred for 20 h at rt. Sat. $NaHCO_3$ aq. was added to the mixture at 0 °C, and the whole mixture was extracted with CH_2Cl_2. Removal of the solvent from the CH_2Cl_2 extract under reduced pressure gave a crude product, which was used for the next reaction without further purification.

TFA (4.0 mL, 50 mmol) was added to a solution of the above product in CH_2Cl_2 (10 mL) at 0 °C, and a whole mixture was stirred for 2 h at rt. Solvent was removed from the mixture under reduced pressure, and the residue was dissolved in H_2O and was basicified with 28% NH_3 aq. The whole mixture was extracted with $CHCl_3$. Removal of the solvent from the $CHCl_3$ extract under reduced pressure gave a crude product, which was purified by SiO_2 column ($CHCl_3$/MeOH = 20:1 containing 1% Et_3N to 5:1 containing 1% Et_3N) to give the corresponding intermediate **C**.

Procedure B

$BH_3\cdot$THF complex (0.8 mL, of a 1.0 M solution in THF, 0.8 mmol, 8.0 equiv) was added to a solution of the intermediate **C** (0.1 mmol) in anhydrous THF (1 mL) and the whole mixture was stirred at 45 °C for 2 h. MeOH (2 drops) and 6 M HCl aq. were added to the mixture at rt, and the whole mixture was stirred under

O_2 atmosphere at 85 °C for 24 h. The mixture was washed with Et_2O and H_2O layer was basified with 4 M NaOH aq. $CHCl_3$ was added to the mixture, and the whole mixture was extracted with $CHCl_3$-MeOH (10:1). Removal of the solvent from the $CHCl_3$ extract under reduced pressure gave a crude product, which was purified by SiO_2 column ($CHCl_3$/MeOH = 30:1 + 1% Et_3N) and ODS column chromatography (70% MeOH) to give the corresponding PDOA analogue (**2**~**6**, **10**, **11**, **15**, and **16**) as red solid.

9-(Benzyloxy)-3-((4-fluorophenethyl)amino)-8-methoxy-3a,4,5,6-tetrahydro-1*H*-benzo[de][1,6]-naphthyridin-2(3*H*)-one (C2)

Through Procedure A, **B** (331.4 mg, 0.59 mmol) was converted to **C2** (125.1 mg, 46%) using HCO_2NH_4 (221.8 mg, 3.5 mmol), Zn (76.6 mg, 1.2 mmol), pyridine (0.48 mL, 5.9 mmol) and acyl chloride **D2** (0.088 mL, 0.64 mmol).

^{1}H NMR (500 MHz, $CDCl_3$) δ: 7.60 (1H, d, *J* = 8.1 Hz), 7.51–7.37 (2H, m), 7.36–7.27 (5H, m), 7.22 (1H, s), 6.34 (1H, s), 5.39 (1H, s), 5.26 (1H, d, *J* = 8.0 Hz), 4.95 (2H, s), 4.61 (1H, dd, *J* = 8.0, 4.2 Hz), 4.14 (1H, d, *J* = 4.1 Hz), 3.87 (3H, s), 3.62 (2H, s), 3.18 (1H, dd, *J* = 11.1, 6.5 Hz), 2.95 (1H, td, *J* = 11.7, 3.9 Hz), 2.70–2.57 (1H, m), 2.51 (1H, d, *J* = 14.1 Hz). MS (ESI-TOF) *m/z*: 476 $[M+H]^+$. HRMS (ESI-TOF) *m/z*: 476.1986, calcd for $C_{27}H_{27}N_3O_4F$; Found: 476.1970.

3-((4-Fluorophenethyl)amino)-8-methoxy-9*H*-benzo[de][1,6]naphthyridin-9-one (2)

Through Procedure B, **C2** (41.2 mg, 0.087 mmol)was converted to **2** (10.8 mg, 36%) using BH_3·THF (0.70 mL, 0.70 mmol) and 6 M HCl (1.5 mL).

^{1}H-NMR (500 MHz, $CDCl_3$) δ: 8.73 (1H, d, *J* = 4.6 Hz), 8.39 (1H, s), 7.45 (1H, d, *J* = 4.6 Hz), 7.24–7.22 (2H, m), 7.04–7.01 (2H, m), 6.96 (1H, t-like, *J* = 6.2 Hz), 6.61 (1H, s), 3.97 (3H, s), 3.78 (2H, q, *J* = 6.9 Hz), 3.08 (2H, t, *J* = 6.9 Hz). ^{13}C-NMR (125 MHz, $CDCl_3$) δ: 176.0, 161.9 (1C, d, *J* = 245 Hz), 157.8, 150.8, 143.7, 136.34, 136.27, 124.8, 133.5 (1C, d, *J* = 3.0 Hz), 130.2 (2C, d, *J* = 7.8 Hz), 129.5, 121.6, 117.9, 115.7 (2C, d, *J* = 21.6 Hz), 106.2, 56.1, 44.1, 34.5. IR (KBr): 3368, 1638, 1615, 1563, 1510, 1274, 1203 cm^{-1}. MS (ESI-TOF) *m/z*: 372 $[M+Na]^+$. HRMS (ESI-TOF) *m/z*: 372.1124, calcd for $C_{20}H_{16}FN_3O_2Na$; Found: 372.1111.

***N*-(9-(Benzyloxy)-8-methoxy-2-oxo-2,3,3a,4,5,6-hexahydro-1*H*-benzo[de][1,6]naphthyridin-3-yl)-2-(4-bro**mophenyl)acetamide (**C3**)

Through Procedure A, **B** (502.7 mg, 0.89 mmol) was converted to **C3** (27.8 mg, 58%) using HCO_2NH_4 (560.7 mg, 8.9 mmol), Zn (116.3 mg, 1.8 mmol), pyridine (0.72 mL, 8.9 mmol) and acyl chloride **D3** (0.15 mL, 0.98 mmol).

^{1}H NMR (500 MHz, $CDCl_3$) δ: 7.50 (1H, s), 7.48 (1H, s), 7.47 (1H, s), 7.39–7.29 (5H, m), 7.17 (1H, d, *J* = 8.4 Hz), 7.01 (1H, d, *J* = 8.3 Hz), 6.41 (1H, s), 5.39 (1H, s), 4.99 (1H, dd, *J* = 26.2, 11.1 Hz), 4.74–4.57 (1H, m), 4.19 (1H, d, *J* = 3.9 Hz), 3.93 (3H, s), 3.54 (2H, s), 3.27 (1H, dd, *J* = 11.5, 6.1 Hz), 2.98 (1H, td, *J* = 11.8, 3.9 Hz), 2.85 – 2.68 (1H, m), 2.57 (1H, d, *J* = 16.3 Hz). MS (ESI-TOF) *m/z*: 536 $[M+H]^+$. HRMS (ESI-TOF) *m/z*: 536.1185, calcd for $C_{27}H_{27}N_3O_4Br$; Found: 536.1198.

3-((4-Bromophenethyl)amino)-8-methoxy-9*H*-benzo[de][1,6]naphthyridin-9-one (3)

Through Procedure B, **C3** (46.7 mg, 0.088 mmol) was converted to **3** (12.0 mg, 34%) using BH_3·THF (0.70 mL, 0.70 mmol) and 6 M HCl (1.5 mL).

^{1}H-NMR (500 MHz, $CDCl_3$) δ: 8.75 (1H, d, *J* = 4.6 Hz), 8.40 (1H, s), 7.47-7.46 (3H, m), 7.15 (2H, d, *J* = 8.3 Hz), 6.94 (1H, t, *J* = 6.6 Hz), 6.62 (1H, s), 3.97 (3H, s), 3.79 (2H, q, *J* = 7.0 Hz), 3.07 (2H, t, *J* = 7.0 Hz). ^{13}C-NMR (125 MHz, $CDCl_3$) δ: 176.0, 157.8, 150.9, 143.7, 136.8, 136.4, 136.3, 134.9, 132.0 (2C), 130.5 (2C), 129.5, 121.6, 120.9, 117.9, 106.2, 56.1, 43.8, 34.8. IR (KBr): 3363, 1563, 1511, 1274 cm^{-1}. MS (ESI-TOF) *m/z*: 432/434 $[M+Na]^+$. HRMS (ESI-TOF) *m/z*: 432.0324, calcd for $C_{20}H_{16}{}^{79}BrN_3O_2Na$; Found: 432.0331.

***N*-(9-(Benzyloxy)-8-methoxy-2-oxo-2,3,3a,4,5,6-hexahydro-1*H*-benzo[de][1,6]naphthyridin-3-yl)-2-(*p*-tolyl)acetamide (C4)**

Through Procedure A, **B** (300 mg, 0.53 mmol) was converted to **C4** (53.6 mg, 21%) using HCO_2NH_4 (334 mg, 5.3 mmol), Zn (69 mg, 1.1 mmol), pyridine (0.43 mL, 5.3 mmol) and acyl chloride **D4** (98 mg, 0.58 mmol).

^{1}H NMR (500 MHz, $CDCl_3$) δ: 7.71 (1H, s), 7.41–7.27 (5H, m), 7.09–6.91 (4H, m), 6.38 (1H, s), 5.90 (1H, brs), 5.02 (1H, d, *J* = 16.8 Hz,), 4.92 (1H, d, *J* = 16.8 Hz), 4.72 (1H, dd, *J* = 8.0, 4.2 Hz), 4.22 (1H, d, *J* = 3.8 Hz), 3.85 (3H, s), 3.44 (2H, t, *J* = 7.8 Hz), 3.36–2.48 (4H, m), 2.25 (3H, s).

8-Methoxy-3-((4-methylphenethyl)amino)-9*H*-benzo[de][1,6]naphthyridin-9-one (4)

Through Procedure B, **C4** (52.4 mg, 0.11 mmol) was converted to **4** (4.5 mg, 12%) using BH_3·THF (0.88 mL, 0.88 mmol) and 6 M HCl (1.5 mL).

^{1}H NMR (500 MHz, $CDCl_3$) δ: 8.74 (1H, d, *J* = 4.5 Hz), 8.42 (1H, brs), 7.46 (1H, d, *J* = 4.5 Hz), 7.16 (4H, q, *J* = 8.2 Hz), 7.05 (1H, brs), 6.61 (1H, s), 3.97 (3H, s), 3.78 (2H, q, *J* = 7.0 Hz), 3.07 (2H, t, *J* = 7.0 Hz), 2.33 (3H, s). ^{13}C NMR (150 MHz, $CDCl_3$) δ 175.9, 157.8, 150.7, 144.0, 136.6, 136.2, 136.2, 134.7, 134.5, 129.7, 129.6, 128.6, 121.7, 117.9, 106.2, 56.1, 44.3, 34.9, 21.1. IR (KBr): 3625, 3020, 1563, 1216, 759, 671 cm^{-1}. MS (ESI-TOF) *m/z*: 368 $[M+Na]^+$. HRMS (ESI-TOF) *m/z*: 368.1375, calcd for $C_{21}H_{19}N_3O_2Na$; Found: 368.1362.

***N*-(9-(Benzyloxy)-8-methoxy-2-oxo-2,3,3a,4,5,6-hexahydro-1*H*-benzo[de][1,6]naphthyridin-3-yl)-2-(4-methoxyphenyl)acetamide (C5)**

Through Procedure A, **B** (110.5 mg, 0.20 mmol) was converted to **C5** (53.7 mg, 56%) using HCO_2NH_4 (123.3 mg, 2.0 mmol), Zn (25.6 mg, 0.40 mmol), pyridine (0.16 mL, 0.20 mmol) and acyl chloride **D5** (0.033 mL, 0.22 mmol).

^{1}H NMR (500 MHz, $CDCl_3$) δ: 7.45 (1H, brs), 7.40–7.29 (5H, m), 7.01 (1H, d, *J* = 8.5 Hz), 6.71 (1H, d, *J* = 8.5 Hz), 6.37 (1H, s), 5.33 (1H, d, *J* = 8.2 Hz), 4.97 (1H, d, *J* = 11.0 Hz), 4.90 (1H, d, *J* = 11.0 Hz), 4.65 (1H, dd, *J* = 8.1, 3.9 Hz), 4.16 (1H, d, *J* = 3.9 Hz), 3.90 (3H, s), 3.74 (3H, s), 3.44 (2H, q, *J* = 16.2 Hz), 3.23 (1H, dd, *J* = 12.0, 4.5 Hz), 2.97 (1H, td, *J* = 11.8, 3.8 Hz), 2.69 (1H, m), 2.53 (1H, d, *J* = 14.8 Hz), 1.89 (1H, brs). ^{13}C NMR (150 MHz, $CDCl_3$) δ 171.6, 166.1, 158.6, 152.0, 136.7, 131.7, 130.5, 130.4, 128.6,

128.0, 126.6, 114.4, 114.0, 106.9, 75.1, 56.0, 55.2, 53.6, 52.0, 42.9, 42.0, 30.0, 28.1. IR (KBr): 3393, 3020, 2400, 1700, 1513, 1216, 759 cm^{-1}. MS (ESI-TOF) *m/z*: 510 $[M+Na]^+$. HRMS (ESI-TOF) *m/z*: 510.2005, calcd for $C_{28}H_{29}N_3O_5Na$; Found: 510.1992.

8-Methoxy-3-((4-methoxylphenethyl)amino)-9*H*-benzo[de][1,6]naphthyridin-9-one (5)

Through Procedure B, **C5** (28.6 mg, 0.059 mmol) was converted to **5** (9.0 mg, 42%) using BH_3·THF (0.47 mL, 0.47 mmol), and 6 M HCl (1.5 mL).

^{1}H-NMR (500 MHz, $CDCl_3$) δ: 8.74 (1H, d, *J* = 4.6 Hz), 8.40 (1H, s), 7.45 (1H, d, *J* = 4.6 Hz), 7.20 (2H, d, *J* = 9.3 Hz), 7.00 (1H, t-like, *J* = 6.2 Hz), 6.88 (2H, d, *J* = 9.3 Hz), 6.61 (1H, s), 3.97 (3H, s), 3.80 (3H, s), 3.76 (2H, q, *J* = 7.0 Hz), 3.05 (2H, t, *J* = 7.0 Hz). ^{13}C-NMR (125 MHz, $CDCl_3$) δ: 176.0, 158.6, 157.8, 150.7, 143.9, 136.30, 136.26, 134.6, 129.8, 129.7 (2C), 129.6, 121.6, 117.9, 114.3 (2C), 106.2, 56.0, 55.3, 44.4, 34.5. IR (KBr): 3365, 2927, 1615, 1563, 1541, 1272 cm^{-1}. MS (ESI-TOF) *m/z*: 384 $[M+Na]^+$. HRMS (ESI-TOF) *m/z*: 384.1324, calcd for $C_{21}H_{19}N_3O_3Na$; Found: 384.1331.

4-(2-((9-(Benzyloxy)-8-methoxy-2-oxo-2,3,3a,4,5,6-hexahydro-1*H*-benzo[de][1,6]naphthyridin-3-yl)amino)-2-oxoethyl)benzyl acetate (C6)

Through Procedure A, **B** (456.4 mg, 0.81 mmol) was converted to **C6** (244.2 mg, 57%) using HCO_2NH_4 (509.1 mg, 8.1 mmol), Zn (105.5 mg, 1.6 mmol), pyridine (0.65 mL, 8.1 mmol) and acyl chloride **D6** (201.3 mg, 0.89 mmol).

^{1}H NMR (300 MHz, $CDCl_3$) δ: 7.46 (1H, s), 7.42–7.27 (5H, m), 7.17 (4H, m), 6.39 (1H, s), 5.38 (1H, s), 4.97 (1H, s), 4.68 (1H, dd, *J* = 8.2, 3.7 Hz), 4.17 (1H, d, *J* = 4.1 Hz), 3.90 (3H, s), 3.88 (2H, s), 3.50 (2H, d, *J* = 1.8 Hz), 3.28–3.17 (1H, m), 2.97 (1H, td, *J* = 11.6, 3.8 Hz), 2.83–2.64 (1H, m), 2.54 (1H, d, *J* = 12.6 Hz), 2.10 (3H, s), 1.76 (1H, brs).

3-((4-(Hydroxymethyl)phenethyl)amino)-8-methoxy-9*H*-benzo[de][1,6]naphthyridin-9-one (6)

Thruogh Procedure B, **C6** (33.4 mg, 0.063 mmol) was converted to **6** (2.8 mg, 12%) using BH_3·THF (0.63 mL, 0.63 mmol) and 6 M HCl (1.5 mL).

^{1}H-NMR (500 MHz, $CDCl_3$) δ: 8.72 (1H, d, *J* = 4.6 Hz), 8.34 (1H, s), 7.44 (1H, d, *J* = 4.6 Hz), 7.34 (2H, d, *J* = 7.2 Hz), 7.27 (2H, d, *J* = 7.2 Hz), 6.99 (1H, t, *J* = 5.7 Hz), 6.60 (1H, s), 4.69 (2H, s), 3.96 (3H, s), 3.79 (2H, q, *J* = 7.0 Hz), 3.10 (2H, t, *J* = 7.0 Hz). ^{13}C-NMR (125 MHz, $CDCl_3$) δ: 176.0, 157.8, 150.7, 143.9, 139.7, 137.3, 136.3, 136.2, 134.6, 129.5, 128.9 (2C), 127.6 (2C), 121.6, 117.9, 106.2, 65.0, 56.1, 44.2, 35.1. IR (KBr): 3364, 2930, 1563, 1273 cm^{-1}. MS (ESI-TOF) *m/z*: 384 $[M+Na]^+$. HRMS (ESI-TOF) *m/z*: 384.1324, calcd for $C_{21}H_{19}N_3O_3Na$; Found: 384.1320.

3-(Benzylamino)-9-(benzyloxy)-8-methoxy-3a,4,5,6-tetrahydro-1*H*-benzo[de][1,6]naphthyridin-2(3H)-one (C7)

Through Procedure A, **B** (310.4 mg, 0.55 mmol) was converted to **C7** (211 mg, 87%) using HCO_2NH_4 (207.7 mg, 3.3 mmol), Zn (71.8 mg, 1.1 mmol), pyridine (0.44 mL, 5.5 mmol) and acyl chloride **D7** (0.070 mL, 0.60 mmol).

^{1}H NMR (500 MHz, $CDCl_3$) δ: 7.88–7.77 (1H, m), 7.65–7.06 (10H, m), 6.45 (1H, s), 5.98–5.86 (1H, m), 5.08–4.86 (2H, m), 4.32–4.25 (1H, m), 3.92 (3H, s), 3.82–3.76 (1H, m), 3.76–3.68 (1H, m), 3.57–3.46 (1H, m), 3.44–2.56 (1H, m). ^{13}C NMR (151 MHz, $CDCl_3$) δ 168.0, 165.9, 152.2, 136.5, 133.9, 131.7, 131.4, 131.3, 128.8, 128.6, 128.5, 128.5, 128.4, 128.3, 127.6, 127.4, 107.0, 102.3, 74.9, 74.7, 55.9, 55.7, 53.5, 52.0, 43.0, 29.7, 29.4, 29.3, 27.9. IR (KBr): 3392, 3019, 1698, 1514, 1352 , 1216, 755 cm^{-1}. MS (ESI-TOF) *m/z*: 444 $[M+H]^+$. HRMS (ESI-TOF) *m/z*: 444.1923, calcd for $C_{26}H_{26}N_3O_4$; Found: 444.1939.

3-(Benzylamino)-8-methoxy-9*H*-benzo[de][1,6]naphthyridin-9-one (10)

Through Procedure B, **C7** (71.4 mg, 0.16 mmol) was converted to **10** (9.2 mg, 18%) using BH_3·THF (1.6 mL, 1.6 mmol) and 6 M HCl (1.5 mL).

^{1}H-NMR (500 MHz, $CDCl_3$) δ: 8.77 (1H, d, *J* = 4.3 Hz), 8.42 (1H, s), 7.47 (1H, d, *J* = 4.3 Hz), 7.43-7.37 (4H, m), 7.34-7.33 (1H, m), 6.62 (1H, s), 4.74, (1H, s), 4.72 (1H, s), 3.97 (3H, s). ^{13}C-NMR (125 MHz, $CDCl_3$) δ: 176.1, 157.8, 150.9, 143.7, 136.6, 136.4 (2C), 135.0, 130.0, 129.1 (2C), 128.1, 127.6 (2C), 121.6, 117.9, 106.3, 56.1, 47.2. IR (KBr): 3371, 1615, 1561, 1542, 1272, 1202 cm^{-1}. MS (ESI-TOF) *m/z*: 340 $[M+Na]^+$. HRMS (ESI-TOF) *m/z*: 340.1062, calcd for $C_{19}H_{15}N_3O_2Na$; Found: 340.1051.

***N*-(9-(Benzyloxy)-8-methoxy-2-oxo-2,3,3a,4,5,6-hexahydro-1*H*-benzo[de][1,6]naphthyridin-3-yl)-3-phenylpropanamide (C8)**

Through Procedure A, **B** (373.0 mg, 0.66 mmol) was converted to **C8** (65.0 mg, 21%) using HCO_2NH_4 (416.2 mg, 6.6 mmol), Zn (88.3 mg, 1.4 mmol), pyridine (41.1 μL, 0.51 mmol) and acyl chloride **D8** (9.4 mg, 0.102 mmol).

MS (ESI-TOF) *m/z*: 494 $[M+Na]^+$. HRMS (ESI-TOF) *m/z*: 494.2056, calcd for $C_{28}H_{29}N_3O_4Na$; Found: 494.2078.

8-Methoxy-3-((3-phenylpropyl)amino)-9*H*-benzo[de][1,6]naphthyridin-9-one (11)

Through Procedure B, **C8** (52.0 mg, 0.11 mmol) was converted to **11** (16.9 mg, 44%) using BH_3·THF (1.1 mL, 1.1 mmol) and 6 M HCl (1.5 mL).

^{1}H-NMR (500 MHz, $CDCl_3$) δ: 8.73 (1H, d, *J* = 4.6 Hz), 8.30 (1H, s), 7.44 (1H, d, *J* = 4.6 Hz), 7.31-7.30 (2H, m), 7.22-7.21 (3H, m), 6.95 (1H, t, *J* = 6.3 Hz), 6.60 (1H, s), 3.96 (3H, s), 3.54 (2H, q, *J* = 6.8 Hz), 2.82 (2H, t, *J* = 7.6 Hz), 2.18-2.13 (2H, m). ^{13}C-NMR (125 MHz, $CDCl_3$) δ: 175.9, 157.8, 150.6, 144.0, 140.6, 136.2, 136.1, 134.4, 129.5, 128.6 (2C), 128.3 (2C), 126.3, 121.6, 117.8, 106.1, 56.0, 42.1, 33.0, 30.5. IR (KBr): 3370, 1615, 1563, 1543, 1273, 1204 cm^{-1}. MS (ESI-TOF) *m/z*: 368 $[M+Na]^+$. HRMS (ESI-TOF) *m/z*: 368.1375, calcd for $C_{21}H_{19}N_3O_2Na$; Found: 368.1366.

8-Methoxy-3-((4-(3-(trifluoromethyl)-3*H*-diazirin-3-yl)benzyl)amino)-9*H*-benzo[*de*][1,6]naphthyridin-9-one (15)

Through Procedures A and B, **B** (125.6 mg, 0.222 mmol) was converted to **15** (3.6 mg, 4%).

^{1}H-NMR (500 MHz, $CDCl_3$) δ: 8.79 (1H, d, *J* = 4.6 Hz), 8.34 (1H, s), 7.48-7.46 (3H, m), 7.22-7.20 (2H, m), 6.63 (1H, s), 4.76 (1H, s), 4.75 (1H, s), 3.97 (3H, s). ^{13}C-NMR (125 MHz, $CDCl_3$) δ: 176.1, 157.7, 151.1,

149.1, 143.4, 138.5, 136.5, 135.4, 129.8, 129.0, 127.9 (2C), 127.2 (2C), 121.7, 117.8, 106.3, 56.1, 46.5 (CF_3 and CNN carbons were not observed). IR (KBr): 3362, 1616, 1578, 1561, 1274, 1190, 1154 cm^{-1}. MS (ESI-TOF) *m/z*: 448 $[M+Na]^+$. HRMS (ESI-TOF) *m/z*: 448.0997, calcd for $C_{21}H_{14}F_3N_5O_2Na$; Found: 448.0995.

***N*-(9-(benzyloxy)-8-methoxy-2-oxo-2,3,3a,4,5,6-hexahydro-1*H*-benzo[*de*][1,6]naphthyridin-3-yl)-2-(4-(3-(trifluoromethyl)-3*H*-diazirin-3-yl)phenyl)acetamide (C10)**

Through procedure A, **B** (1.29 g, 2.29 mmol) was converted to **C10** (458 mg, 35%) using NH_4HCO_2 (865 mg, 13.7 mmol), Zn (299 mg, 4.57 mmol), pyridine (1.85 mL, 22.9 mmol) and acyl chloride **D10** (2.53 mmol).

^{1}H-NMR (500 MHz, $CDCl_3$) δ: 7.48 (1H, s), 7.34-7.32 (4H, m), 7.19-7.17 (3H, m), 7.04 (2H, d, *J* = 8.0 Hz), 6.39 (1H, s), 5.38 (1H, br s), 5.25 (1H, d, *J* = 8.0 Hz), 4.99 (2H, s), 4.63 (1H, dd, *J* = 8.0, 4.0 Hz), 4.16 (1H, d, *J* = 4.0 Hz), 3.93 (3H, s), 3.52 (1H, d, *J* = 15.5 Hz), 3.46 (1H, d, *J* = 15.5 Hz), 3.20 (1H, ddd, *J* = 11.9, 5.9, 1.6 Hz), 2.97 (1H, td, *J* = 11.7, 4.4 Hz), 2.63 (1H, dt, *J* = 18.3, 4.7 Hz), 2.54 (1H, dd, *J* = 16.0, 2.9 Hz). ^{13}C-NMR (125 MHz, $CDCl_3$) δ: 170.2, 166.1, 151.9, 136.7, 136.5, 131.8, 131.5, 129.7 (2C), 128.6 (2C), 128.5 (2C), 127.9, 127.8, 126.9, 126.6 (2C), 122.0 (1C, q, *J* = 275 Hz), 111.1, 106.8, 75.0, 55.8, 53.6, 52.2, 42.88, 42.86, 28.26, 28.19 (1C, q, *J* = 40.4 Hz). IR (KBr): 3282, 3033, 2939, 1687, 1517, 1348, 1232, 1186, 1154 cm^{-1}. MS (ESI-TOF) *m/z*: 566 $[M+H]^+$. HRMS (ESI-TOF) *m/z*: 566.2015, calcd for $C_{22}H_{16}F_3N_5O_2Na$; Found: 566.2006.

8-Methoxy-3-((4-(3-(trifluoromethyl)-3*H*-diazirin-3-yl)phenethyl)amino)-9*H*-benzo[*de*][1,6]naphthyridin-9-one (16)

Through Procedure B, **C10** (156 mg, 0.275 mmol) was converted to **16** (7.2 mg, 6%) using $BH_3{\cdot}THF$ (2.75 mL, 2.75 mmol) and 6 M HCl (5 mL).

^{1}H-NMR (500 MHz, $CDCl_3$) δ: 8.74 (1H, d, *J* = 4.6 Hz), 8.39 (1H, s), 7.46 (1H, d, *J* = 4.6 Hz), 7.31 (2H, d, *J* = 8.3 Hz), 7.17 (2H, d, *J* = 8.3 Hz), 6.93 (1H, t-like, *J* = 6.3 Hz), 6.61 (1H, s), 3.97 (3H, s), 3.79 (2H, q, *J* = 7.0 Hz), 3.12 (2H, t, *J* = 7.0 Hz). ^{13}C-NMR (125 MHz, $CDCl_3$) δ: 176.0, 157.8, 150.9, 143.6, 139.7, 136.4, 136.3, 134.9, 129.4, 129.2 (2C), 127.8, 127.0 (2C), 122.1 (CF_3, 1C, q, *J* = 275 Hz), 121.7, 117.9, 106.3, 56.1, 43.8, 34.9, 28.3 (CN=N, 1C, q, *J* = 40.8 Hz). IR (KBr): 2917, 1614, 1565, 1345, 1274, 1182, 1154 cm^{-1}. MS (ESI-TOF) *m/z*: 462 $[M+Na]^+$. HRMS (ESI-TOF) *m/z*: 462.1154, calcd for $C_{22}H_{16}F_3N_5O_2Na$; Found: 462.1167.

4-(2-((9-(Benzyloxy)-8-methoxy-2-oxo-2,3,3a,4,5,6-hexahydro-1*H*-benzo[de][1,6]naphthyridin-3-yl)amino)-2-oxoethyl)phenyl acetate (C11)

Through Procedure A, **B** (476.3 mg, 0.84 mmol) was converted to **C11** (195 mg, 45%) using NH_4HCO_2 (531.3 mg, 8.4 mmol), Zn (110.1 mg, 1.7 mmol), pyridine (0.68 mL, 8.4 mmol), acyl chloride **D11** (197.1 mg, 0.93 mmol).

^{1}H NMR (500 MHz, $CDCl_3$) δ: 7.47 (1H, s), 7.39–7.30 (5H, m), 7.16 (2H, d, *J* = 8.4 Hz), 6.97–6.92 (2H, m), 6.40 (1H, s), 5.49–5.32 (1H, m), 5.00 (2H, s), 4.69 (1H, dd, *J* = 7.9, 4.2 Hz), 4.18 (1H, d, *J* = 4.0 Hz), 3.90

(3H, s), 3.50 (2H, d, J = 5.7 Hz), 3.30–3.18 (1H, m), 2.97 (1H, td, J = 11.8, 3.8 Hz), 2.83–2.69 (1H, m), 2.54 (1H, d, J = 14.1 Hz), 2.28 (3H, s). MS (ESI-TOF) *m/z*: 538 $[M+Na]^+$. HRMS (ESI-TOF) *m/z*: 538.1954, calcd for $C_{29}H_{29}N_3O_6Na$; Found: 538.1937.

3-((4-Hydroxyphenethyl)amino)-8-methoxy-9*H*-benzo[de][1,6]naphthyridin-9-one (17)

Through Procedure B, **C11** (86.5 mg, 0.17 mmol) was converted to **17** (23.0 mg, 40%) using BH_3·THF (1.7 mL, 1.7 mmol) and 6 M HCl (1.5 mL).

^{1}H-NMR (500 MHz, CD_3OD) δ: 8.65 (1H, d, J = 4.3 Hz), 8.10 (1H, s), 7.50 (1H, d, J = 4.3 Hz), 7.03 (1H, d, J = 9.0 Hz), 6.80 (1H, s), 6.61 (1H, d, J = 9.0 Hz), 3.85 (3H, s), 3.68 (2H, t, J = 7.2 Hz), 3.25 (1H, s), 2.88 (2H, t, J = 7.2 Hz). ^{13}C-NMR (125 MHz, DMSO-d_6) δ: 174.3, 157.3, 155.9, 150.6, 144.2, 135.6, 135.32, 135.29, 133.0, 129.7 (2C), 128.7, 122.2, 117.5, 115.2 (2C), 106.8, 55.8, 43.8, 33.8. IR (KBr): 2346, 1613, 1562, 1512, 1270 cm^{-1}. MS (ESI-TOF) *m/z*: 370 $[M+Na]^+$. HRMS (ESI-TOF) *m/z*: 370.1168, calcd for $C_{20}H_{17}N_3O_3Na$; Found: 370.1167.

8-Methoxy-3-((4-(prop-2-yn-1-yloxy)phenethyl)amino)-9*H*-benzo[*de*][1,6]naphthyridin-9-one (7)

K_2CO_3 (24.1 mg, 0.174 mmol, 5.0 equiv) and propargyl bromide (5.2 µL, 0.070 mmol, 2.0 equiv) were added to a solution of **17** (12.1 mg, 0.0348 mmol) in anhydrous DMF (0.4 mL), and the whole mixture was stirred for 12 h at rt. H_2O was added to the mixture, and the whole mixture was extracted with $CHCl_3$-MeOH (10:1). Removal of the solvent from the $CHCl_3$ extract under reduced pressure gave a crude product, which was purified by SiO_2 column ($CHCl_3$/MeOH = 30:1 + 1% Et_3N) and ODS column chromatography (70% MeOH) to give **7** (11.7 mg, 87%) as a red solid.

^{1}H-NMR (500 MHz, $CDCl_3$) δ: 8.74 (1H, d, J = 4.6 Hz), 8.39 (1H, s), 7.45 (1H, d, J = 4.6 Hz), 7.21 (2H, d, J = 8.6 Hz), 6.99 (1H, t, J = 5.7 Hz), 6.96 (2H, d, J = 8.6 Hz), 6.61 (1H, s), 4.69 (2H, d, J = 2.9 Hz), 3.97 (3H, s), 3.77 (2H, q, J = 7.0 Hz), 3.06 (2H, t, J = 7.0 Hz), 2.52 (1H, t, J = 2.3 Hz). ^{13}C-NMR (125 MHz, $CDCl_3$) δ: 175.9, 157.7, 156.5, 150.6, 143.8, 136.2, 134.62, 134.55, 130.8, 129.7 (2C), 129.5, 121.6, 117.8, 115.3, 106.2, 78.5, 75.6, 56.0, 55.8, 44.2, 34.5. IR (KBr): 1615, 1563, 1542, 1519, 1272 cm^{-1}. MS (ESI-TOF) *m/z*: 408 $[M+Na]^+$. HRMS (ESI-TOF) *m/z*: 408.1324, calcd for $C_{23}H_{19}N_3O_3Na$; Found: 408.1339.

***N*-(32-(4-((4-(2-((8-Methoxy-9-oxo-9*H*-benzo[*de*][1,6]naphthyridin-3-yl)amino)ethyl)phenoxy)methyl)-1*H*-1,2,3-triazol-1-yl)-3,6,9,12,15,18,21,24,27,30-decaoxadotriacontyl)-5-((3a*S*,4*S*,6a*R*)-2-oxohexahydro-1*H*-thieno[3,4-*d*]imidazol-4-yl)pentanamide (12)**

$CuSO_4$ (0.05 M solution in H_2O, 24.0 µL, 0.2 equiv.) and sodium ascorbate (0.05 M solution in H_2O, 48.0 µL, 0.4 equiv.) were added to a solution of alkyne **7** (2.3 mg, 5.97 µmol) and **18** (4.9 mg, 6.56 µmol, 1.1 equiv.) in *tert*-BuOH (0.3 mL), and the whole mixture was stirred at rt for 16 h. Removal of solvent from the mixture under reduced pressure gave the crude product, which was purified by open column chromatography eluted with $CHCl_3$/MeOH/H_2O (30:3:1, lower phase) and ODS column chromatography (70% MeOH) to give **12** (3.7 mg, 54%) as a red solid.

^{1}H-NMR (500 MHz, $CDCl_3$) δ: 8.75 (1H, d, J = 4.6 Hz), 8.39 (1H, s), 7.85 (1H, s), 7.47 (1H, d, J = 4.6 Hz),

7.19 (2H, d, J = 8.6 Hz), 7.03 (1H, t, J = 6.2 Hz), 6.97 (2H, d, J = 8.6 Hz), 6.62 (1H, s), 5.59 (1H, s), 5.19 (2H, s), 4.87 (1H, s), 4.56 (2H, t, J = 5.0 Hz), 4.50 (1H, t, J = 6.4 Hz), 4.32 (1H, t, J = 6.0 Hz), 3.97 (3H, s), 3.88 (2H, t, J = 5.0 Hz), 3.77 (2H, q, J = 6.8 Hz), 3.64-3.60 (40H, m), 3.56 (3H, t, J = 5.0 Hz), 3.43 (2H, dd, J = 10.2, 5.3 Hz), 3.15 (1H, td, J = 7.2, 4.7 Hz), 3.05 (2H, t, J = 7.2 Hz), 2.91 (1H, dd, J = 12.9, 4.9 Hz), 2.72 (1H, d, J = 12.6 Hz), 2.22 (2H, td, J = 7.3, 2.9 Hz), 1.45 (2H, q, J = 7.4 Hz). IR (KBr): 3367, 2916, 2870, 1695, 1640, 1564, 1511, 1272 cm^{-1}. MS (ESI-TOF) *m/z*: 1160 $[M+Na]^+$. HRMS (ESI-TOF) *m/z*: 1160.5314, calcd for $C_{55}H_{79}N_9O_{15}NaS$; Found: 1160.5356.

_N_-(8-(4-((4-(2-((8-methoxy-9-oxo-9_H_-benzo[_de_][1,6]naphthyridin-3-yl)amino)ethyl)phenoxy)methyl)-1_H_-1,2,3-triazol-1-yl)octyl)-5-(2-oxohexahydro-1_H_-thieno[3,4-_d_]imidazol-4-yl)pentanamide (13)

Through the same procedure as the synthesis of **12**, alkyne **7** (3.1 mg, 8.04 µmol) and **19** (3.5 mg, 8.85 µmol) was converted to **13** (2.1 mg, 33%) as a red solid.

^{1}H-NMR (500 MHz, $CDCl_3$) δ: 8.75 (1H, d, J = 4.6 Hz), 8.35 (1H, s), 7.61 (1H, s), 7.47 (1H, d, J = 4.6 Hz), 7.19 (2H, d, J = 8.6 Hz), 7.03 (1H, t, J = 6.2 Hz), 6.95 (2H, d, J = 8.6 Hz), 6.62 (1H, s), 5.72 (1H, brs), 5.35 (1H, brs), 5.20 (2H, s), 4.83 (1H, brs), 4.51 (2H, t, J = 5.0 Hz), 4.35 (1H, t, J = 6.4 Hz), 4.33 (1H, t, J = 6.0 Hz), 3.97 (3H, s), 3.77 (2H, t, J = 5.0 Hz), 3.28–3.12 (3H, m), 3.05 (2H, t, J = 7.2 Hz), 2.92 (1H, dd, J = 12.9, 4.9 Hz), 2.72 (1H, d, J = 12.6 Hz), 2.18 (2H, td, J = 7.3, 2.9 Hz), 1.89 (2H, t, J = 3.4 Hz), 1.75–1.20 (16H, m). MS (ESI-TOF) *m/z*: 805 $[M+Na]^+$. HRMS (ESI-TOF) *m/z*: 804.3626, calcd for $C_{41}H_{51}N_9O_5NaS$; Found: 804.3650.

8-Hydroxy-3-(phenethylamino)-9_H_-benzo[_de_][1,6]naphthyridin-9-one (8)

BBr_3 (1.0 M solution in CH_2Cl_2, 0.466 mL, 0.466 mmol, 6.0 equiv.) was added to a solution of **1** (25.7 mg, 0.078 mmol) in anhydrous CH_2Cl_2 (5.0 mL), and the whole mixture was stirred at rt for 7 h. MeOH (2 drops) and sat. $NaHCO_3$ aq. were added to the mixture, and the whole mixture was extracted with $CHCl_3$-MeOH (10:1). Removal of the solvent from the $CHCl_3$ extract under reduced pressure gave a crude product **8**, which was used next reaction without further purification.

A small amount was purified by SiO_2 column ($CHCl_3$/MeOH/H_2O = 40:3:1 containing 1% Et_3N, lower phase) and reverse phase column chromatography (70% MeOH) to give **8** as a red solid.

^{1}H-NMR (500 MHz, CD_3OD) δ: 8.74 (1H, d, J = 4.6 Hz), 8.42 (1H, s), 7.48 (1H, d, J = 4.6 Hz), 7.36-7.35 (2H, m), 7.29-7.28 (3H, m), 6.85 (1H, s), 3.84 (2H, q, J = 7.1 Hz), 3.13 (2H, t, J = 7.1 Hz). ^{13}C-NMR (125 MHz, DMSO-d_6) δ: 176.1, 154.1, 150.7, 144.8, 137.6, 136.5, 136.0, 132.8, 130.1, 128.9 (2C), 128.7 (2C), 127.0, 122.4, 118.3, 107.1, 44.2, 35.4. IR (KBr): 3285, 1617, 1561, 1257, 1168 cm^{-1}. MS (ESI-TOF) *m/z*: 340 $[M+Na]^+$. HRMS (ESI-TOF) *m/z*: 340.1062, calcd for $C_{19}H_{15}N_3O_2Na$; Found: 340.1066.

3-(Phenethylamino)-8-(prop-2-yn-1-yloxy)-9_H_-benzo[_de_][1,6]naphthyridin-9-one (9)

K_2CO_3 (32.2 mg, 0.233 mmol, 3.0 equiv) and propargyl bromide (6.4 µL, 0.085 mmol, 1.1 equiv) were added to a solution of crude **8** in anhydrous DMF (5.0 mL), and the whole mixture was stirred for 8 h at rt. H_2O was added to the mixture and the whole mixture was extracted with $CHCl_3$. Removal of the solvent from the $CHCl_3$ extract under reduced pressure gave a crude product, which was purified by SiO_2 column

($CHCl_3$/MeOH = 30:1 containing 1% Et_3N, lower phase) and ODS column chromatography (70% MeOH) to give **9** (9.5 mg, 34%) as a red solid.

^{1}H-NMR (500 MHz, $CDCl_3$) δ: 8.76 (1H, d, *J* = 4.6 Hz), 8.40 (1H, s), 7.50 (1H, d, *J* = 4.6 Hz), 7.36-7.34 (2H, m), 7.29-7.27 (3H, m), 7.02 (1H, t-like, *J* = 6.4 Hz), 6.83 (1H, s), 4.89 (2H, d, *J* = 2.3 Hz), 3.81 (2H, q, *J* = 7.1 Hz), 3.11 (2H, t, *J* = 7.1 Hz), 2.60 (1H, t, *J* = 2.3 Hz). ^{13}C-NMR (125 MHz, $CDCl_3$) δ: 175.7, 155.4, 150.7, 143.8, 137.8, 136.2, 135.8, 134.6, 129.6, 128.9 (2C), 128.7 (2C), 127.0, 122.1, 117.9, 108.7, 77.3, 76.7, 56.3, 44.2, 35.4. IR (KBr): 3361, 1639, 1617, 1562, 1543, 1269, 1194 cm^{-1}. MS (ESI-TOF) *m/z*: 378 [M + Na]$^+$. HRMS (ESI-TOF) *m/z*: 378.1218, calcd for $C_{22}H_{17}N_3O_2Na$; Found: 378.1236.

***N*-(32-(4-(((9-oxo-3-(phenethylamino)-9*H*-benzo[de][*1,6*]naphthyridin-8-yl)oxy)methyl)-1*H*-1,2,3-triazol-1-yl)-3,6,9,12,15,18,21,24,27,30-decaoxadotriacontyl)-5-((3a*S*,4*S*,6a*R*)-2-oxohexahydro-1*H*-thieno[3,4-*d*]imidazol-4-yl)pentanamide (14)**

$CuSO_4$ (0.05 M solution in H_2O, 40.0 μL, 0.2 equiv.) and sodium ascorbate (0.05 M solution in H_2O, 79.0 μL, 0.4 equiv.) were added to a solution of alkyne **9** (3.5 mg, 9.85 μmol) and **18** (7.8 mg, 10.3 μmol, 1.05 equiv.) in *tert*-BuOH (0.2 mL) and DMF (0.5 mL), and the whole mixture was stirred at rt for 16 h. $CuSO_4$ (0.05 M solution in water, 40.0 μL, 0.2 equiv) and sodium ascorbate (0.8 mg, 0.4 equiv) were added to the mixture, and the whole mixture was stirred for 6h. Then, the reaction mixture was evaporated. The resulting mixture was purified by open column chromatography eluted with $CHCl_3$/MeOH/H_2O (30:3:1, lower phase) and reverse phase column chromatography (70% MeOH) to give **14** (4.7 mg, 43%) as a red solid.

^{1}H-NMR (500 MHz, $CDCl_3$) δ: 8.73 (1H, d, *J* = 4.5 Hz), 8.39 (1H, s), 7.99 (1H, s), 7.51 (1H, d, *J* = 4.5 Hz), 7.35-7.33 (2H, m), 7.28-7.27 (3H, m), 7.06 (1H, s), 7.02 (1H, t-like, *J* = 5.9 Hz), 6.64 (1H, br s), 5.85 (1H, br s), 5.41 (2H, s), 5.14 (1H, br s), 4.55 (2H, t, *J* = 5.0 Hz), 4.51 (1H, t, *J* = 6.7 Hz), 4.32 (1H, t, *J* = 6.7 Hz), 3.87 (2H, t, *J* = 4.9 Hz), 3.79 (2H, q, *J* = 7.0 Hz), 3.64-3.55 (38H, m), 3.44-3.42 (2H, m), 3.15-3.14 (1H, m), 3.10 (2H, t, *J* = 7.0 Hz), 2.90 (1H, dd, *J* = 12.7, 4.7 Hz), 2.74 (1H, d, *J* = 12.6 Hz), 2.23-2.20 (2H, m), 1.83 (1H, br s), 1.76-1.63 (3H, m), 1.46-1.40 (2H, m). IR (KBr): 3361, 2917, 2871, 1696, 1642, 1564, 1460, 1270, 1111 cm^{-1}. MS (ESI-TOF) *m/z*: 1130 [M+Na]$^+$. HRMS (ESI-TOF) *m/z*: 1130.5208, calcd for $C_{54}H_{77}N_9O_{14}NaS$; Found: 1130.5222.

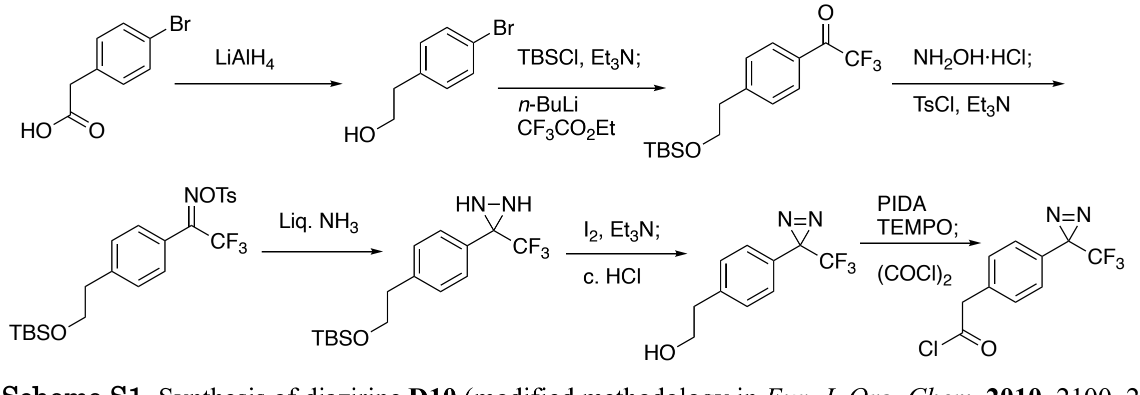

Scheme S1. Synthesis of diazirine **D10** (modified methodology in *Eur. J. Org. Chem.* **2010**, 2100–2112.)

2-(4-Bromophenyl)ethanol

$LiAlH_4$ (2.36 g, 63.7 mmol, 1.3 equiv) was carefully added to a solution of 2-(4-bromophenyl)acetic acid (10.5 g, 49.0 mmol) in E_2O (250 mL) at 0 °C, and the whole mixture was stirred at rt for 1h. 1 M HCl was slowly added to the solution at 0 °C, and the whole mixture was extracted with Et_2O. Combined organic layer was washed with 1 M HCl, sat. $NaHCO_3$ aq., H_2O, and brine. Removal of the solvent from the reaction mixture under reduced pressure gave a crude product (9.80 g, 99%) as colorless oil, which was used next reaction withput further purification.

(4-Bromophenethoxy)(*tert*-butyl)dimethylsilane

TBSCl (7.71 g, 51.2 mmol, 1.05 equiv.) and DMAP (1.19 g, 9.75 mmol, 0.2 equiv.) were added to a solution of alcohol (9.80 g, 48.7 mmol) and imidazole (6.64 g, 97.5 mmol, 2.0 equiv.) in CH_2Cl_2 (80 mL) at rt, and the whole mixture was stirred at rt for 2 min. Sat. NH_4Cl aq. was added to the mixture, and the whole mixture was extracted with CH_2Cl_2. Combined organic layer was washed with deionised H_2O and brine. Removal of the solvent from the reaction mixture under reduced pressure gave a crude product (15.4 g, quant.) as colorless oil, which was used next reaction withput further purification.

1-(4-(2-((*tert*-Butyldimethylsilyl)oxy)ethyl)phenyl)-2,2,2-trifluoroethanone

n-BuLi (15.3 mL, of a 1.60 M solution in hexanes, 24.5 mmol, 1.1 equiv) was slowly added dropwise to a solution of bromide (7.13 g, 22.3 mmol) in THF (80 mL) at –78 °C, and the whole mixture was stirred at –78 to –40 °C for 40 min. A solution of ethyl trifluoroacetate (2.80 mL, 23.4 mmol, 1.05 equiv) in THF (5.0 mL) was added to the mixture via *cannula* at –78 °C, and the whole mixture was stirred at –78 °C for 40 min. Sat. NH_4Cl aq. was added to the mixture, and the whole mixture was extracted with Et_2O. Removal of the solvent from Et_2O extract under reduced pressure gave a crude product (7.12 g, 96%) as colorless oil, which was used next reaction withput further purification.

1-(4-(2-((*tert*-Butyldimethylsilyl)oxy)ethyl)phenyl)-2,2,2-trifluoroethanone *O*-tosyl oxime

$NH_2OH \cdot HCl$ (4.47 g, 64.3 mmol, 3.0 equiv) was added dropwise to a solution of ketone (7.12 g, 21.4 mmol) in pyridine (25 mL), and the whole mixture was stirred with reflux (140 °C) for 2 h. The mixture was concentrated, and the resulting mixture was dissolved in EtOAc and 5% HCl. The whole mixture was extracted with EtOAc, and combined organic layer was washed with 5% HCl, deionised H_2O, and brine. Removal of the solvent from the reaction mixture under reduced pressure gave a crude product, which was dissolved in in CH_2Cl_2 (20 mL). Et_3N (4.48 mL, 32.1 mmol, 1.5 equiv) and DMAP (262 mg, 2.14 mmol, 0.1 equiv) were added to the mixture, and TsCl (4.08 g, 21.4 mmol, 1.0 equiv) in CH_2Cl_2 (20 mL) was added dropwise to the mixture for 1 h. The whole mixture was stirred at rt for 30 min. Sat. NH_4Cl aq. was added to the mixture, and the whole mixture was extracted with CH_2Cl_2. The combined organic layer was washed with 1 M HCl and brine. Removal of the solvent from Et_2O extract under reduced pressure gave a crude product, which was purified by SiO_2 column (*n*-Hexanes/EtOAc = 8:1 to 4:1) to give tosylate (9.54 g, 89%, *Z/E* mixture) as colorless oil.

^{1}H-NMR (500 MHz, $CDCl_3$) δ: 7.88-7.87 (2H, m), 7.38-7.24 (6H, m), 3.83-3.78 (2H, m), 2.86-2.82 (2H, m), 2.46 (1.5H, s), 2.44 (1.5H, s), 0.83 (9H, s), –0.05 (6H, s). ^{13}C-NMR (125 MHz, $CDCl_3$) δ: 154.03 (C=N, 0.5 C, q, J = 32.9 Hz), 153.96 (C=N, 0.5C, 153.96, J = 32.1 Hz), 146.0 (0.5C), 145.9 (0.5C), 143.9 (0.5C), 143.7 (0.5C), 131.5 (0.5C), 131.3 (0.5C), 129.8 (2C), 129.64, 129.56, 129.2, 129.1, 128.7, 128.3, 125.5 (0.5C), 122.3 (0.5C), 119.7 (CF_3, 1C, q, J = 276 Hz), 63.8 (0.5C), 63.7 (0.5C), 39.4 (0.5C), 39.3 (0.5C), 25.8 (3C), 21.8 (0.5C), 21.7 (0.5C), 18.3, –5.5 (2C). IR (KBr): 2954, 2930, 2858, 1598, 1471, 1391, 1181, 1151, 1095 cm^{-1}. MS (ESI-TOF) *m/z*: 524 $[M+Na]^+$. HRMS (ESI-TOF) *m/z*: 524.1515 calcd for $C_{23}H_{30}F_3NO_4SSiNa$; Found: 524.1525.

3-(4-(2-((*tert*-Butyldimethylsilyl)oxy)ethyl)phenyl)-3-(trifluoromethyl)diaziridine

Liq. NH_3 (~ 3 mL) was added to a solution of tosylate (6.41 g, 12.8 mmol) in THF (15 mL) at –78 °C, and the whole mixture was stirred at –78 °C for 6 h. Then, the mixture was carefully warmed to rt with exhausting NH_3 gas. H_2O was added to the mixture, and the whole mixture was extracted with Et_2O. The combined organic layer was washed with H_2O and brine. Removal of the solvent from the Et_2O extract under reduced pressure gave a crude product (4.45 g, quant.) as colorless oil, which was used next reaction without further purification.

^{1}H-NMR (500 MHz, $CDCl_3$) δ: 7.52 (2H, d, J = 8.0 Hz), 7.27 (2H, d, J = 8.0 Hz), 3.80 (2H, t, J = 6.9 Hz), 2.84 (2H, t, J = 6.9 Hz), 2.77 (1H, d, J = 8.9 Hz), 2.18 (1H, d, J = 8.9 Hz), 0.85 (9H, s), -0.03 (6H, s). ^{13}C-NMR (125 MHz, $CDCl_3$) δ: 141.7, 129.54 (2C), 129.46, 127.9 (2C), 123.6 (CF_3, 1C, q, J = 278 Hz), 64.0, 57.9 (CNHNH, 1C, q, J = 35.8 Hz), 39.2, 25.8 (3C), 18.3, –5.5 (2C). IR (KBr): 3228, 2954, 2930, 2859, 1256, 1216, 1153, 1097 cm^{-1}. MS (ESI-TOF) *m/z*: 369 $[M+Na]^+$. HRMS (ESI-TOF) *m/z*: 369.1586, calcd for $C_{16}H_{25}F_3N_2OSiNa$; Found: 369.1576.

3-(4-(2-((*tert*-Butyldimethylsilyl)oxy)ethyl)phenyl)-3-(trifluoromethyl)-3*H*-diazirine

Iodine (3.18 g, 12.5 mmol, 1.0 equiv.) was added portionwise to a solution of diaziridine (4.34 g, 12.5 mmol) and Et_3N (3.49 mL, 25.1 mmol, 2.0 equiv.) in MeOH (13 mL) at 0 °C, and the whole mixture was stirred at rt for 1.5 h. 1 M HCl and sat. $Na_2S_2O_5$ aq. were added to the mixture, and the whole mixture was extracted with Et_2O. The combined organic layer was washed with 1N HCl, sat. $Na_2S_2O_5$ aq., H_2O, and brine. Removal of the solvent from the Et_2O extract under reduced pressure gave a crude product (4.28 g, 99%) as colorless oil, which was used next reaction without further purification

^{1}H-NMR (500 MHz, $CDCl_3$) δ: 7.24 (2H, d, J = 8.0 Hz), 7.11 (2H, d, J = 8.0 Hz), 3.78 (2H, t, J = 6.8 Hz), 2.82 (2H, t, J = 6.8 Hz), 0.85 (9H, s), -0.04 (5H, s). ^{13}C-NMR (125 MHz, $CDCl_3$) δ: 141.3, 129.7 (2C), 126.9, 126.3 (2C), 122.21 (CF_3, 1C, q, J = 275 Hz), 63.9, 39.1, 28.4 (CNN, 1C, q, J = 40.4 Hz), 25.8 (3C), 18.3, -5.5 (2C). IR (KBr): 2954, 2931, 2859, 1346, 1238, 1185, 1157, 1101 cm^{-1}. MS (ESI-TOF) *m/z*: 367 $[M+Na]^+$. HRMS (ESI-TOF) *m/z*: 367.1429, calcd for $C_{16}H_{23}F_3N_2OSiNa$; Found: 367.1423.

2-(4-(3-(Trifluoromethyl)-3*H*-diazirin-3-yl)phenyl)ethanol

Conc. HCl (1.5 mL) was added to a solution of diazirine (4.18 g, 12.1 mmol) in MeOH (25 mL) at 0 °C, and the whole mixture was stirred at rt for 30 min. The mixture was concentrated, and the resulting mixture

was dissolved in Et_2O and H_2O. The whole mixture was extracted with Et_2O, and the combined organic layer was washed with sat. $NaHCO_3$ aq. and brine. Removal of the solvent from Et_2O extract under reduced pressure gave a crude product, which was purified by SiO_2 column (*n*-Hexanes/EtOAc = 4:1 to 2:1) to give **alcohol** (2.50 g, 89%) as colorless oil.

^{1}H-NMR (500 MHz, $CDCl_3$) δ: 7.27 (2H, d, *J* = 8.3 Hz), 7.15 (2H, d, *J* = 8.3 Hz), 3.86 (2H, q, *J* = 5.5 Hz), 2.88 (2H, t, *J* = 6.3 Hz), 1.37 (1H, t-like, *J* = 4.9 Hz). ^{13}C-NMR (125 MHz, $CDCl_3$) δ: 140.6, 129.5 (2C), 127.2, 126.6 (2C), 122.1 (CF_3, 1C, q, *J* = 275 Hz), 63.2, 38.7, 28.3 (CNN, 1C, q, *J* = 40.4 Hz). IR (KBr): 3317, 2949, 2880, 1616, 1521, 1346, 1232, 1153, 1053 cm^{-1}.

2-(4-(3-(Trifluoromethyl)-3*H*-diazirin-3-yl)phenyl)acetic acid

PIDA (8.36 g, 26.0 mmol, 2.5 equiv) and TEMPO (162 mg, 1.04 mmol, 0.1 equiv) were added to a solution of alcohol (2.39 g, 10.4 mmol) in CH_2Cl_2/H_2O (2:1, 45 mL) at 0 °C, and the whole mixture was stirred at rt for 4 h. Sat. $Na_2S_2O_4$ aq was added to the mixture, and the whole mixture was extracted with EtOAc. Removal of the solvent from EtOAc extract under reduced pressure gave a crude product, which was purified by SiO_2 column (*n*-Hexanes/EtOAc = 4:1 to 2:1) to give carboxylic acid (2.45 g, 97%) as a white solid.

^{1}H-NMR (500 MHz, $CDCl_3$) δ: 11.34 (1H, br s), 7.33 (2H, d, *J* = 8.2 Hz), 7.17 (2H, d, *J* = 8.2 Hz), 3.67 (2H, s). ^{13}C-NMR (125 MHz, $CDCl_3$) δ: 177.6, 134.8, 129.9 (2C), 128.3, 126.7 (2C), 122.1 (CF_3, 1C, q, *J* = 275 Hz), 40.6, 28.3 (CNN, 1C, q, *J* = 40.4 Hz). IR (KBr): 3023, 1712, 1413, 1348, 1233, 1182, 1151 cm^{-1}.

2-(4-(3-(Trifluoromethyl)-3*H*-diazirin-3-yl)phenyl)acetyl chloride (D10)

Oxalyl chloride (2.53 mL, of a 2.0 M solution in CH_2Cl_2, 5.06 mmol, 2.0 equiv) and DMF (1 drop) were added to a solution of carboxylic acid (619 mg, 2.53 mmol) in CH_2Cl_2 (13 mL) at 0 °C, and the whole mixture was stirred at rt for 3 h. The mixture was concentrated to give **D10**, which was used next reaction.

Figure S1. ^{1}H NMR spectrum of **2**

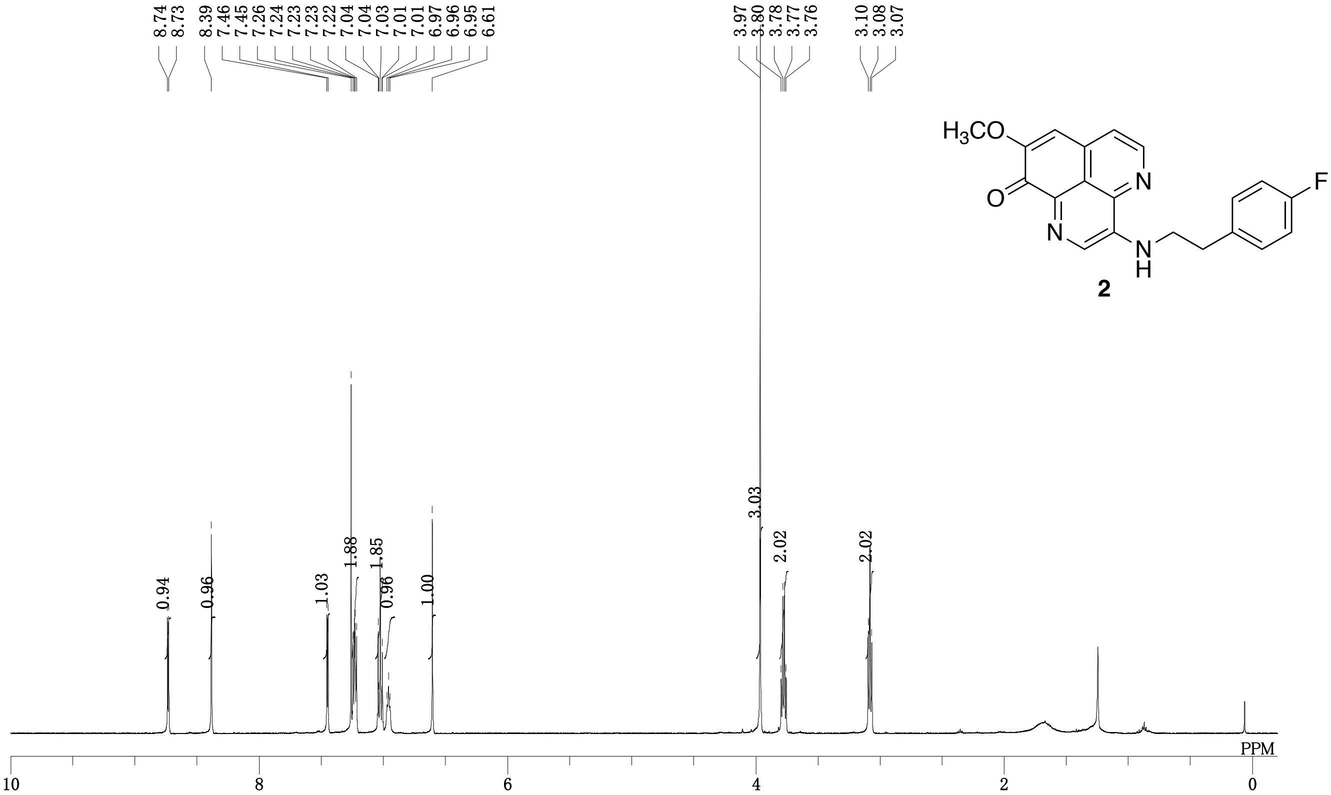

Figure S2. ^{13}C NMR spectrum of **2**

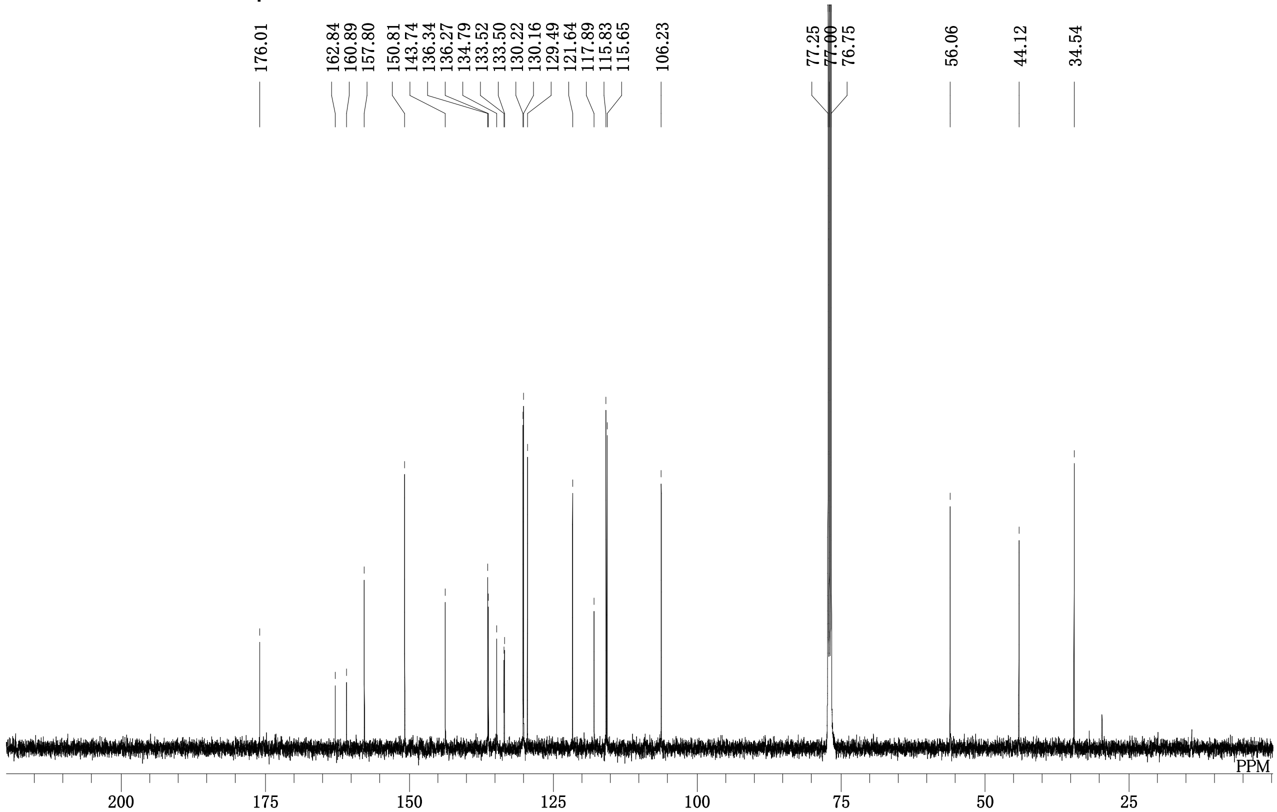

Figure S3. ^{1}H NMR spectrum of **3**

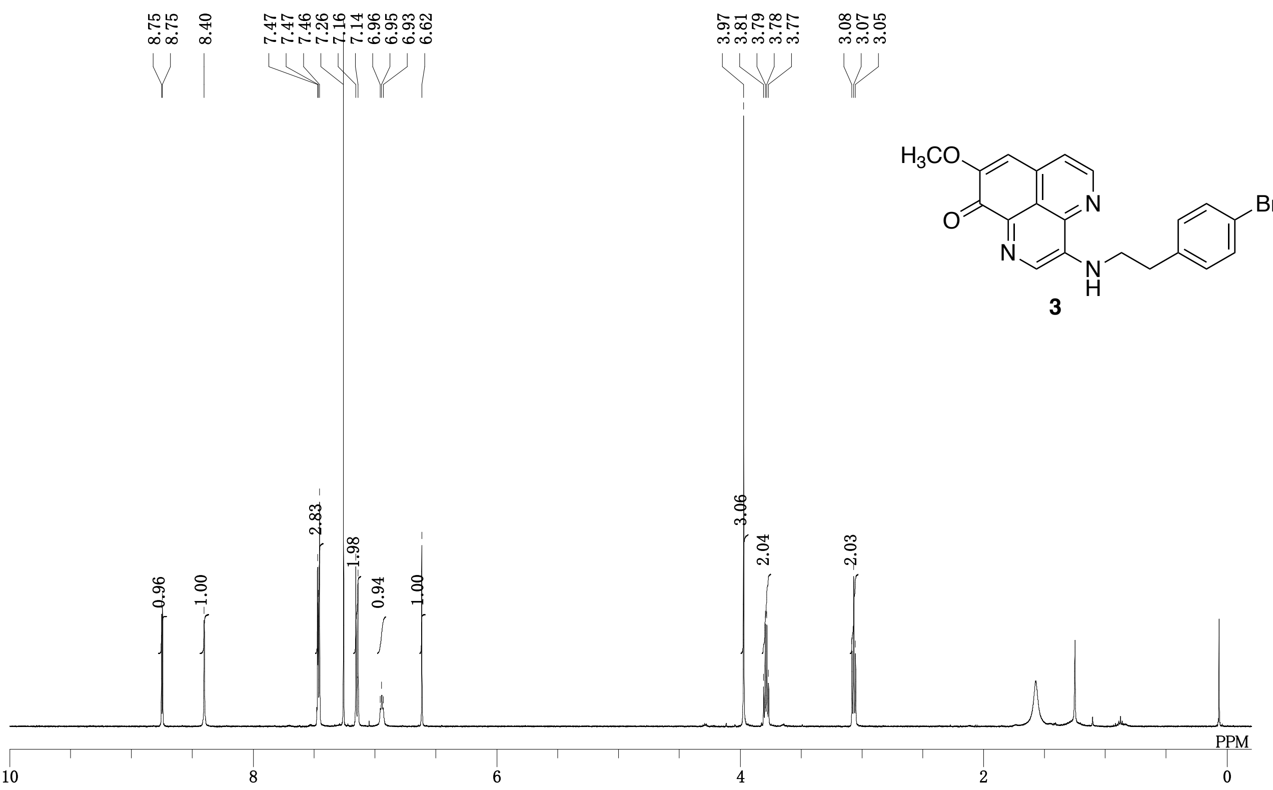

Figure S4. ^{13}C NMR spectrum of **3**

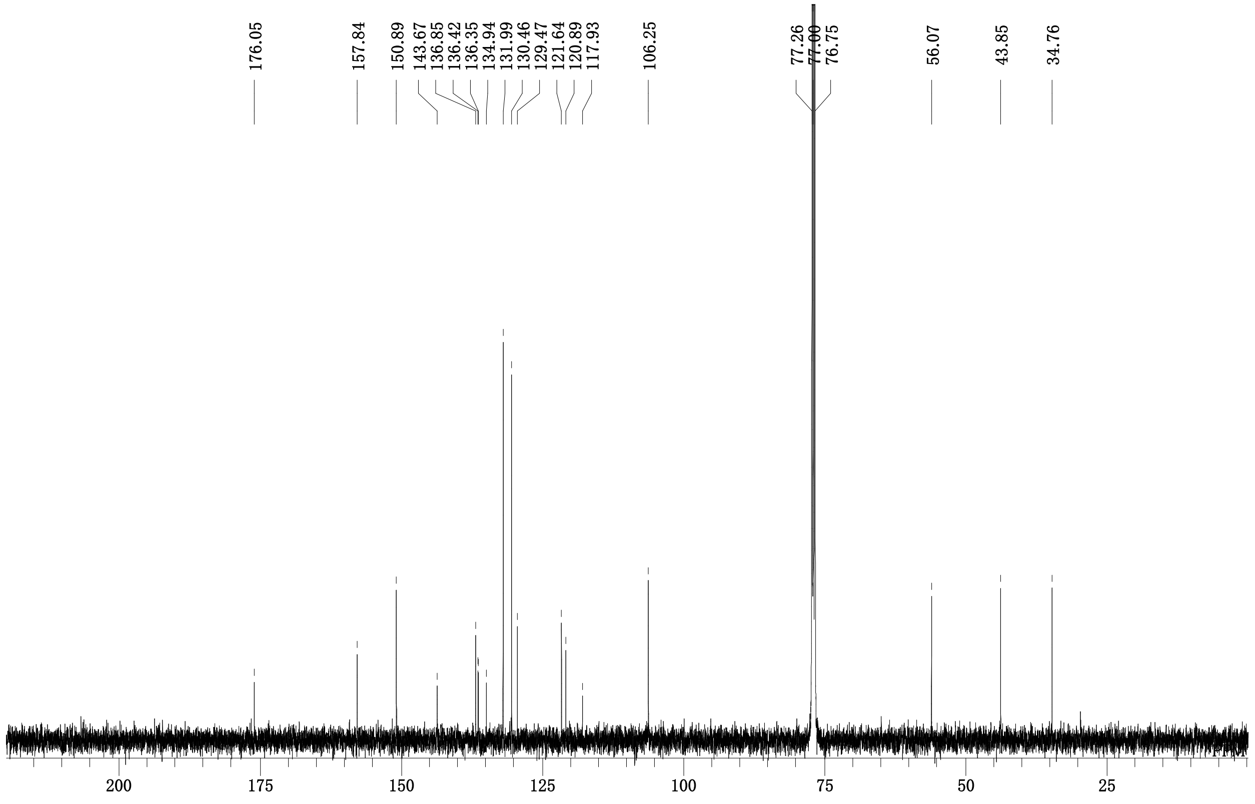

Figure S5. ^{1}H NMR spectrum of **4**

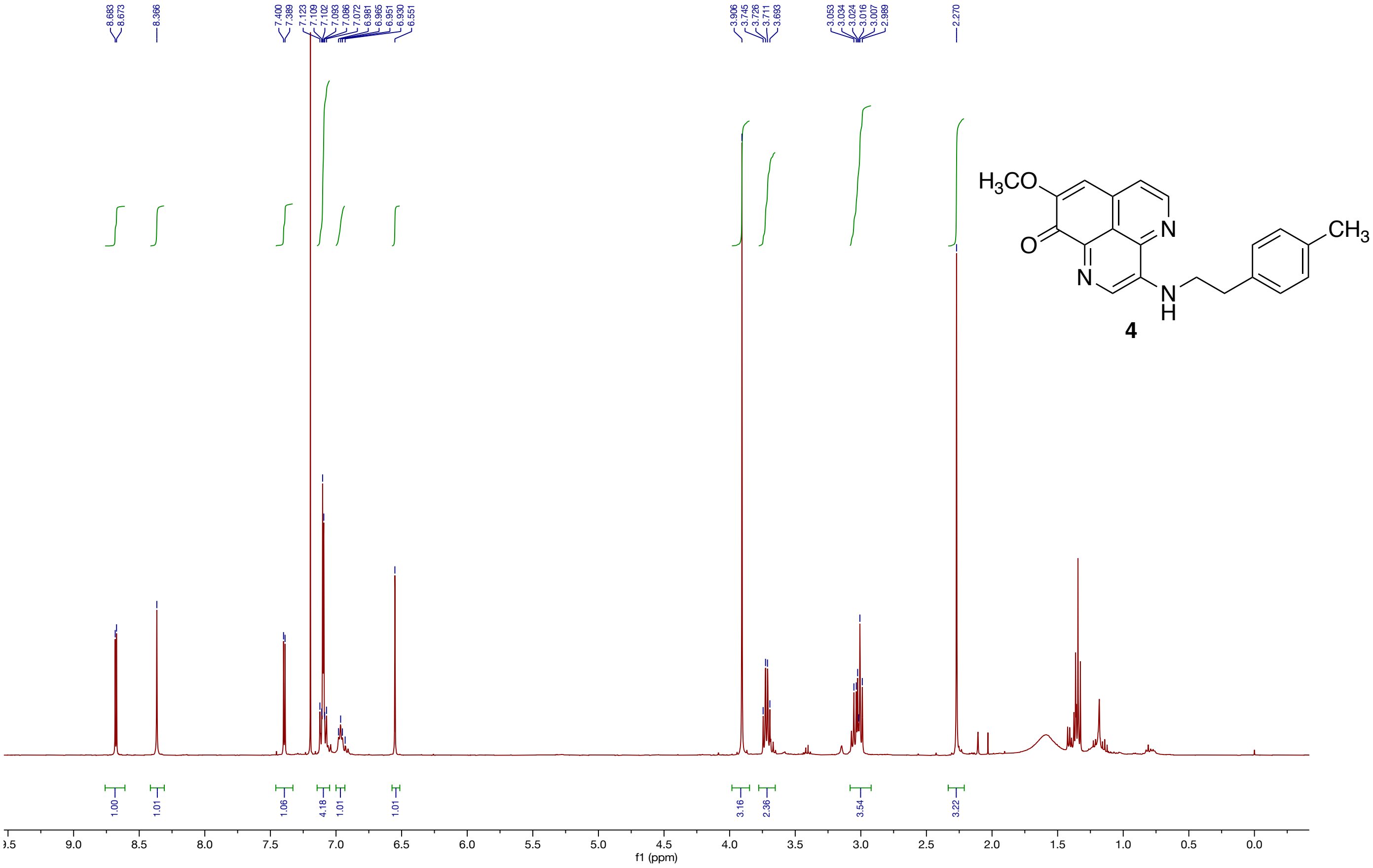

Figure S6. ^{13}C NMR spectrum of **4**

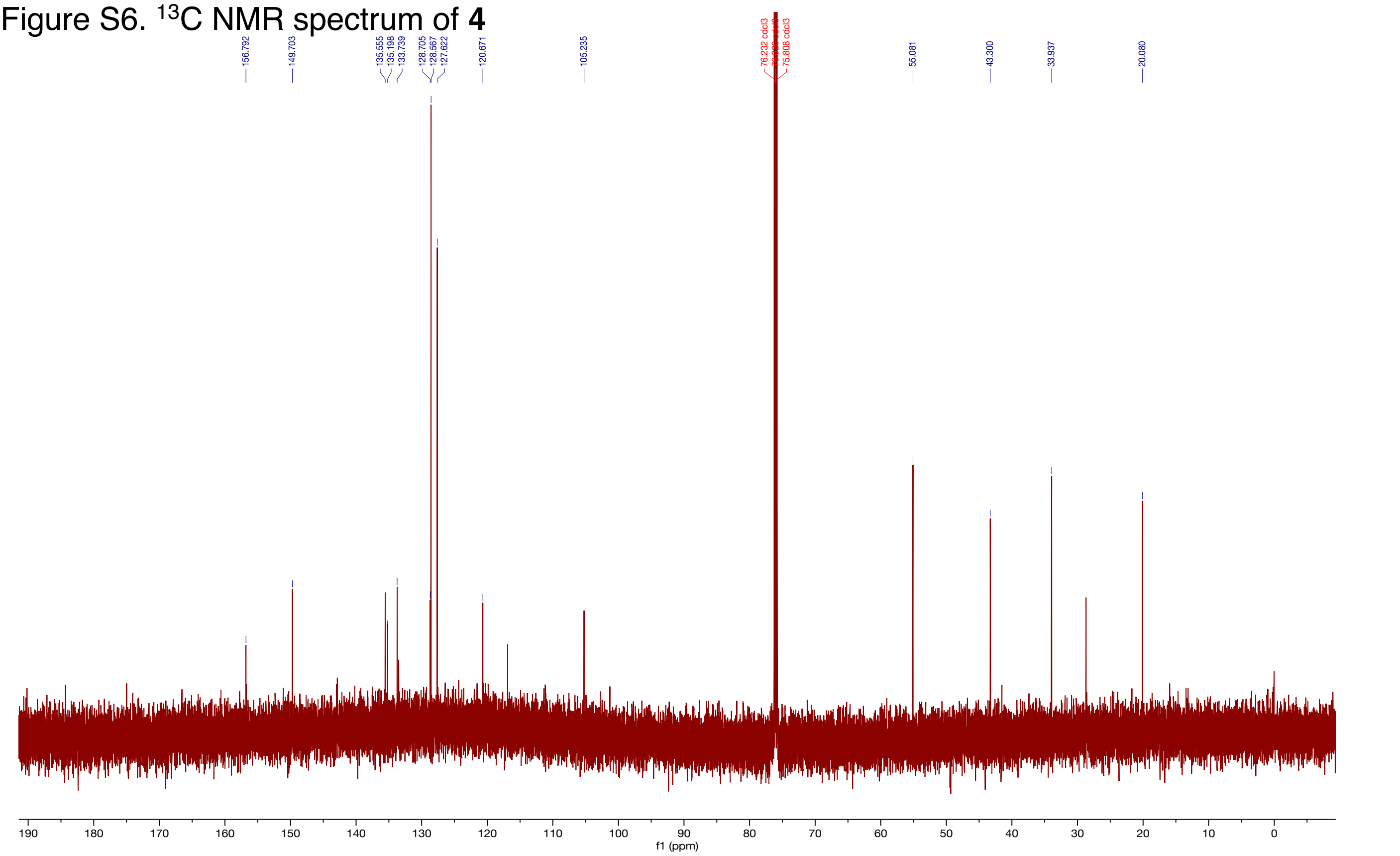

Figure S7. ^{1}H NMR spectrum of **5**

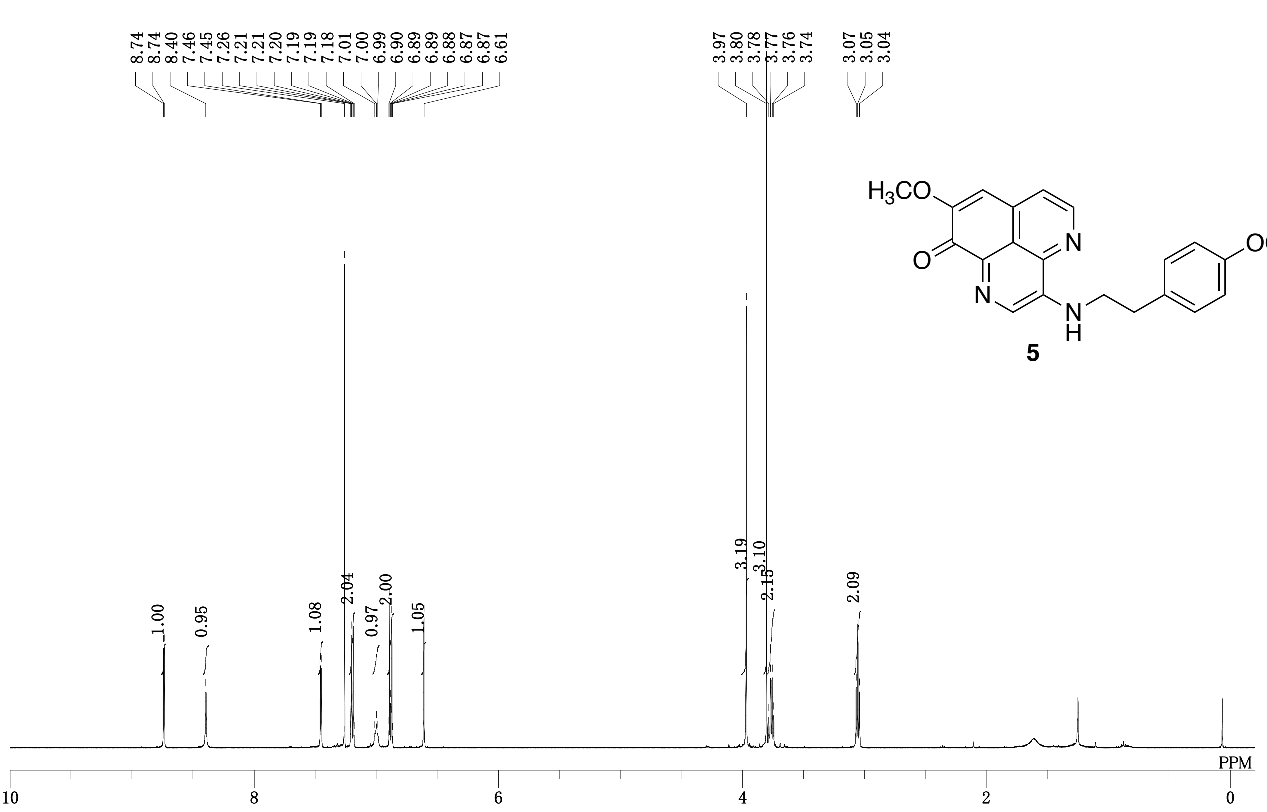

Figure S8. ^{13}C NMR spectrum of **5**

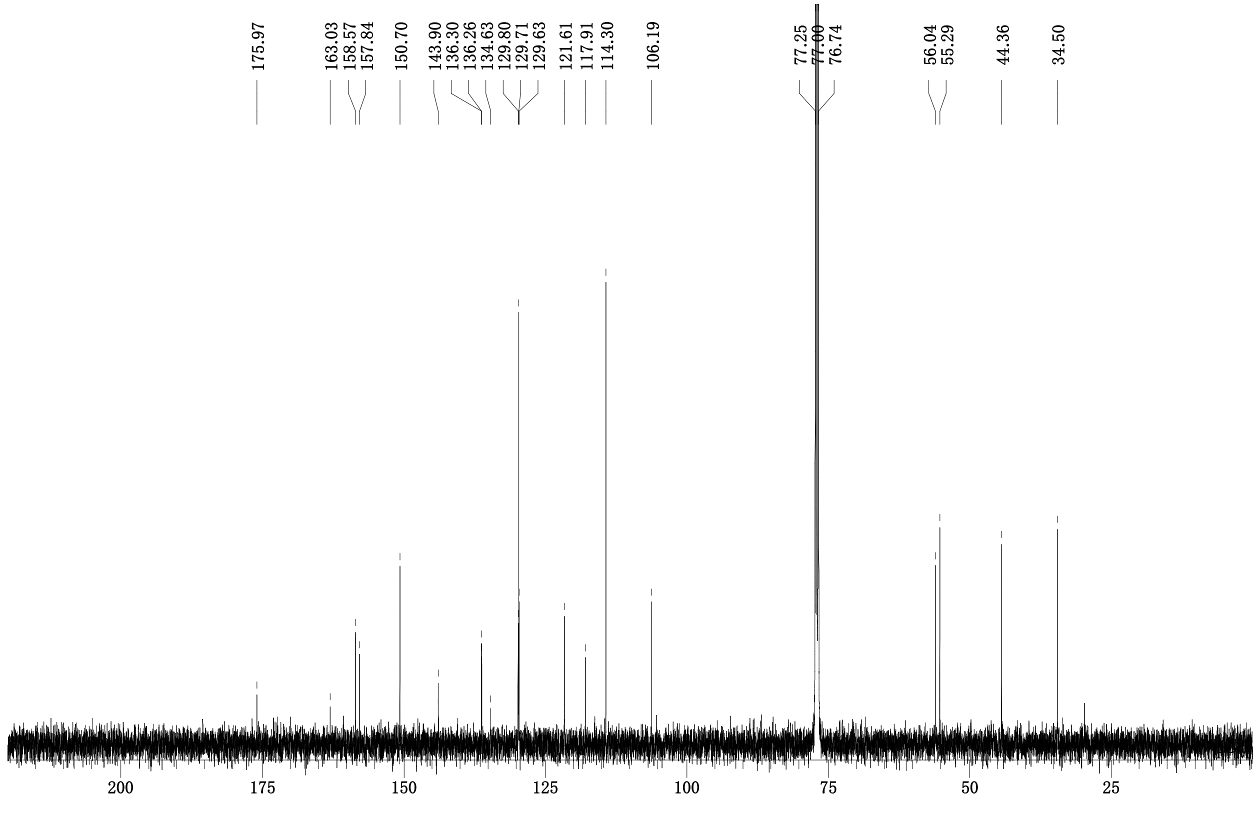

Figure S9. ^{1}H NMR spectrum of **6**

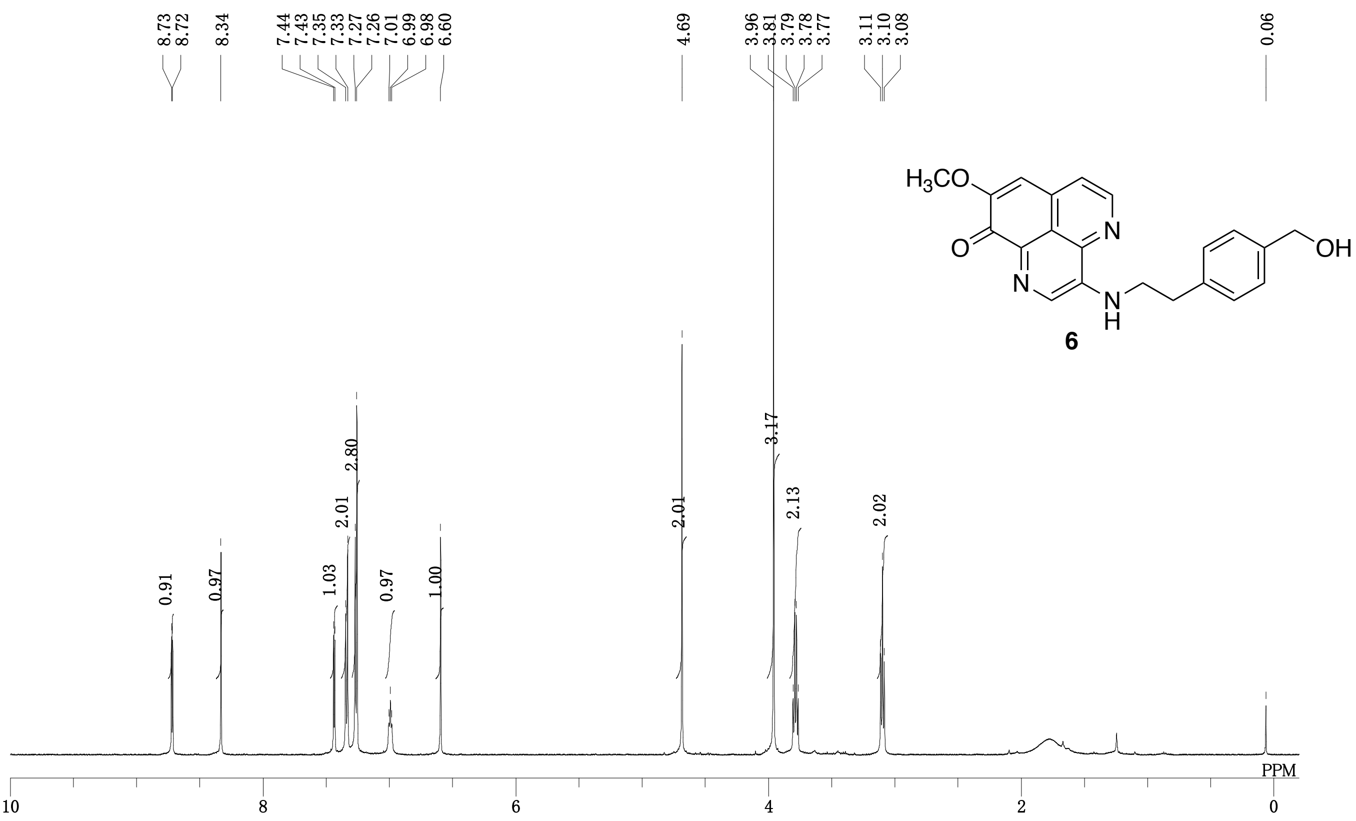

Figure S10. ^{13}C NMR spectrum of **6**

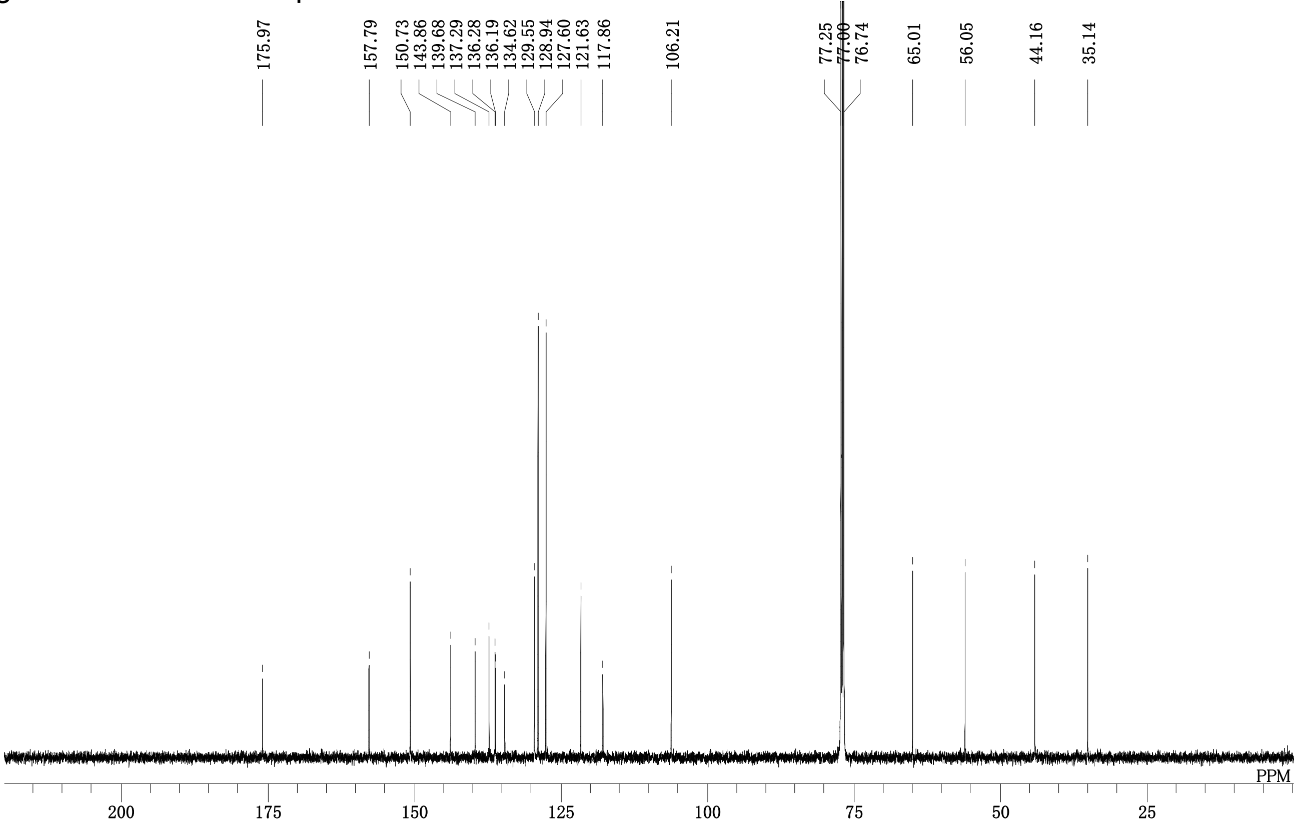

Figure S11. ^{1}H NMR spectrum of **10**

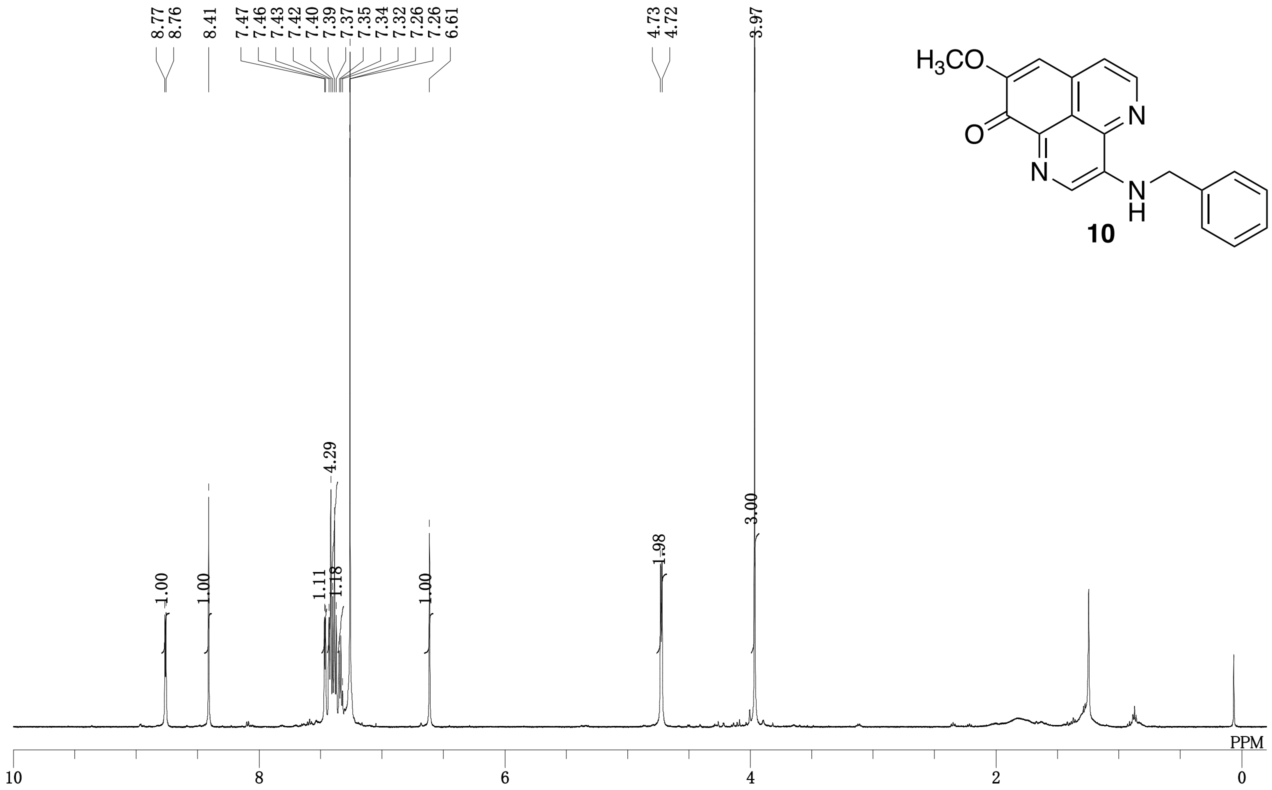

Figure S12. ^{13}C NMR spectrum of **10**

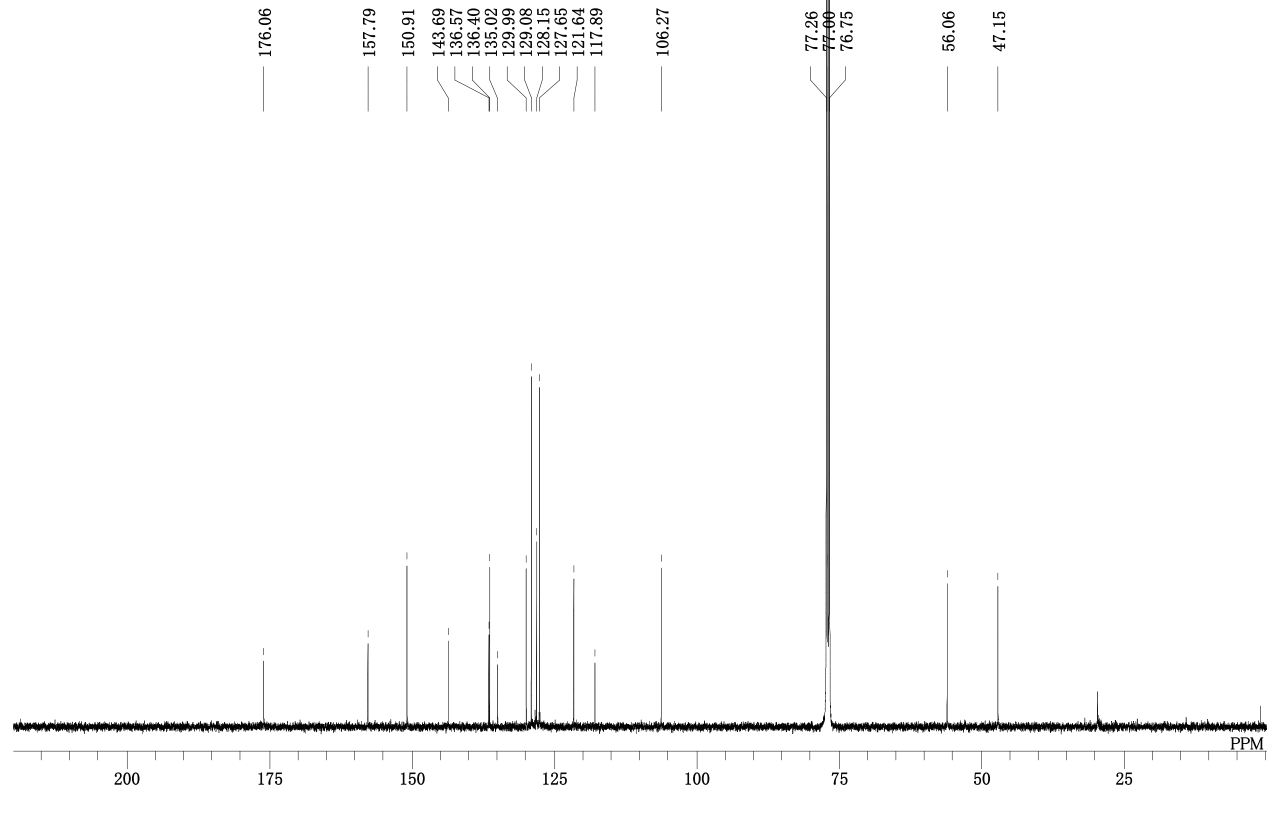

Figure S13. ^{1}H NMR spectrum of **11**

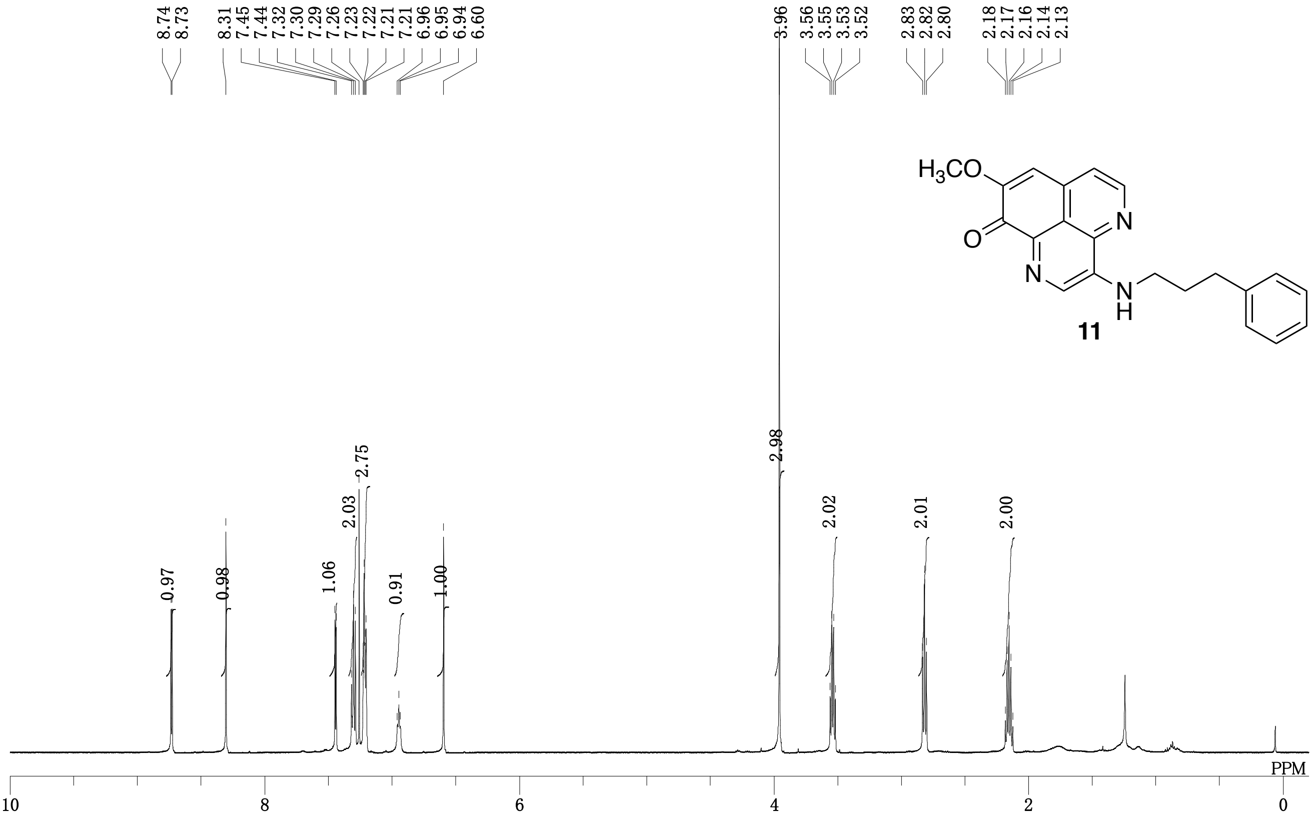

Figure S14. ^{13}C NMR spectrum of **11**

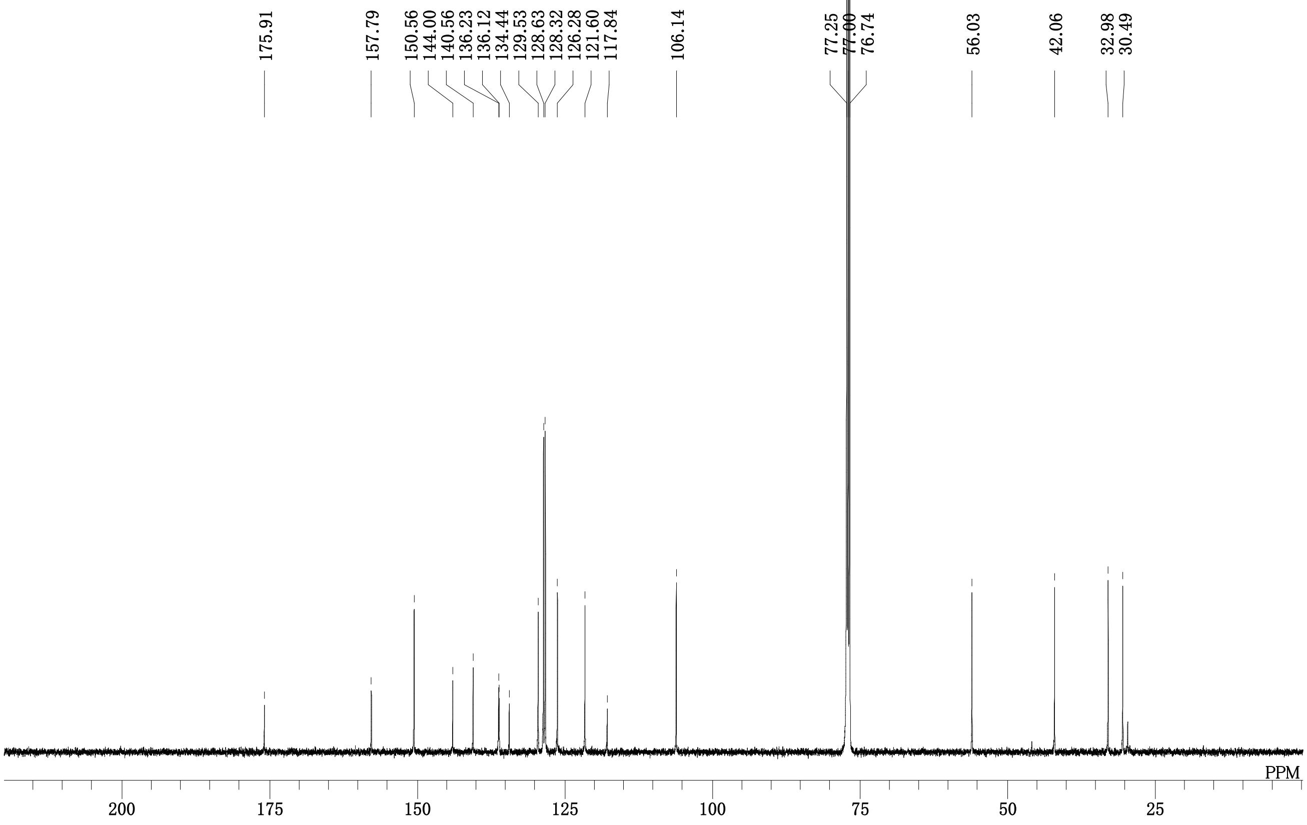

Figure S15. ^{1}H NMR spectrum of **15**

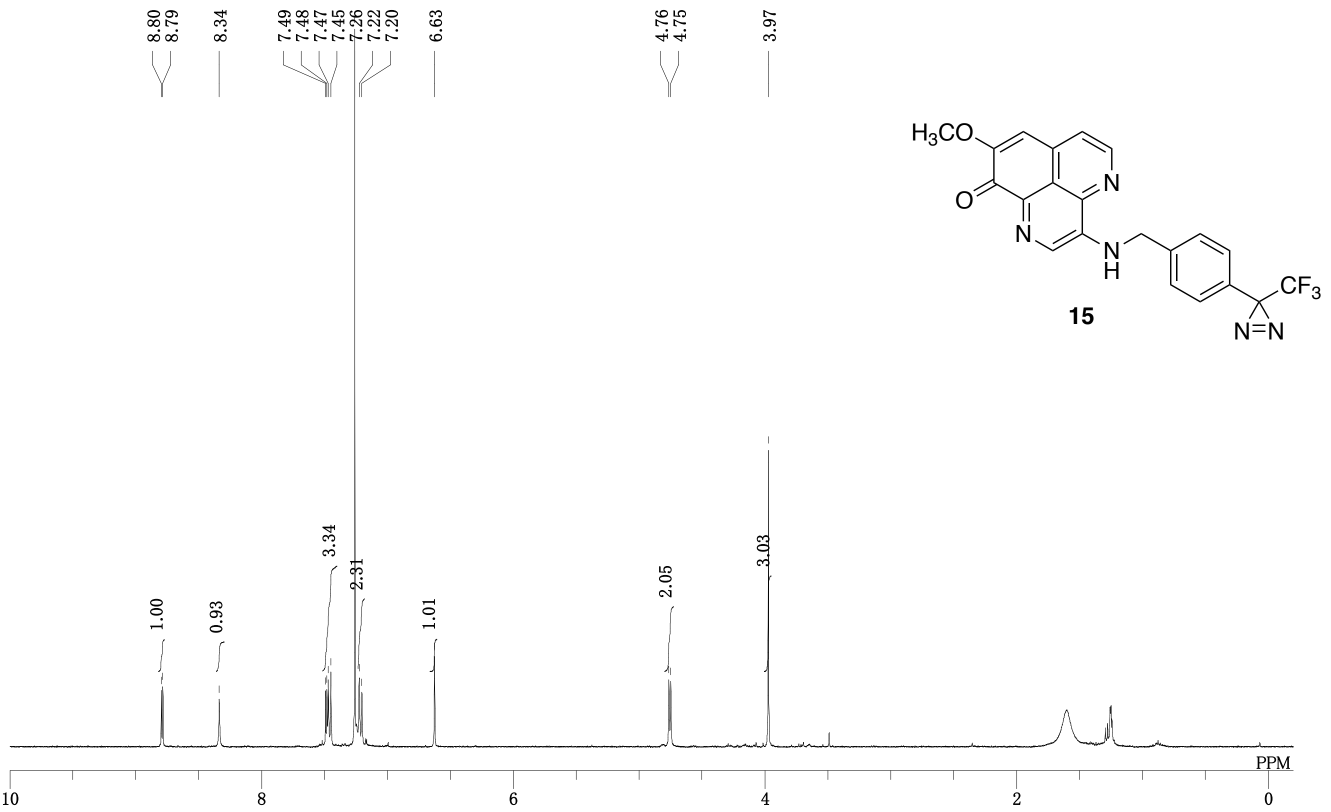

Figure S16. ^{13}C NMR spectrum of **15**

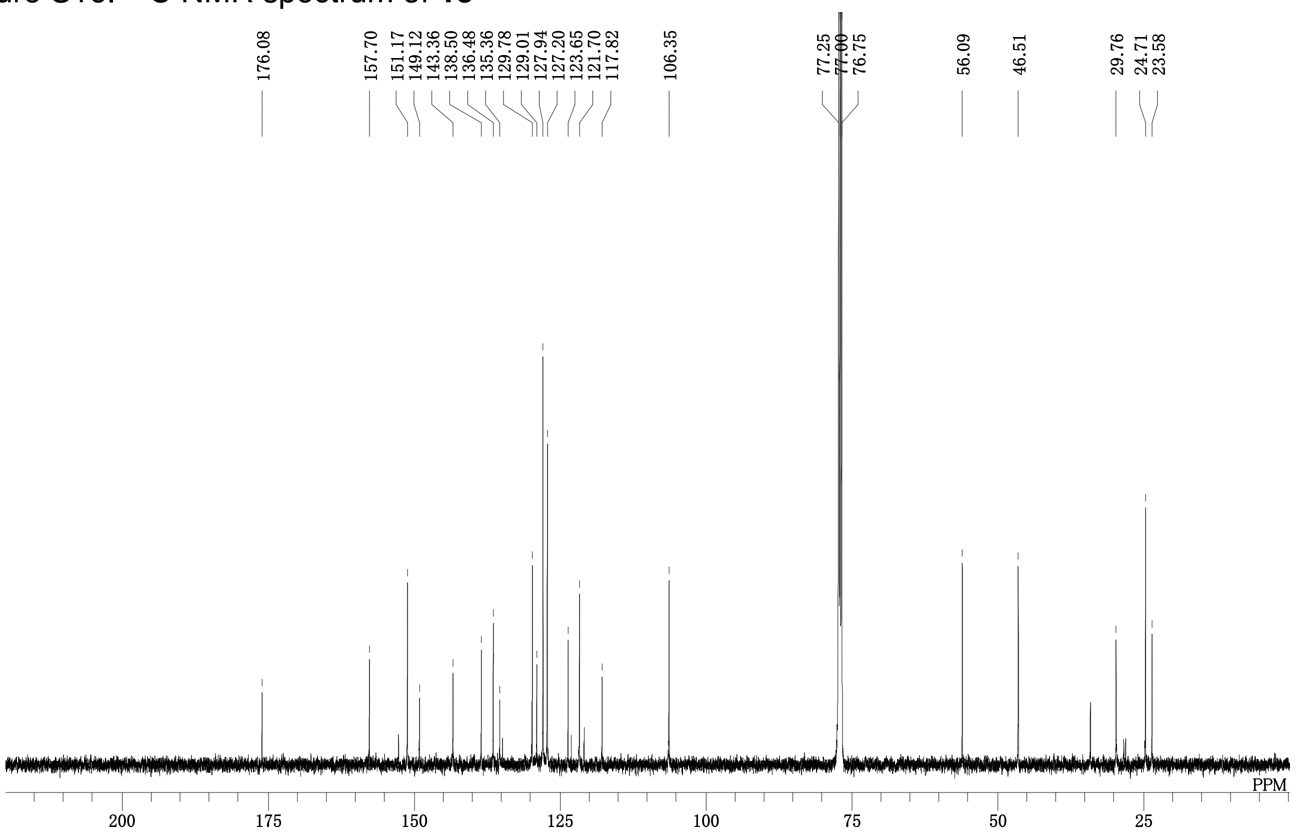

Figure S17. ^{1}H NMR spectrum of **16**

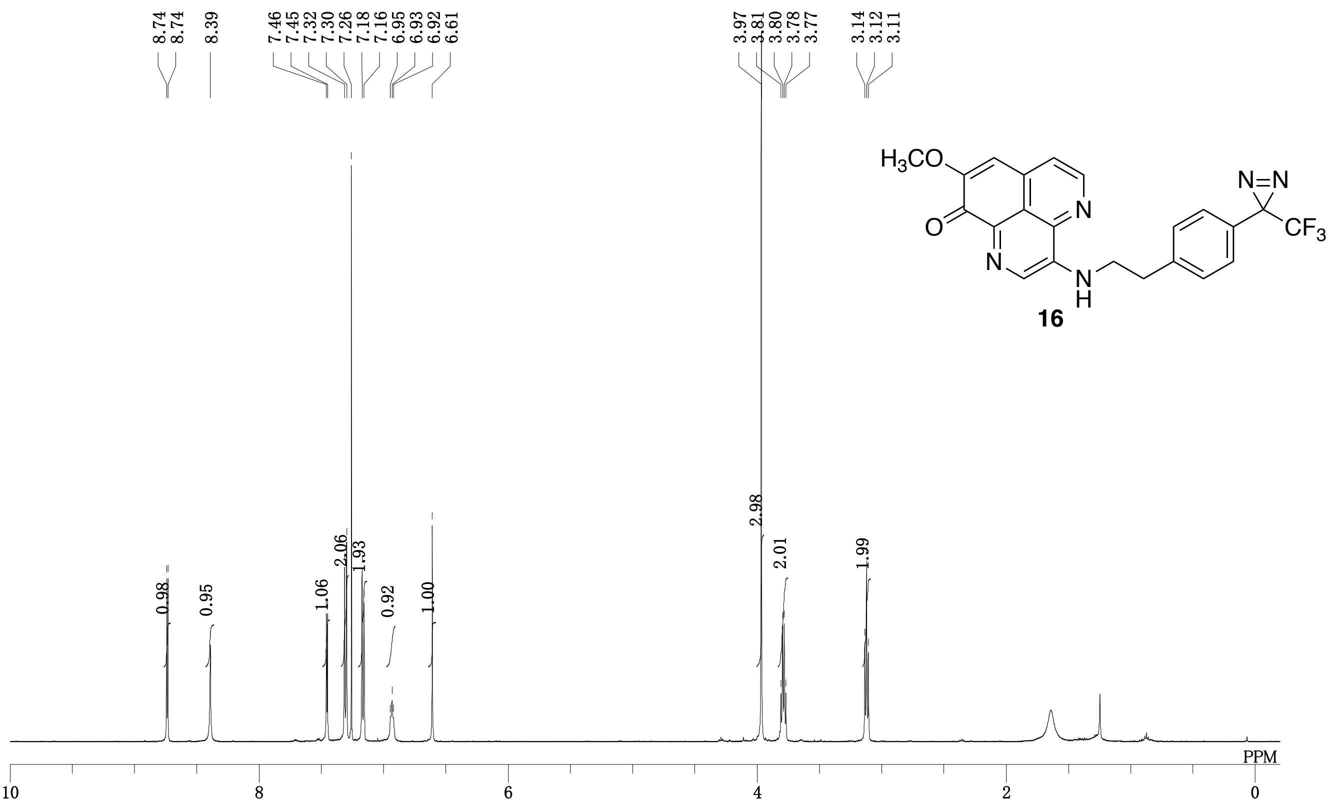

Figure S18. ^{13}C NMR spectrum of **16**

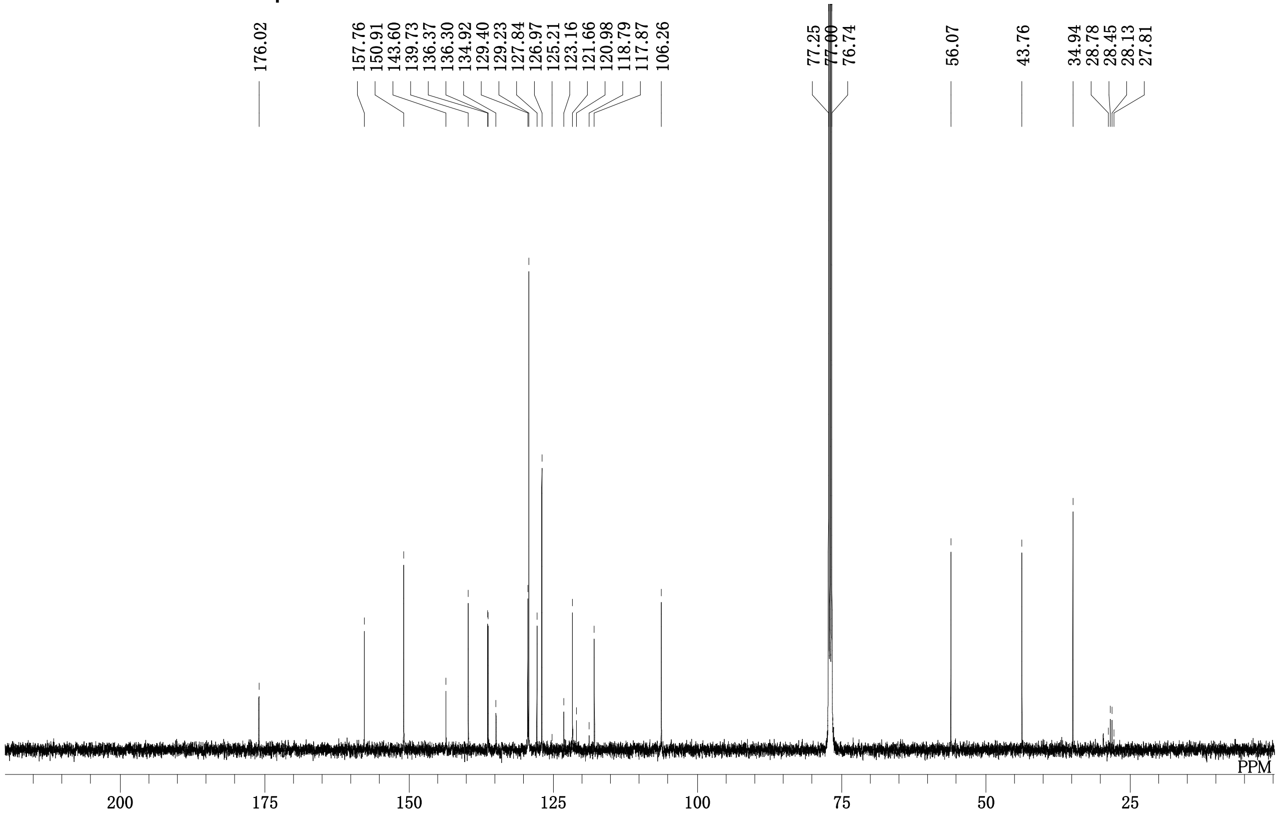

Figure S19. ^{1}H NMR spectrum of **17**

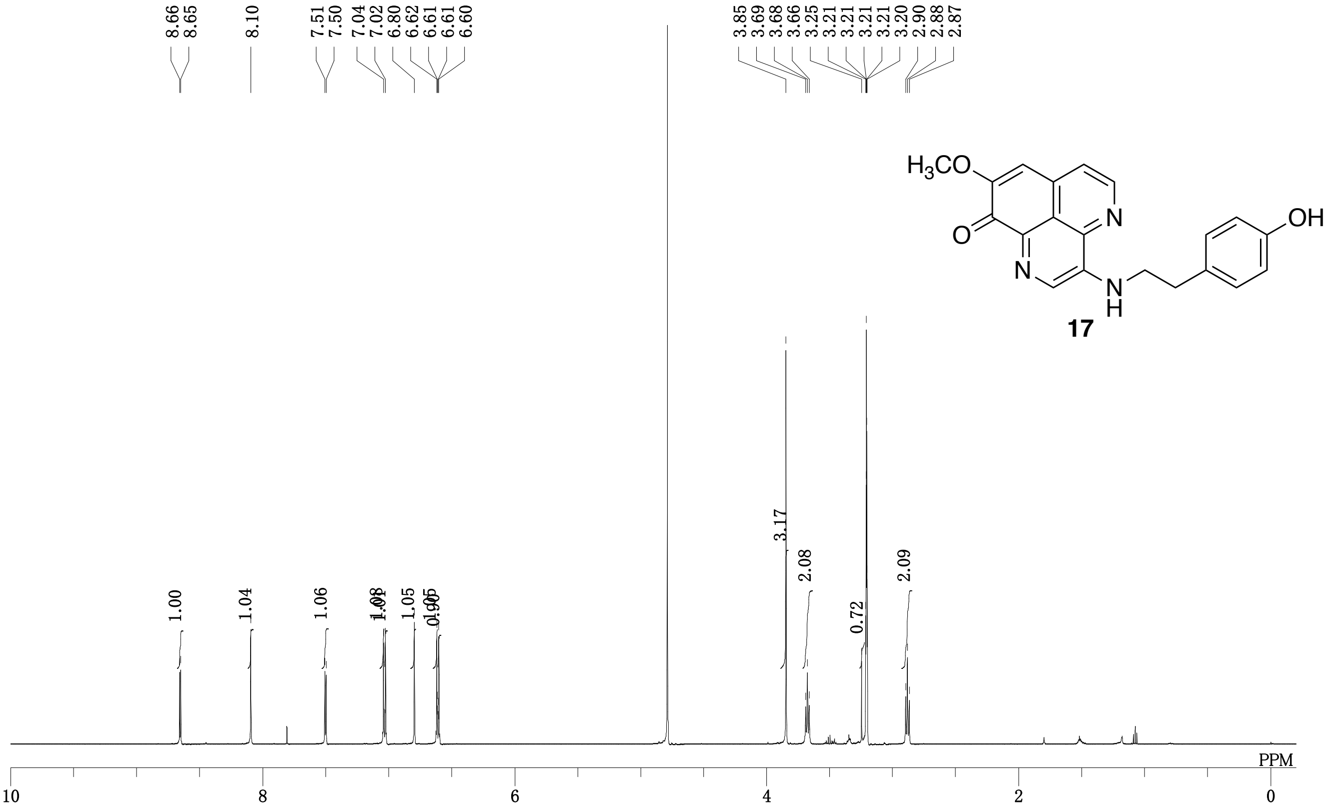

Figure S20. ^{13}C NMR spectrum of **17**

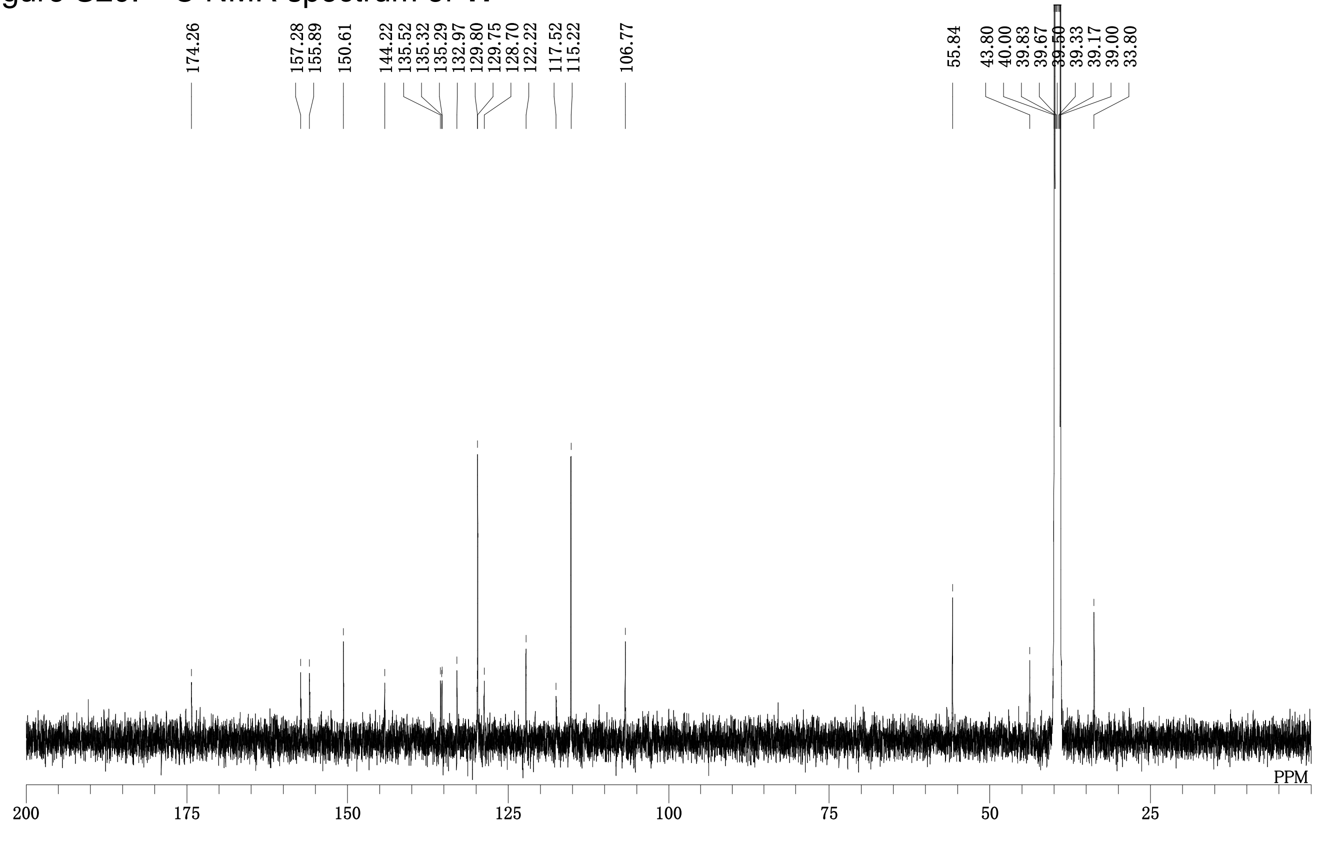

Figure S21. ^{1}H NMR spectrum of **7**

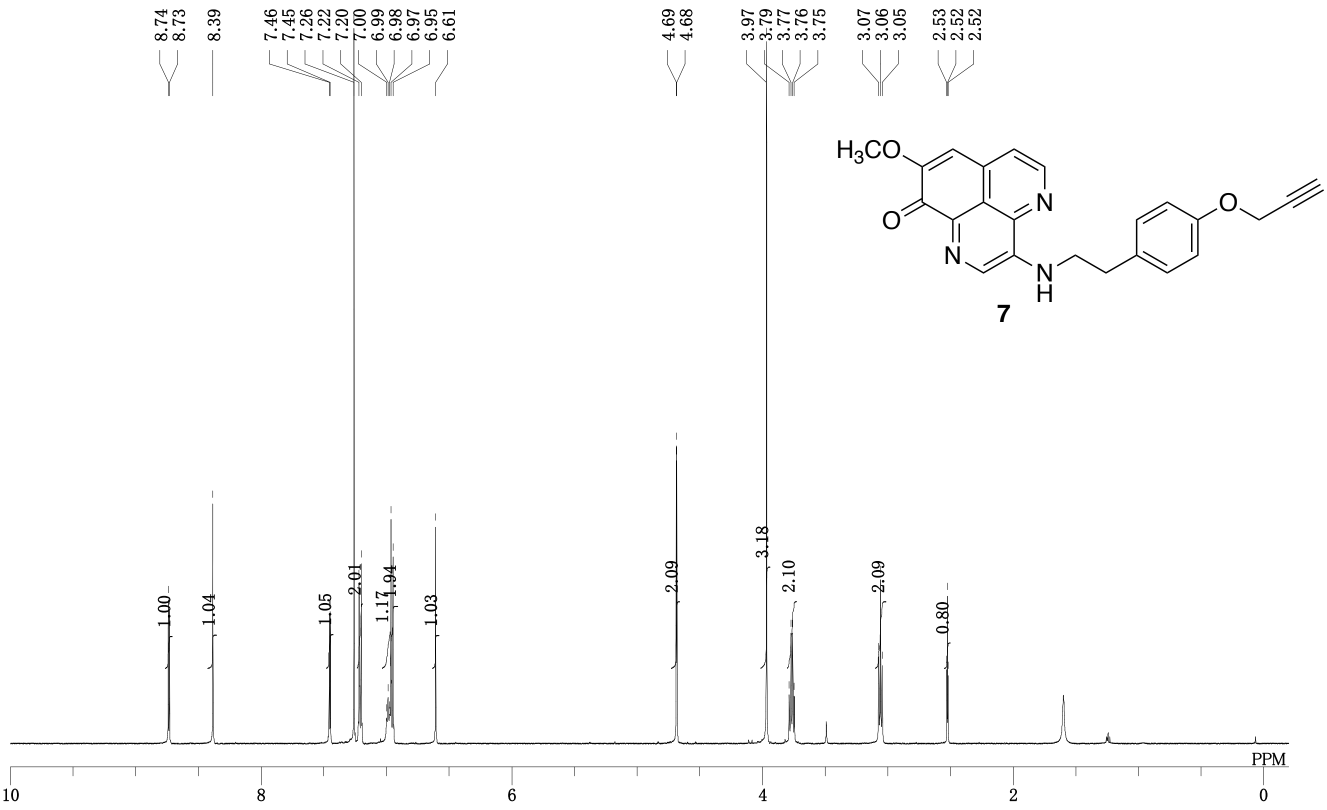

Figure S22. ^{13}C NMR spectrum of **7**

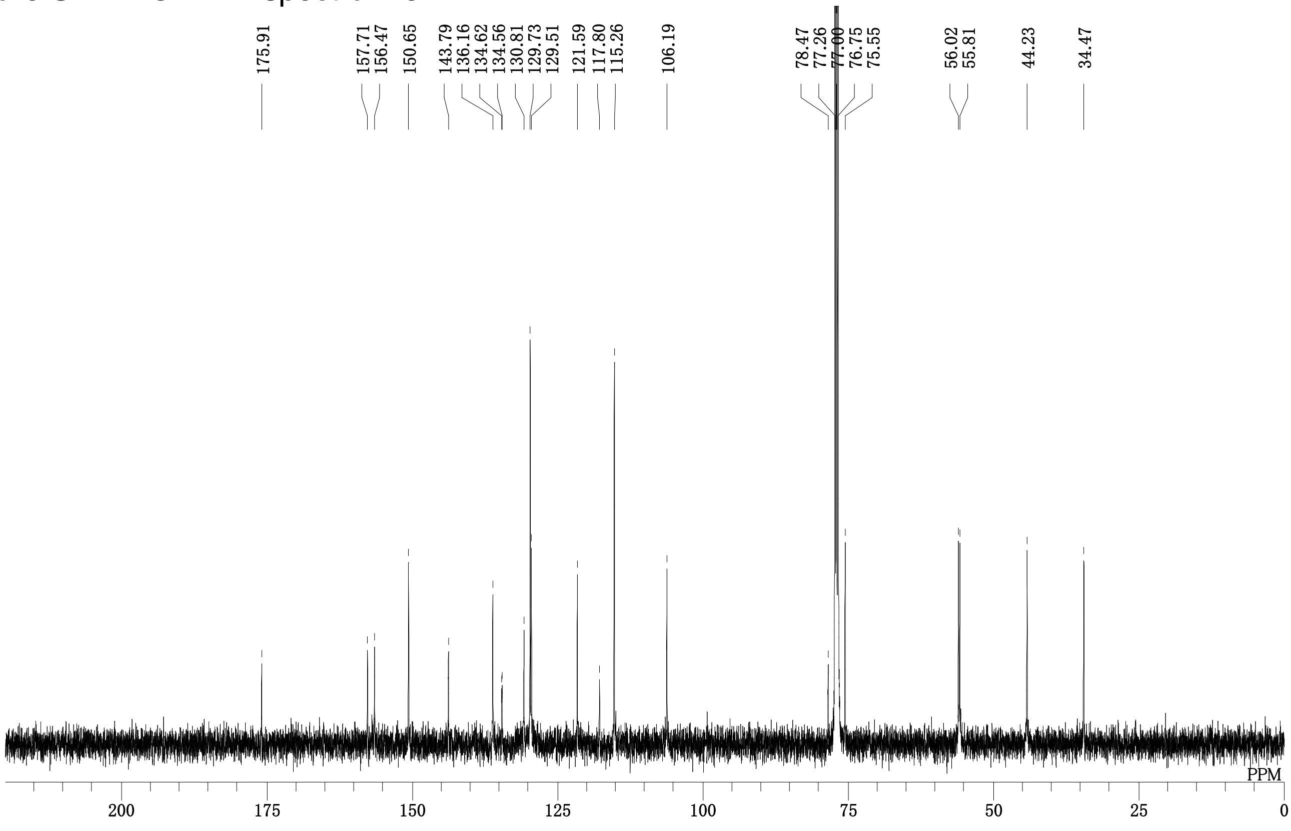

Figure S23. ^{1}H NMR spectrum of **12**

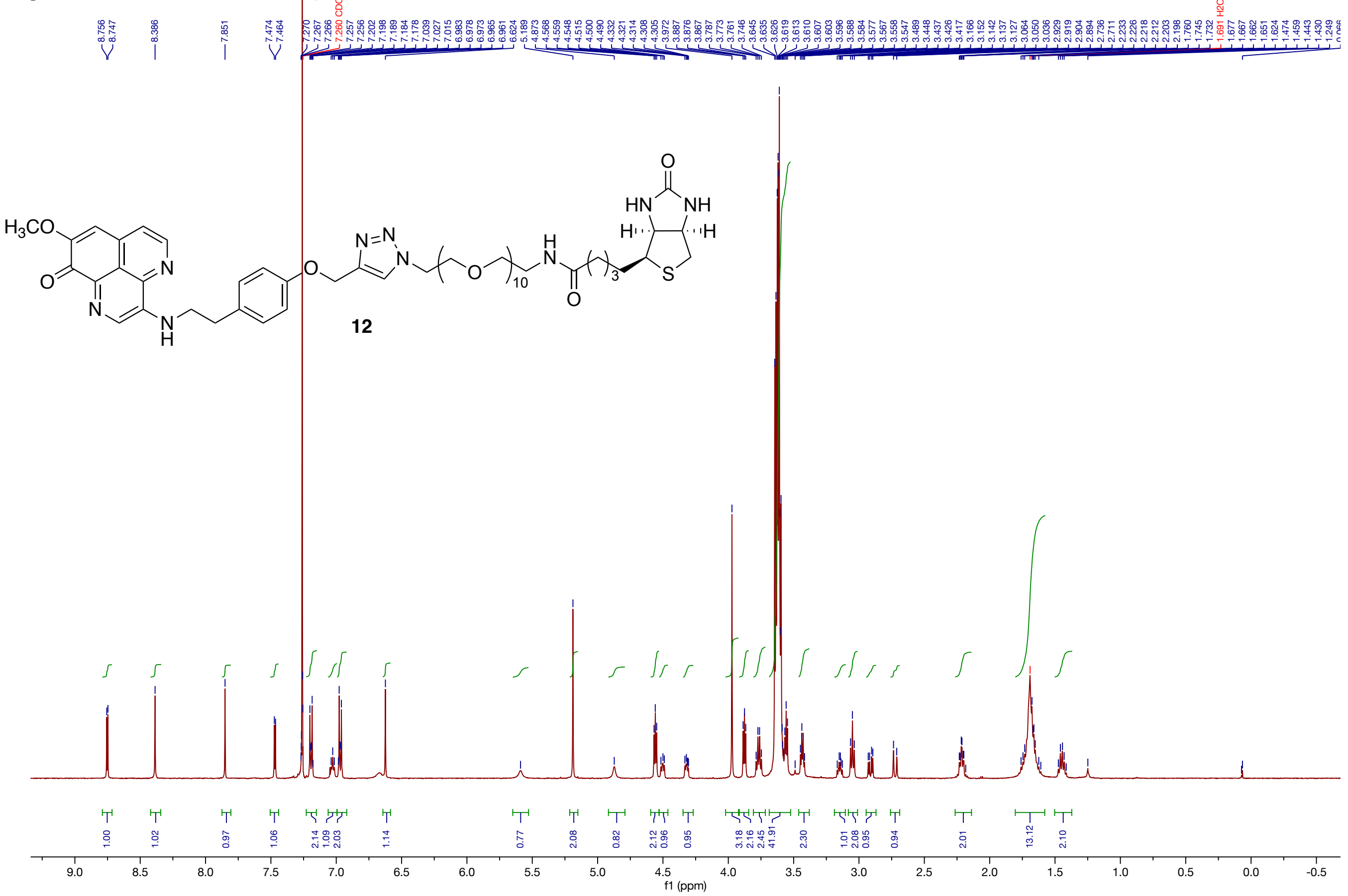

Figure S24. ^{1}H NMR spectrum of **13**

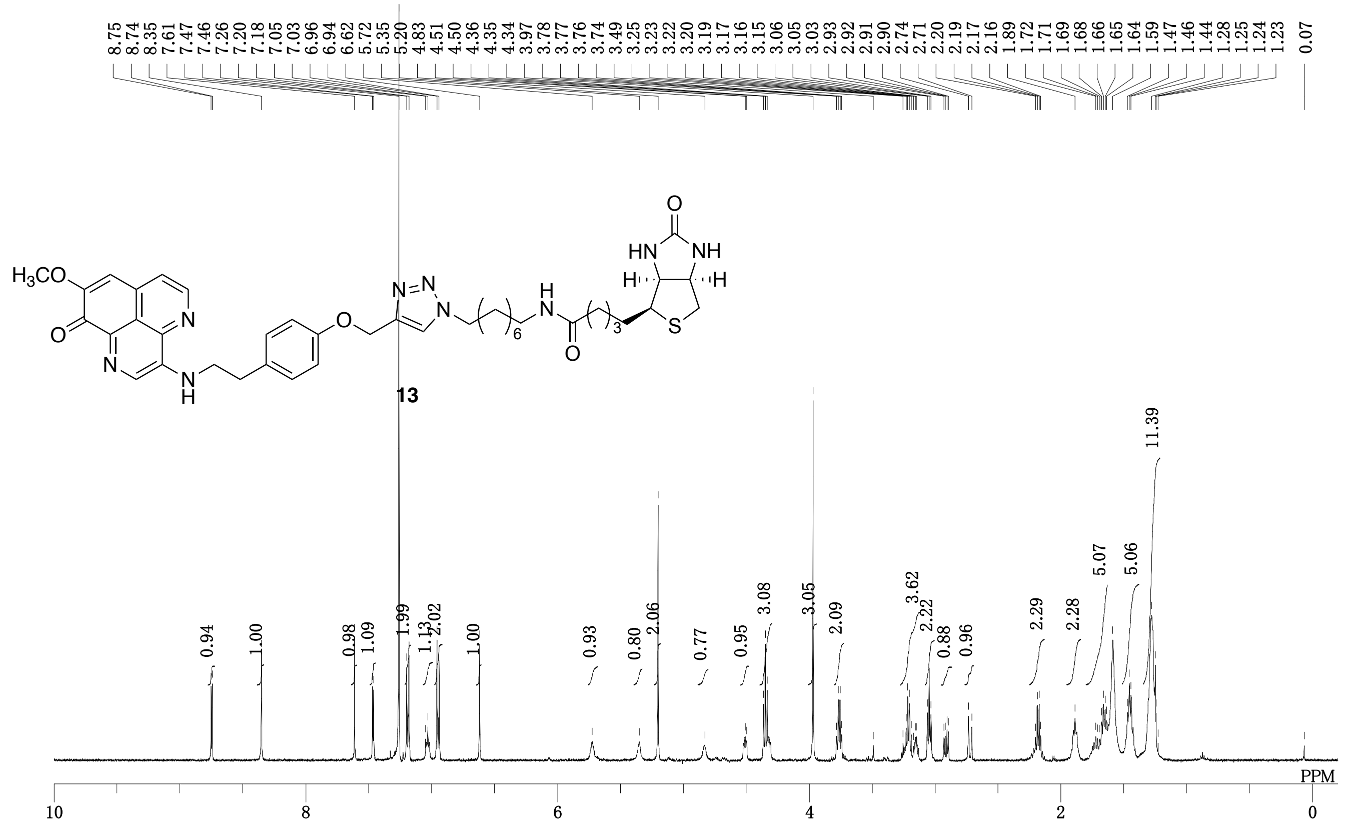

Figure S25. ^{1}H NMR spectrum of **8**

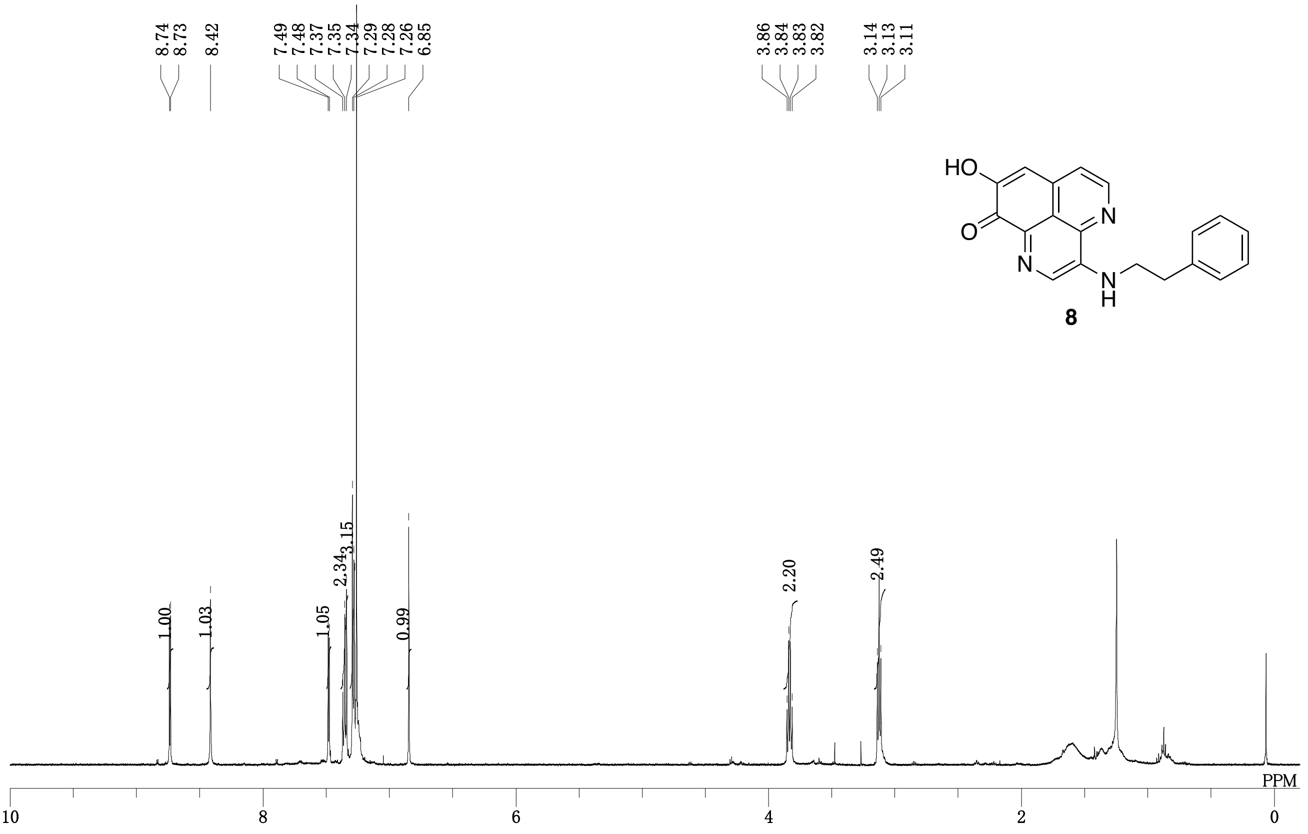

Figure S26. ^{13}C NMR spectrum of **8**

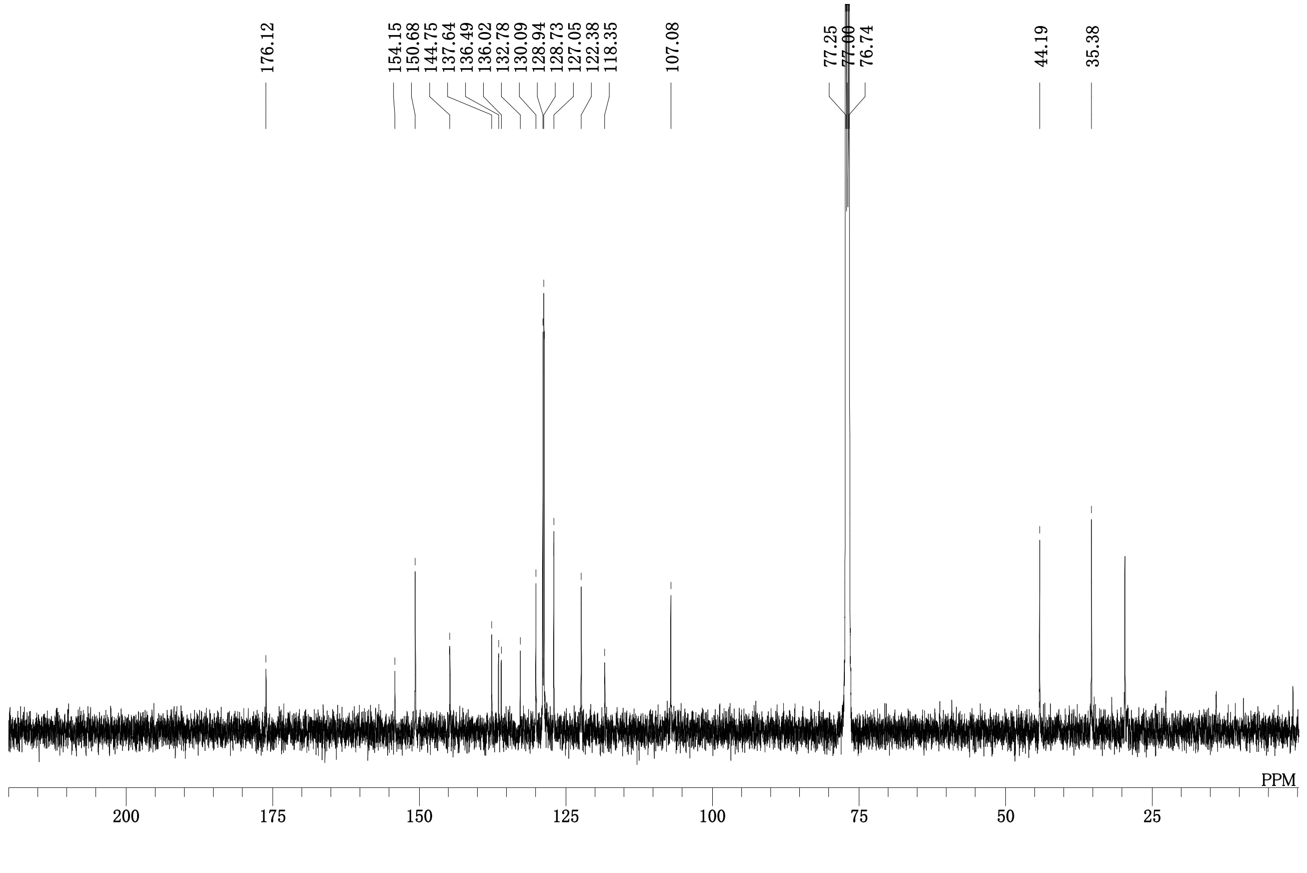

Figure S27. ^{1}H NMR spectrum of **9**

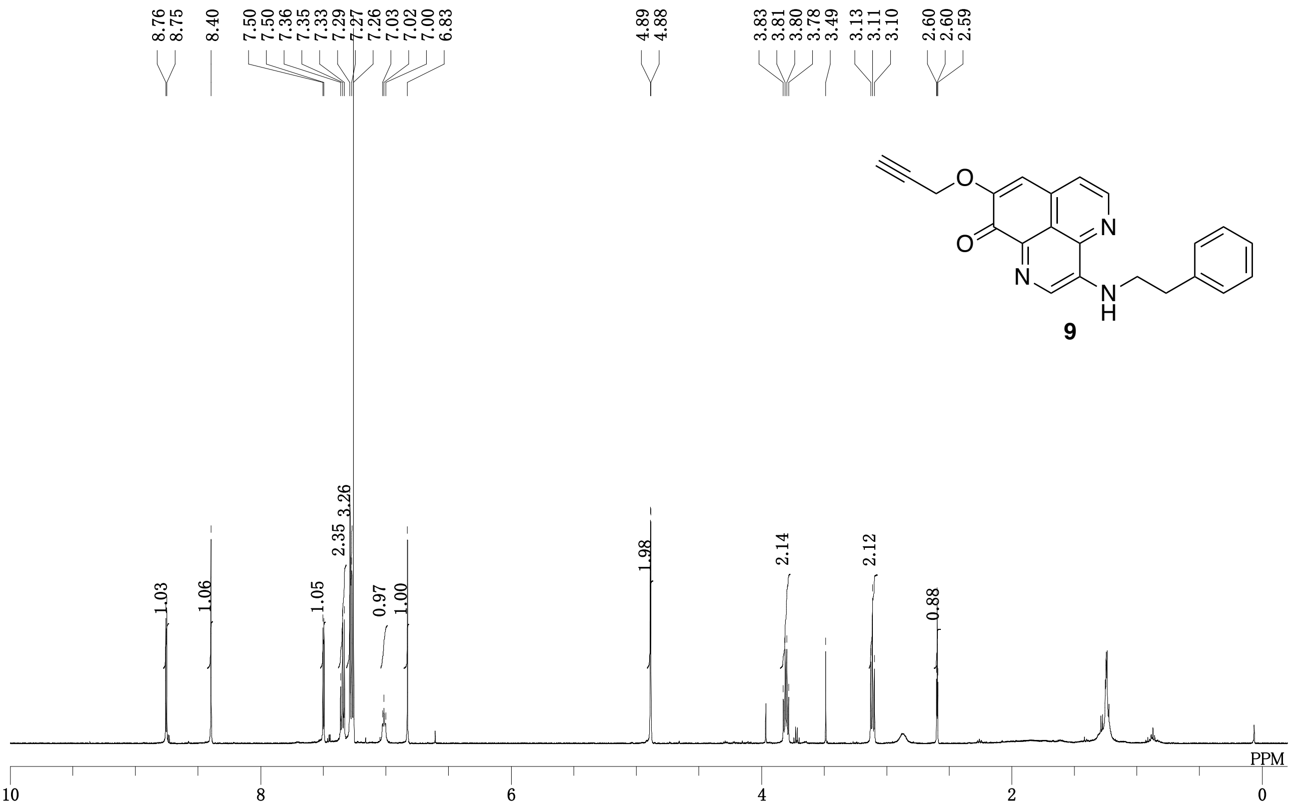

Figure S28. ^{13}C NMR spectrum of **9**

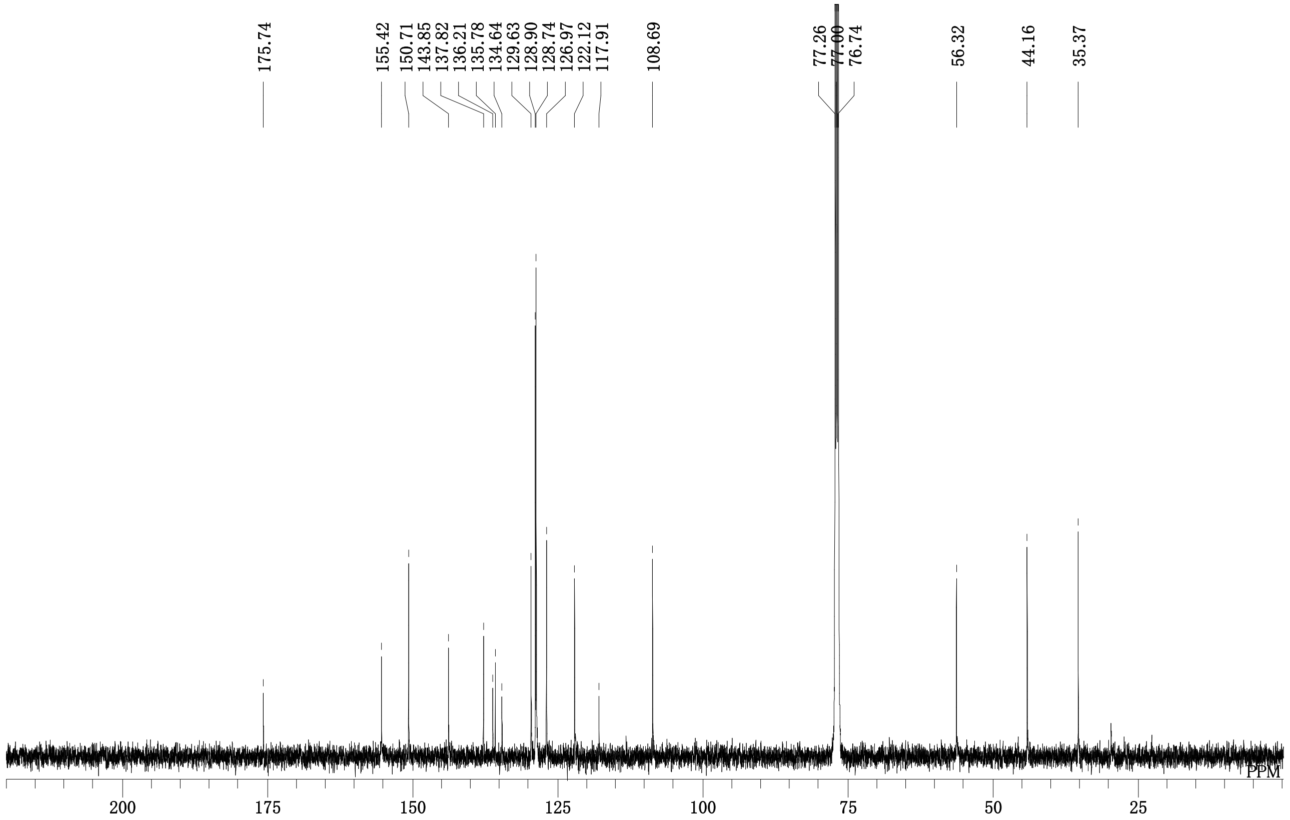

Figure S29. ^{1}H NMR spectrum of **14**

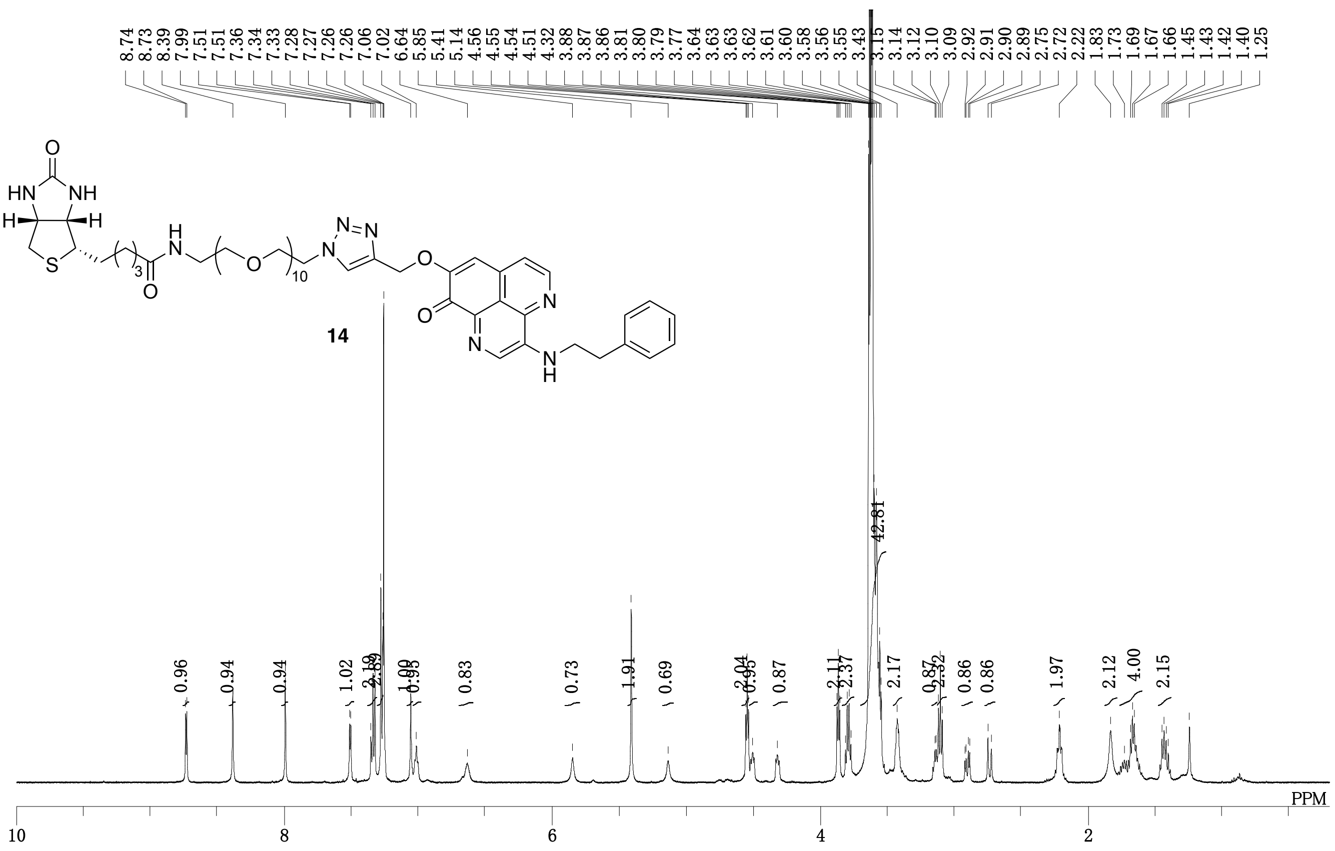

Figure S30. ^{1}H NMR spectrum of 1-(4-(2-((*tert*-Butyldimethylsilyl)oxy)ethyl)phenyl)-2,2,2-trifluoroethanone *O*-tosyl oxime

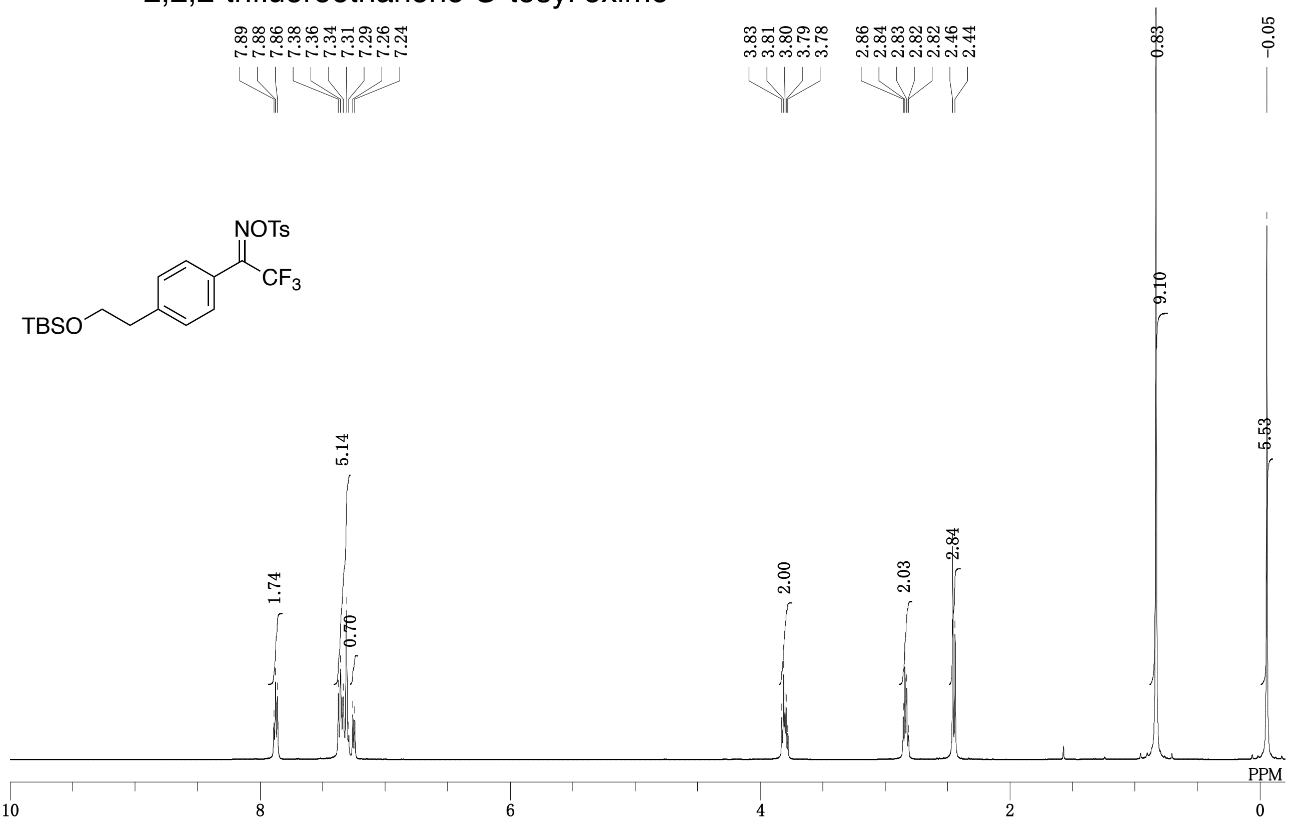

Figure S31. ^{13}C NMR spectrum of 1-(4-(2-((*tert*-Butyldimethylsilyl)oxy)ethyl)phenyl)-2,2,2-trifluoroethanone *O*-tosyl oxime

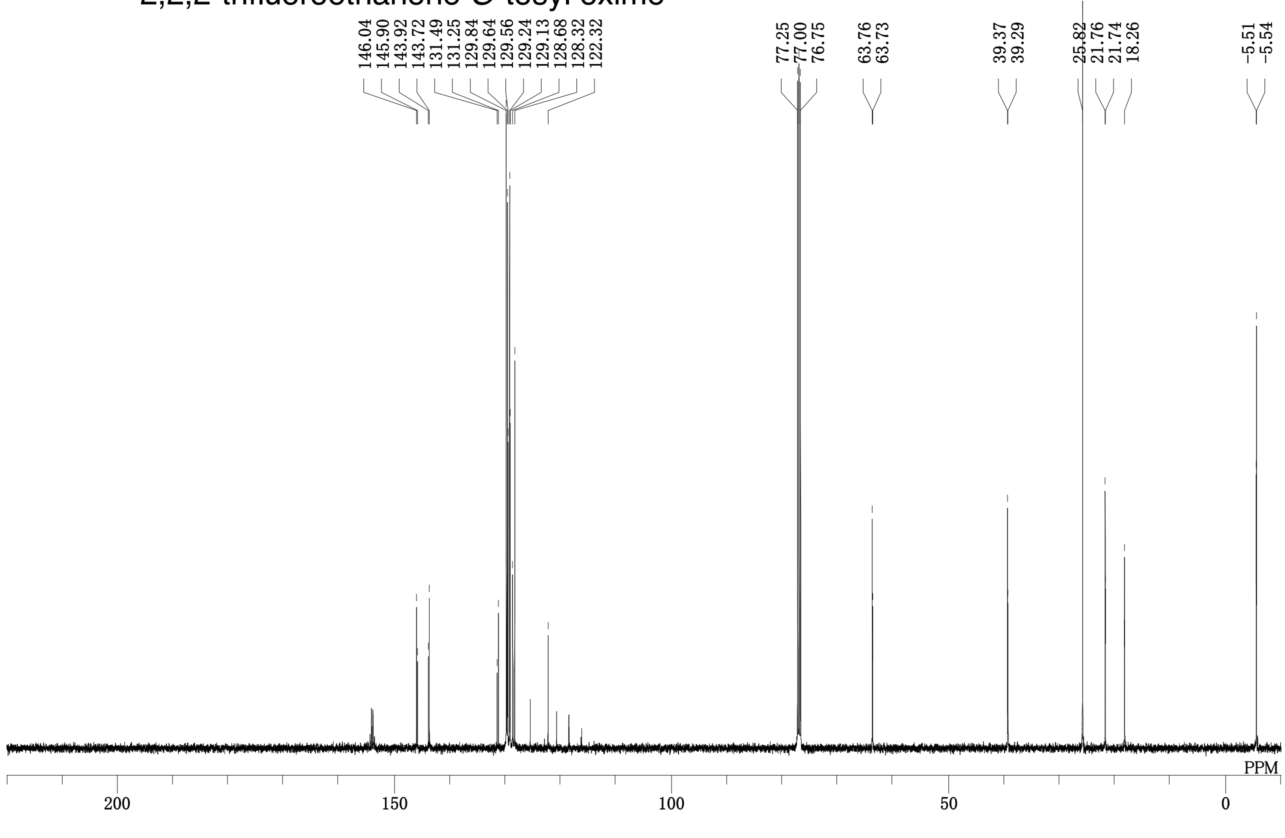

Figure S32. ^{1}H NMR spectrum of 3-(4-(2-((*tert*-Butyldimethylsilyl)oxy)ethyl)phenyl)-3-(trifluoromethyl)diaziridine

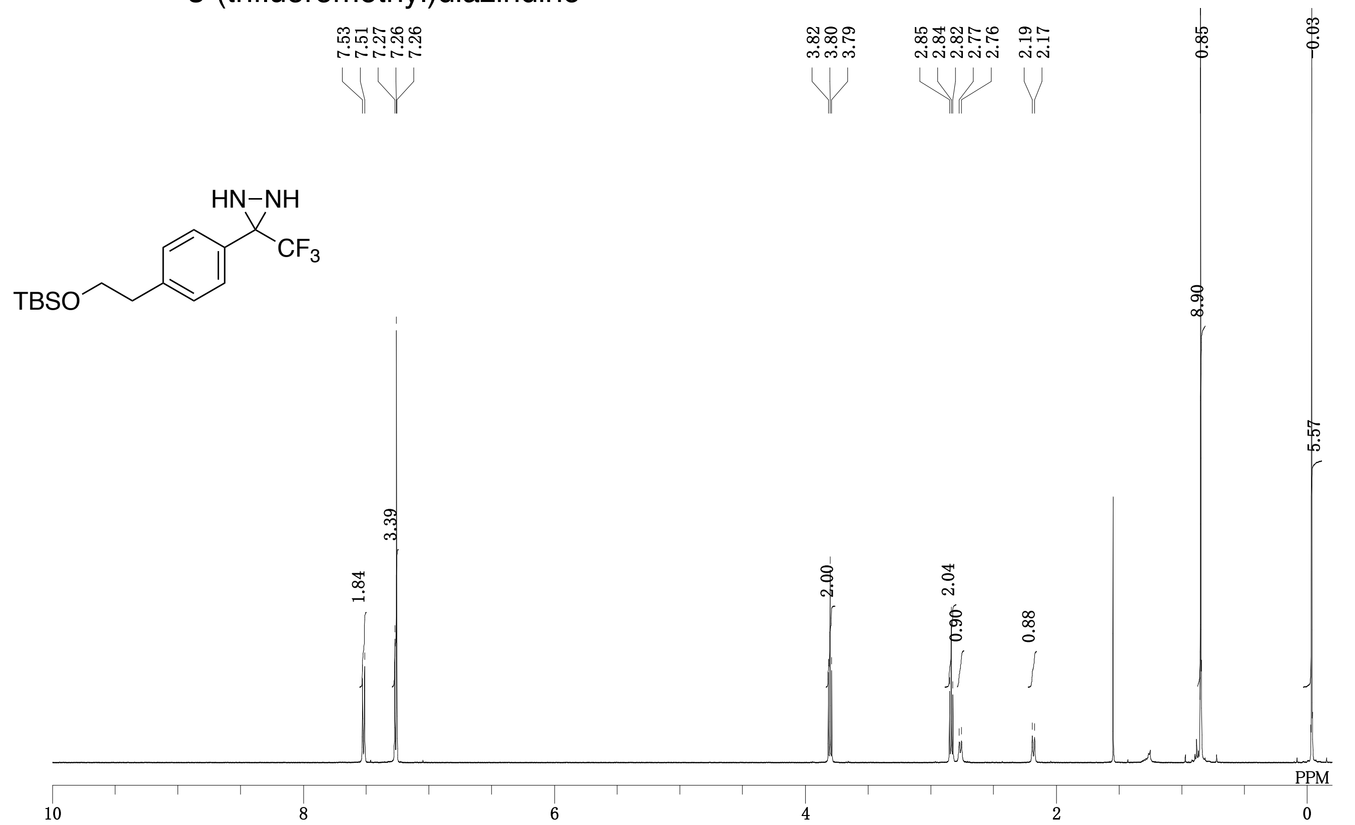

Figure S33. ^{13}C NMR spectrum of 3-(4-(2-((*tert*-Butyldimethylsilyl)oxy)ethyl)phenyl)-3-(trifluoromethyl)diaziridine

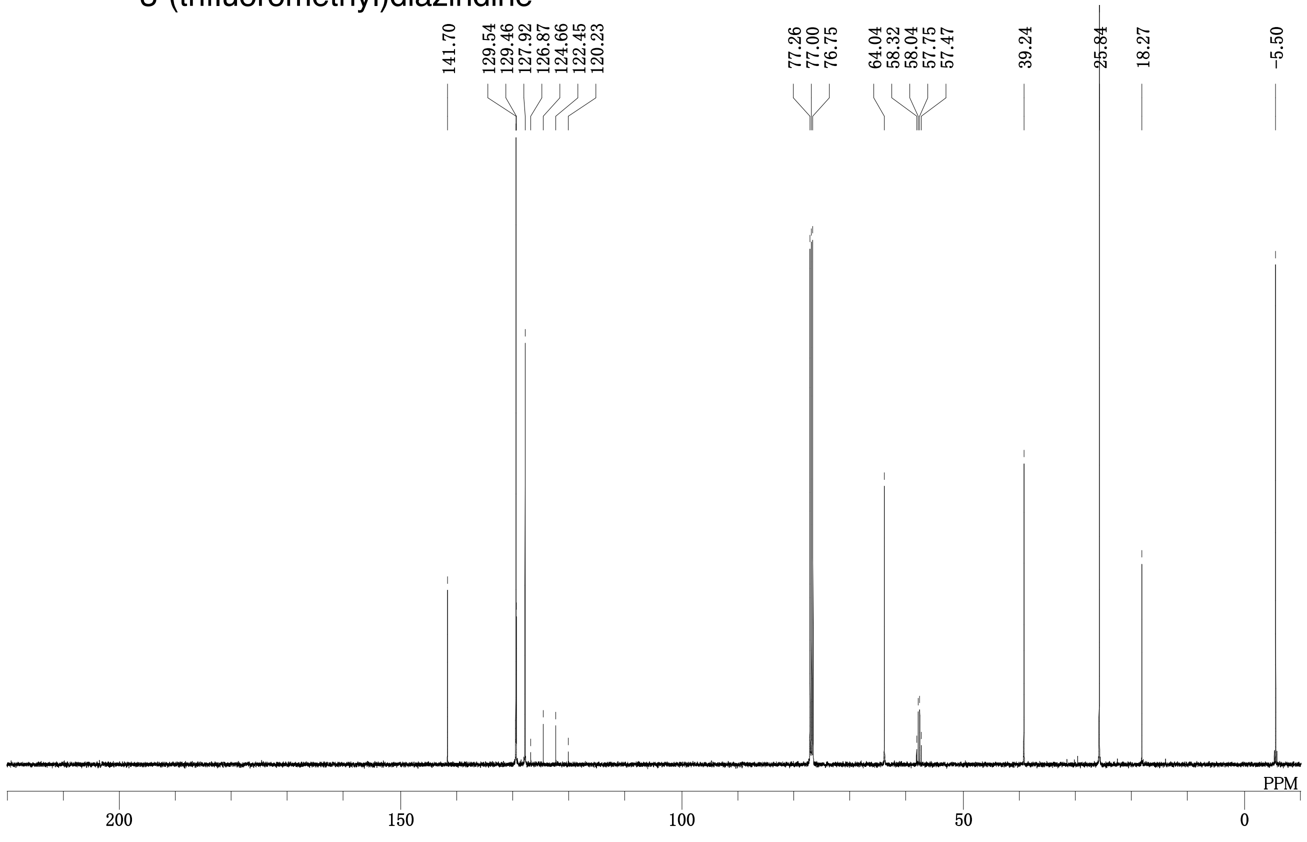

Figure S34. ^{1}H NMR spectrum of 3-(4-(2-((*tert*-Butyldimethylsilyl)oxy)ethyl)phenyl)-3-(trifluoromethyl)-3*H*-diazirine

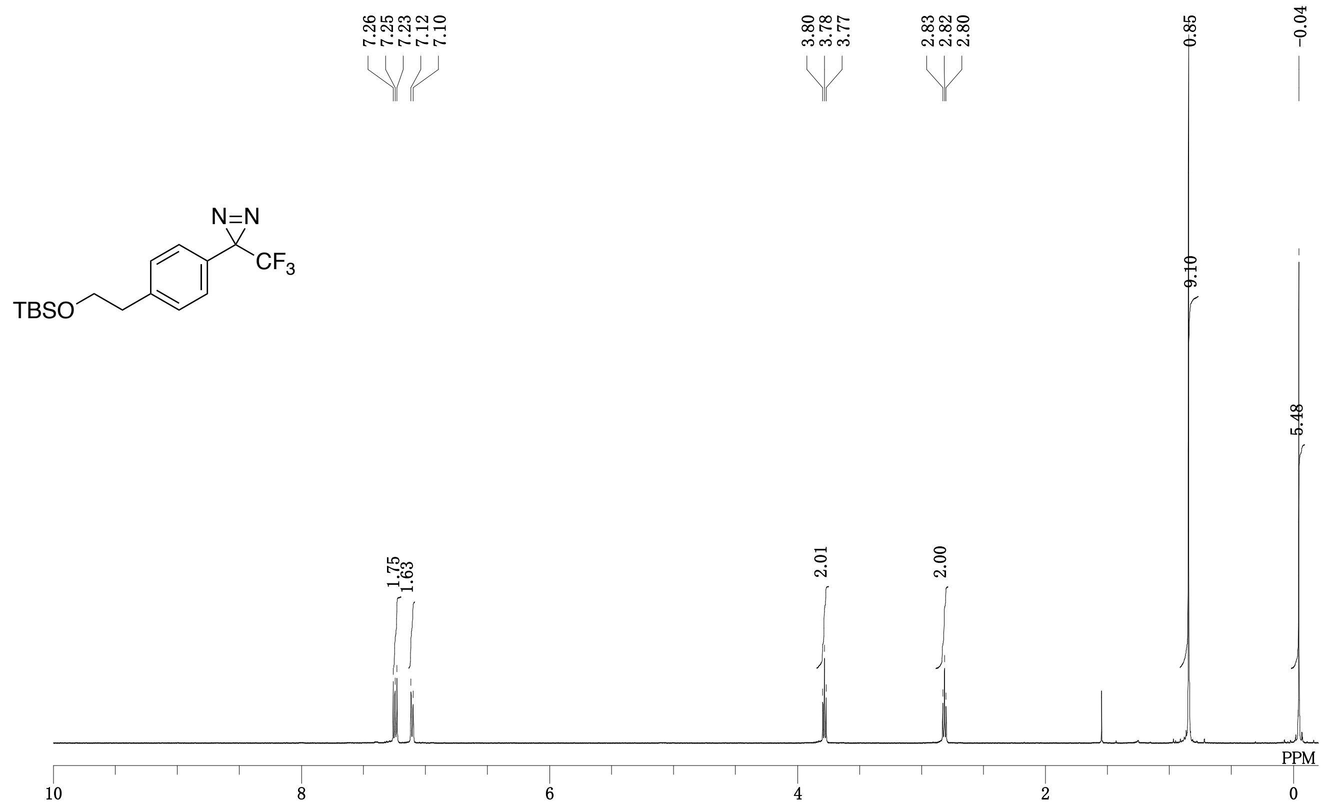

Figure S35. ^{13}C NMR spectrum of 3-(4-(2-((*tert*-Butyldimethylsilyl)oxy)ethyl)phenyl)-3-(trifluoromethyl)-3*H*-diazirine

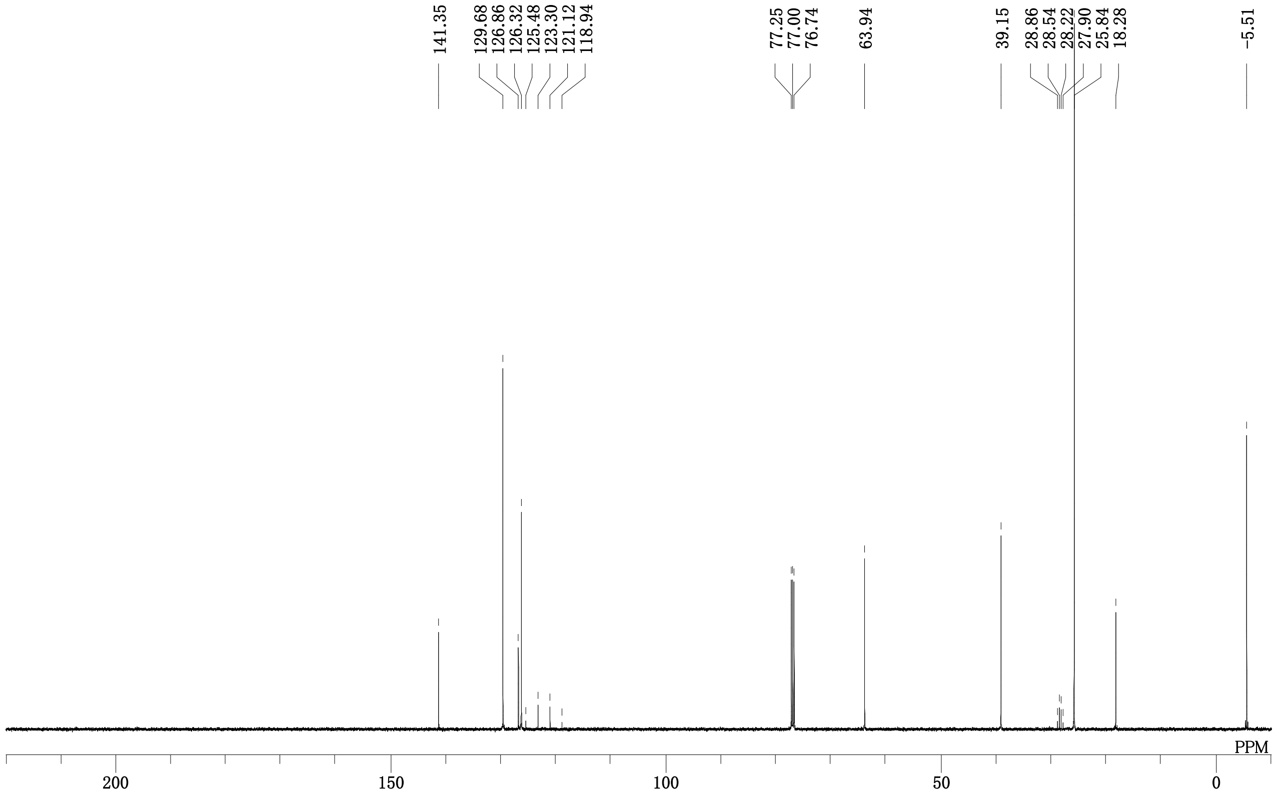

Figure S36. ^{1}H NMR spectrum of 2-(4-(3-(Trifluoromethyl)-3*H*-diazirin-3-yl)phenyl)ethanol

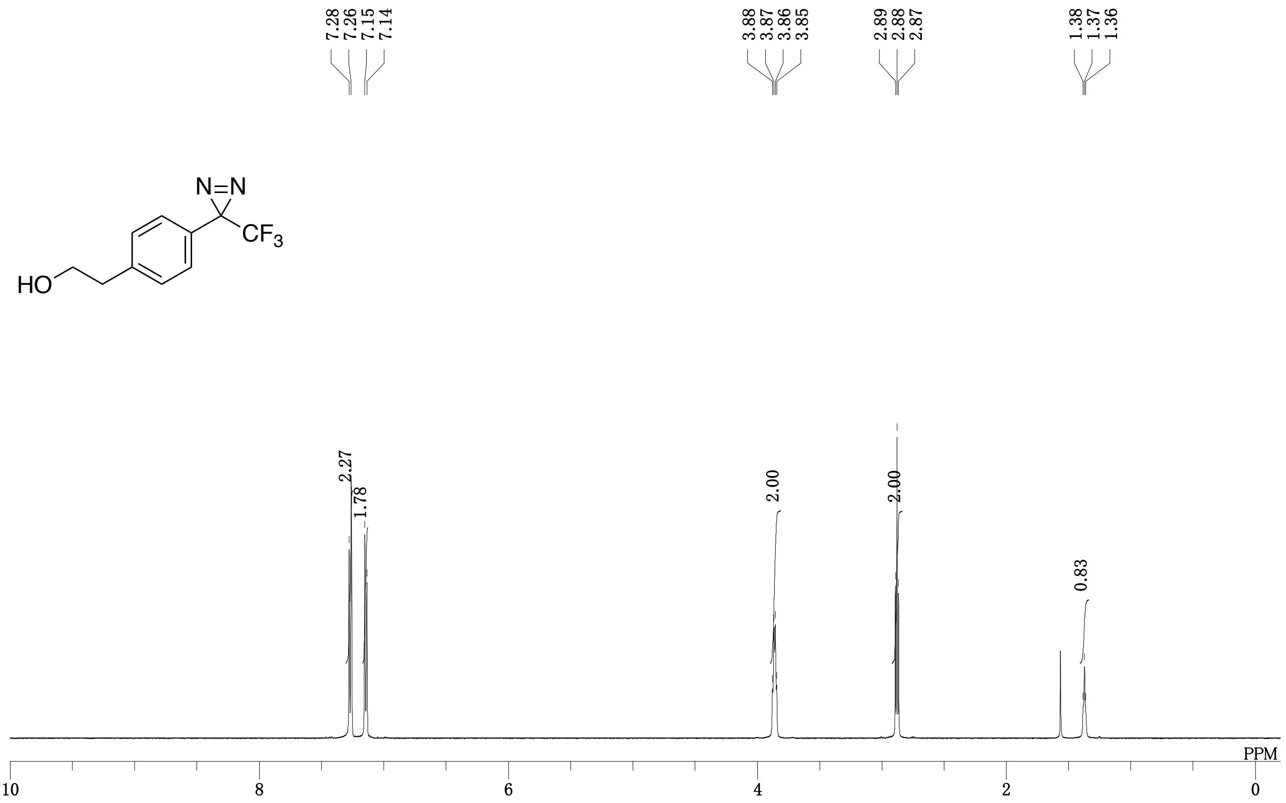

Figure S37. ^{13}C NMR spectrum of 2-(4-(3-(Trifluoromethyl)-3*H*-diazirin-3-yl)phenyl)ethanol

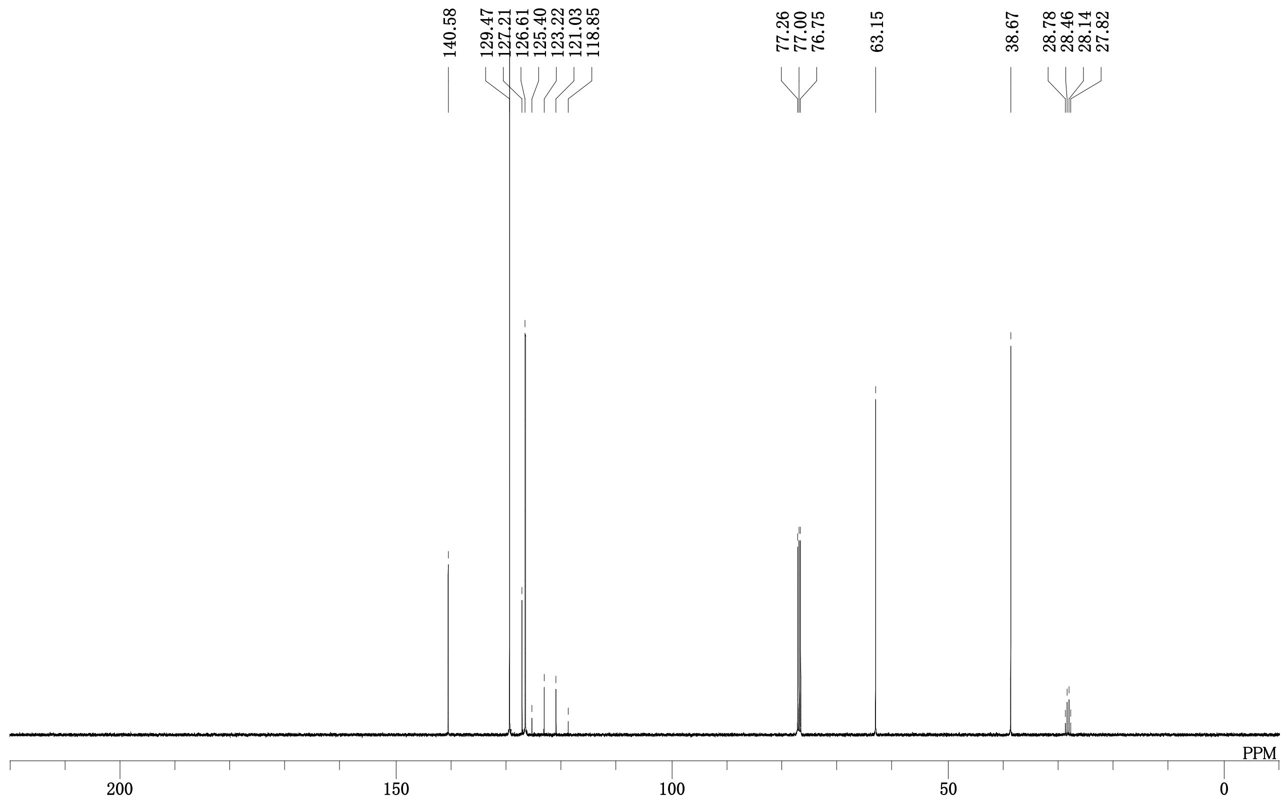

Figure S38. ^{1}H NMR spectrum of 2-(4-(3-(Trifluoromethyl)-3*H*-diazirin-3-yl)phenyl)acetic acid

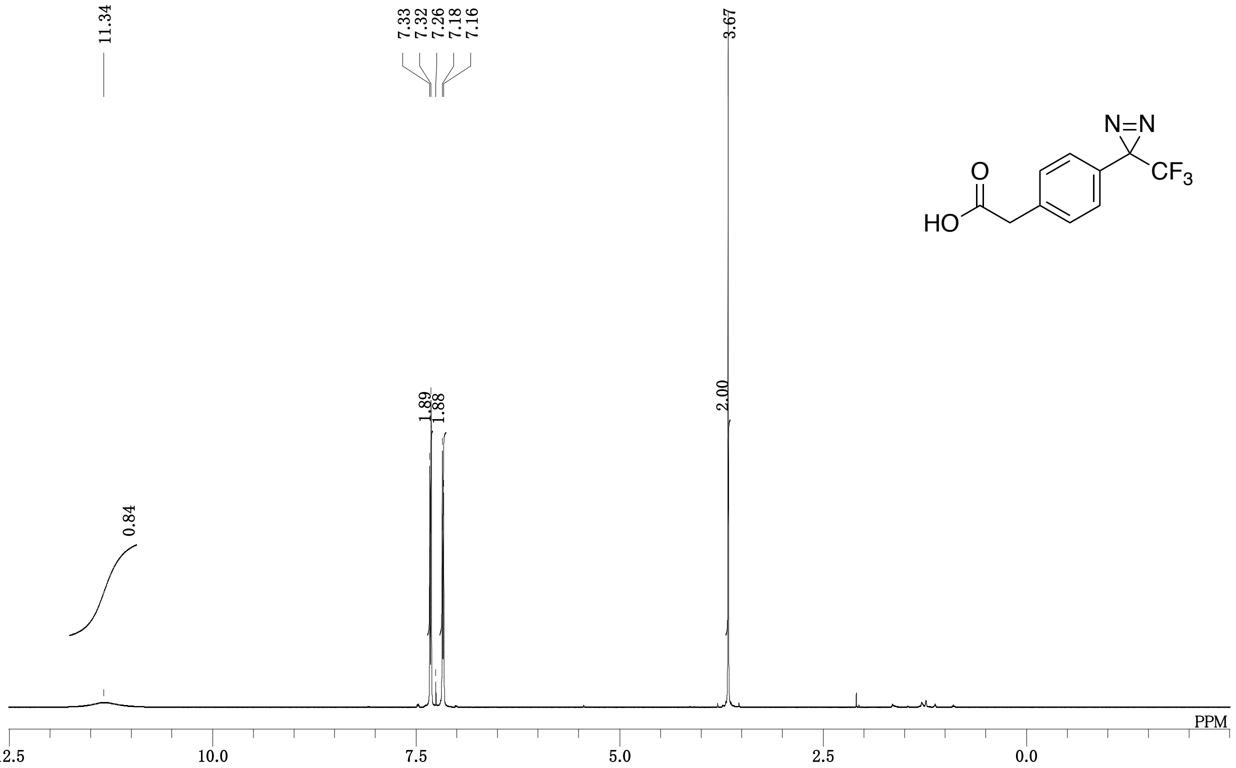

Figure S39. ^{13}C NMR spectrum of 2-(4-(3-(Trifluoromethyl)-3*H*-diazirin-3-yl)phenyl)acetic acid

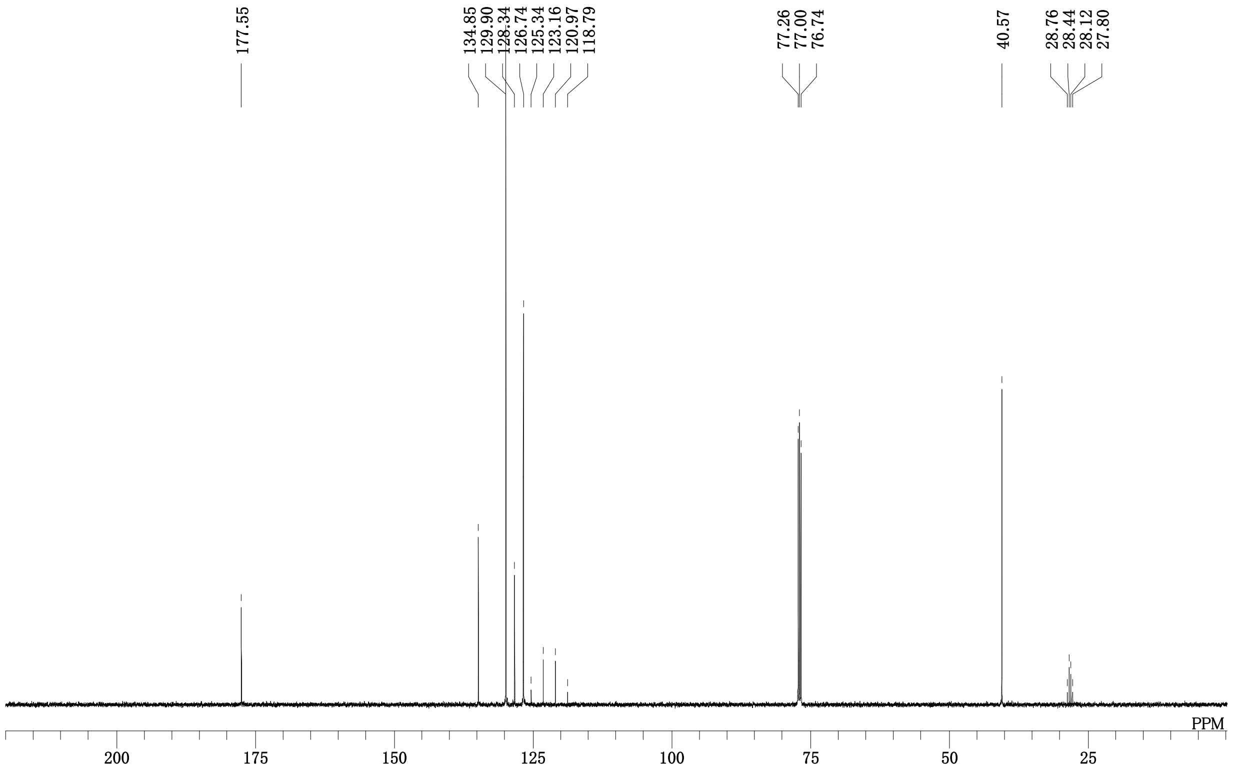

Supplement: Supplementary file 1 [file marinedrugs-20-00098-s001.zip › marinedrugs-1526465-supplementary.pdf]
